# Highly Active Oxygen Evolution Integrating with Highly Selective CO_2_-to-CO Reduction

**DOI:** 10.1007/s40820-025-01688-2

**Published:** 2025-03-13

**Authors:** Chaowei Wang, Laihong Geng, Yingpu Bi

**Affiliations:** 1 State Key Laboratory for Oxo Synthesis and Selective Oxidation, National Engineering Research Center for Fine Petrochemical Intermediates, Lanzhou Institute of Chemical Physics, Chinese Academy of Sciences, Lanzhou, 730000 People’s Republic of China; 2 University of Chinese Academy of Sciences, Beijing, 100049 People’s Republic of China; 3 Gansu Research Institute of Chemical Industry Co., Ltd, Lanzhou, 730000 People’s Republic of China

**Keywords:** Photosynthesis, Oxygen evolution, CO_2_ reduction, Photoanode, Single-atom Co-N_5_

## Abstract

Artificial carbon fixation is a promising pathway for achieving the carbon cycle and environment remediation. However, the sluggish kinetics of oxygen evolution reaction (OER) and poor selectivity of CO_2_ reduction seriously limited the overall conversion efficiencies of solar energy to chemical fuels. Herein, we demonstrated a facile and feasible strategy to rationally regulate the coordination environment and electronic structure of surface-active sites on both photoanode and cathode. More specifically, the defect engineering has been employed to reduce the coordination number of ultrathin FeNi catalysts decorated on BiVO_4_ photoanodes, resulting in one of the highest OER activities of 6.51 mA cm^−2^ (1.23 *V*_RHE_, AM 1.5G). Additionally, single-atom cobalt (II) phthalocyanine anchoring on the N-rich carbon substrates to increase Co–N coordination number remarkably promotes CO_2_ adsorption and activation for high selective CO production. Their integration achieved a record activity of 109.4 μmol cm^−2^ h^−1^ for CO production with a faradaic efficiency of > 90%, and an outstanding solar conversion efficiency of 5.41% has been achieved by further integrating a photovoltaic utilizing the sunlight (> 500 nm).

**Supplementary Information:**

The online version contains supplementary material available at 10.1007/s40820-025-01688-2.

## Highlights

- Rational regulation of the coordination environment of surface-active sites on both photoanode and cathode has been demonstrated.
- Reducing the coordination of FeNi catalysts decorated on BiVO_4_ photoanodes achieves excellent water oxidation activities of 6.51 mA cm^−2^ (1.23 *V*_RHE_, AM 1.5G).
- Single-atom cobalt anchoring on N-rich carbon with increased Co–N coordination remarkably promotes CO_2_ reduction to CO.

**Supplementary Information:**

The online version contains supplementary material available at 10.1007/s40820-025-01688-2.

## Graphical abstract

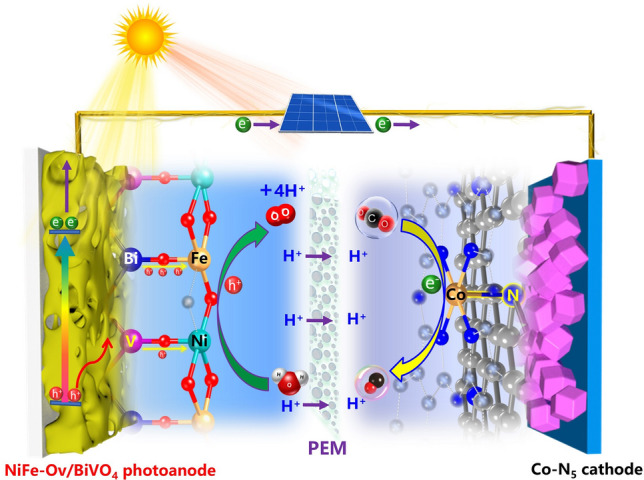

## Introduction

The artificial carbon fixation is one of the greatest challenges for the field of modern chemistry concerning to sustainable energy sources and effective carbon dioxide (CO_2_) mitigation [1–5]. One promising approach involves the coupling of sunlight-driven photoanodes with dark cathodes for achieving oxygen evolution and CO_2_ reduction, presenting a feasible and promising solution to simulate the natural photosynthesis [6–10]. In this typical configuration, a highly active photoanode is essential for significantly reducing the energy barrier of oxygen evolution reaction (OER) to release protons and electrons, maximizing the efficiency of the overall solar energy conversion system [11–17]. Among diverse photoanode materials, bismuth vanadate (BiVO_4_) has attracted considerable attentions benefiting from its appropriate bandgap (2.4 eV), suitable valence edge positions, and low onset potential. To further promote OER activities, the rational construction of transition metal oxides or (oxy)hydroxides, especially for VIII metals (Fe, Co, Ni), on BiVO_4_ photoanode surfaces has attracted considerable attentions in recent years [18–21]. For example, Choi et al. [22] reported the electrodeposition of FeOOH/NiOOH dual catalysts on BiVO_4_ photoelectrodes, and a record-breaking photocurrent (4.5 mA cm^−2^ at 1.23 *V*_RHE_) has been achieved. Domen et al. [23] deposited NiFe bimetallic catalysts on BiVO_4_ photoanodes for improving the PEC activities up to 4.2 mA cm^−2^ at 1.23 *V*_RHE_. Our group previously demonstrated the synergy between iron and nickel of FeNi oxyhydroxides significantly improved the PEC water oxidation properties with 5.8 mA cm^−2^ at 1.23 *V*_RHE_ for BiVO_4_ photoanodes [24]. Although diverse strategies have been extensively reported to decorate NiFe catalysts on BiVO_4_ photoelectrodes, the intrinsic roles of coordination environment and electronic structures of surface NiFe active sites on PEC water oxidation behaviors still remain ambiguous until now.

Except for the highly active OER photoanodes for releasing protons and electrons, a highly selective cathode catalyst is also crucial for CO_2_ reduction to target products. Among various CO_2_ reduction products, carbon monoxide (CO) is one of crucial intermediates for numerous industrial applications, especially for Fischer–Tropsch synthesis to produce various liquid hydrocarbons compounds [25–27]. Up to now, the noble metal catalysts such as Au [28], Ag [29–31], and their alloys [32, 33] have been widely studied to improve the selectivity of CO_2_ reduction to CO. Thus, it is highly desirable to explore low-cost and robust cathode catalysts with high CO selectivity for realizing the industrial application for PEC reduction of CO_2_ to CO. Recently, the metal phthalocyanine (MPc) has attracted particular attention for CO_2_ reduction owing to the well-defined coordination environment (metal-N_4_) and unique electronic properties, which could serve as an ideal platform for in-depth studies regarding the potential reaction mechanism and manipulation of terminal products [34–37]. However, suffering from the agglomeration, non-conductivity, and poor CO_2_ adsorption, most of reported MPc demonstrated the unsatisfactory CO_2_ reduction performances [38]. Recently, metal phthalocyanine supported on conductive nanocarbon substrates, such as carbon nanotube [39, 40] and graphene [41, 42], has been demonstrated to optimize the catalytic performances. However, rational regulation of center metal coordination environment and electronic structure has been rarely reported until now, which might be a feasible and promising strategy for significantly promoting the CO_2_ reduction selectivity.

In this work, we firstly demonstrated the rational decrease in oxygen coordination of FeNi catalysts on BiVO_4_ photoanodes to significantly promote the PEC water oxidation performances, and an outstanding photocurrent density of 6.51 mA cm^−2^ at 1.23 *V*_RHE_ has been obtained. The improvement in OER activity should be attributed to surface electron-rich Ni/Fe active sites for accelerating charge separation from bulk BiVO_4_ and hole transfer for water oxidation to release proton and electron. Further coupling with cobalt phthalocyanine (CoPc) supported on N-rich carbon cathode for increasing Co–N coordination to promote CO_2_ adsorption and activation. Under simulated sunlight, an outstanding CO production rate of 109.4 μmol cm^−2^ h^−1^ with an average faradaic efficiency of 90.6% has been obtained in this artificial photosynthetic system. Thereby, the rational coordination tailoring of surface-active sites on both photoanode and cathode should be a feasible strategy to achieve highly efficient PEC H_2_O oxidation and CO_2_ reduction.

## Experimental Section

### Preparation of Nanoporous BiVO_4_ Photoanodes

Nanoporous BiVO_4_ photoanodes were synthesized by the previous report [22]. 0.9701 g Bi(NO_3_)_3_·5H_2_O was added to 50 mL of 0.4 M KI solution; then, the pH was adjusted to 1.7 by HNO_3_ solution. Next, this solution was mixed with the 0.23 M quinhydrone ethanol solution (20 mL) and stirred vigorously for a few minutes to obtain the electrodeposited solution. In a typical three-electrode system, FTO, Ag/AgCl (3 M KCl), and a Pt foil act as working electrode, reference electrode, and counter electrode, respectively. Potentiostatic cathodic deposition is done at -0.1 V (vs. Ag/AgCl) for 3 min at room temperature to acquire the BiOI electrodes. And the 0.2 M VO(acac)_2_ was dissolved in DMSO (10 mL); subsequently, the 200 μL solution was coated on the BiOI electrode and heated under the air in a muffle furnace at 450 °C for 2 h with a ramping rate of 2 °C min^−1^. After calcination, the excess V_2_O_5_ on the electrodes can be removed by immersing into 1 M NaOH solution for 15 min. Finally, the electrodes were rinsed with deionized water and dried in air to obtain pure BiVO_4_ photoanodes.

### Preparation of BiVO_4_/NiFe-Ov Photoanodes

BiVO_4_/NiFeOOH (BiVO_4_/NiFe) photoanodes were prepared by a simple immersion method according our group’s previous work [24]. The BiVO_4_ photoanodes were immersed into the mixed solution of FeCl_3_·6H_2_O (10 mM, 7.5 mL) and NiCl_2_·6H_2_O (10 mM, 2.5 mL) for 15 min at room temperature. Then the pH was adjusted to 8 by adding 2 M NaOH solution and stirred gently for 5 min. Subsequently, after soaking for 45 min, the electrodes were rinsed with deionized water and blow-dried to obtain BiVO_4_/NiFe photoanodes. The BiVO_4_/NiFe photoanodes were treated by Ar plasma with a medium power (10.5 W) under 300 Pa for 2, or 5 min, noted as BiVO_4_/NiFe-Ov and BiVO_4_/NiFe-Ov (5 min), respectively.

### Preparation of ZIF-8 and NC

A solution of Zn(NO_3_)_2_·6H_2_O (1.67 g) dissolved in 42 mL of methanol was mixed with 2-methylimidazole methanol solution (1.84 g/21 mL) and stirred vigorously for 1 h, and then kept still for 24 h at room temperature. The white solid (ZIF-8) was collected by centrifugation, washed with methanol for three times, and dried at 60 °C under vacuum. The ZIF-8 samples were calcined at 1000 °C in a tube furnace under Ar flow for 2 h with the ramping rate of 2 °C min^−1^ to obtain nitrogen-doped carbon (NC).

### Preparation of CoPc-NC Cathodes

60 mg as-prepared NC was dispersed in 60 mL DMF with ultrasonication for 30 min. Then 12 mg CoPc was added to the suspension and followed by ultrasonication for 30 min and then stirred for 24 h at room temperature. The precipitate was centrifuged and washed by DMF and ethanol, followed by dried at 60 °C under vacuum to yield CoPc-NC.

5 mg catalyst and 170 μL of 5% nafion solution were added into 830 μL ethanol and sonicated for 1 h to obtained the well-dispersed ink. Then, the above ink of 100 μL was dropped onto a carbon cloth with area of 1 cm^2^.

### Photoelectrochemical Water Oxidation Measurements

The typical three-electrode system was used in the electrochemical workstation (CHI760D), in which the photoanode, the Ag/AgCl electrode, and the Pt electrode were used as the working electrode, reference electrode, and the counter electrode, respectively. 0.5 M K_3_BO_3_ solution (pH = 9.5) was used as electrolyte, and the illumination source was a simulated sunlight AM 1.5G (100 mW cm^−2^). The photocurrent vs. voltage (*J–V*) characteristics were determined by scanning potential from − 0.6 to 1.0 V (vs. Ag/AgCl) with a scan rate of 10 mV s^−1^, and the applied potentials could be converted to reversible hydrogen electrode (RHE):

$$E_{{{\text{RHE}}}} = E_{{\text{Ag/AgCl}}} + 0.0592pH + E_{{\text{Ag/AgCl}}}^{\theta }$$

where *E*_*RHE*_ is the potential vs. RHE, the value of *E*^*θ*^_Ag/AgCl_ is 0.197 V at 25 °C, and *E*_Ag/AgCl_ is the potential vs. Ag/AgCl.

The incident photo to current efficiency (IPCE) was determined by a full solar simulator (Newport, Model 9600, 300 W Xe arc lamp) and a motorized monochromator (Oriel Cornerstone 130 1/8 m) at 1.23 V_RHE_ in a 0.5 M K_3_BO_3_ electrolyte. The IPCE value was calculated using the equation:

$${\text{IPCE}} \left( \% \right) = \frac{{1240 \times I \left( {{\text{mA \, cm}}^{-2} } \right)}}{{P_{{{\text{light}}}} \left( {{\text{mW \, cm}}^{-2} } \right) \times \lambda \left( {{\text{nm}}} \right)}} \times 100$$

where *I* is the measured photocurrent density at specific wavelength, *λ* is the wavelength of incident light, and *P*_light_ is the power density of incident light at that wavelength (S130VC, THORLABS).

Electrochemical impedance spectroscopy (EIS) measurements were conducted on a frequency range of 0.01 to 10^5^ Hz under 0.75 *V*_RHE_. Cyclic voltammetry (CV) was measured for obtaining the electrochemical active surface area (ECAS) and carried out in 0.5 M K_3_BO_3_ electrolyte under 0.66 *V*_RHE_. The evolution of H_2_ and O_2_ was performed by an online gas analysis system (Labsolar 6A, Beijing Perfect Light Technology Co. Ltd.) equipped with a gas chromatograph (GC 7890A, Agilent).

### Photoelectrochemical CO_2_ Reduction Measurements

The photoelectrochemical CO_2_ reduction was conducted for an H cell with a proton exchange membrane (Nafion 115) separating the anode and cathode, and the electrolyte was 0.5 M K_3_BO_3_ and 0.5 M KHCO_3_ for anode and cathode, respectively. In this photoelectrochemical CO_2_ reduction system, the BiVO_4_/NiFe-Ov and Ag/AgCl were used as the working electrode and reference electrode in the anode cell, respectively, and the prepared CoPc-NC was used as the counter electrode in the cathode cell. And the working electrode was placed under simulated sunlight irradiation (100 mW cm^−2^) with different bias voltage, while the cathodic cell was placed in dark condition. Before the reaction, the cathode cell was saturated by bubbling with CO_2_ gas for 30 min. During the reaction, the gas composition of the cathode cell was analyzed by Shimadzu GC-2014C gas chromatography equipped with a Ni conversion furnace (TCD and FID, 5A molecular sieve and TDX-1 columns). The cycling test of CO_2_ reduction was performed at 1.23 V_RHE_ bias with a BiVO_4_/NiFe-Ov photoanode as the working electrode under the simulated solar irradiation and an CoPc-NC as the dark cathode. The electrolytes of the anode and cathode cells were composed of 0.5 M K_3_BO_3_ and CO_2_-saturated 0.5 M KHCO_3_ solution, respectively. After one hour of single cycle test, the gas in the cathode cell was detected by the GC, and the electrolyte solution was refilled for the next cycle test. The ^13^C labeled isotope tracer experiment was performed, and the products were analyzed by a gas chromatography-mass spectrometry (GC–MS, 7890A and 5975C, Agilent).

### Performance Evaluation of the Bias-Free PV-PEC CO_2_ Reduction System

In this PV-PEC CO_2_ reduction system, no additional voltage is applied and the cells include the polycrystal silicon solar cell, anode cell, cathode cell, and a proton exchange membrane (Nafion 115). The electrolyte was 0.5 M K_3_BO_3_ for anode and CO_2_-saturated 0.5 M KHCO_3_ for cathode. As the BiVO_4_/NiFe-Ov photoanode can only absorb light < 500 nm, we fully utilize light from the solar spectrum (> 500 nm) that is not available to the photoanode by means of a polycrystalline silicon solar panel. A long-pass filter (RG-510, Edmund Optics) is therefore added to the light outlet of another light source (AM 1.5 G) to provide light at > 500 nm. This long wavelength light irradiates a 54 × 54 mm^2^ polycrystalline solar cell with a maximum open circuit voltage of 2.0 V to provide the voltage to drive the entire system, where the total power irradiated on the solar cell is controlled at 2.6 mW cm^−2^ (calculated from the AM 1.5 G spectrum).

### Characterizations

The physical phase of the samples was determined by X-ray diffraction patterns (XRD, Smartlab-SE) with Cu Kα radiation in the 2θ range of 5°–80° at 50 kV and 50 mA. The morphology of samples was observed via Hitachi SU 8010 scanning electron microscopy (SEM) and transmission electron microscopy (TEM) with FEI Tecnai TF20 operated at an accelerating voltage of 200 kV. The elemental composition and chemical valence states were performed on X-ray photoelectron spectroscopy (XPS, Escalab 250Xi). UV–visible diffuse reflectance spectra were recorded by a Shimadzu UV 2550 spectrometer with the BaSO_4_ as the reference. Raman spectra (LabRAM HR Evolution) were obtained for probing the local structure by 532-nm laser. Transient absorption spectra (TA) and TA kinetics were conducted by an Edinburgh LP980 spectrophotometer with the 355-nm laser source. Temperature-programmed desorption profiles of CO_2_ (CO_2_-TPD) were obtained on a chemical adsorption instrument (Micromeritics, ChemiSorb 2720). Before the adsorbed CO_2_ molecules were desorbed under a purge of 25 mL min^−1^ of helium with a heating rate of 10 °C min^−1^, the samples were saturated with CO_2_ at 25 °C. XAFS spectrum of Co was collected at the BL14W beamline in Shanghai Synchrotron Radiation Facility (SSRF). The electron beam energy of the storage ring was 3.5 G eV with a maximum stored current of 250 mA. The hard X-ray was monochromatized with Si (111) double-crystal monochromator. The in situ FTIR tests of CO_2_ adsorption were performed on a Fourier transform infrared spectroscopy (VERTEX 70, Bruker). The gas flow was 20 mL min^−1^, and the data were collected at 3-min intervals after subtracting the initial background.

## Results and Discussion

### Characterization of BiVO_4_/NiFe-Ov Photoanode

**Fig. 1:**
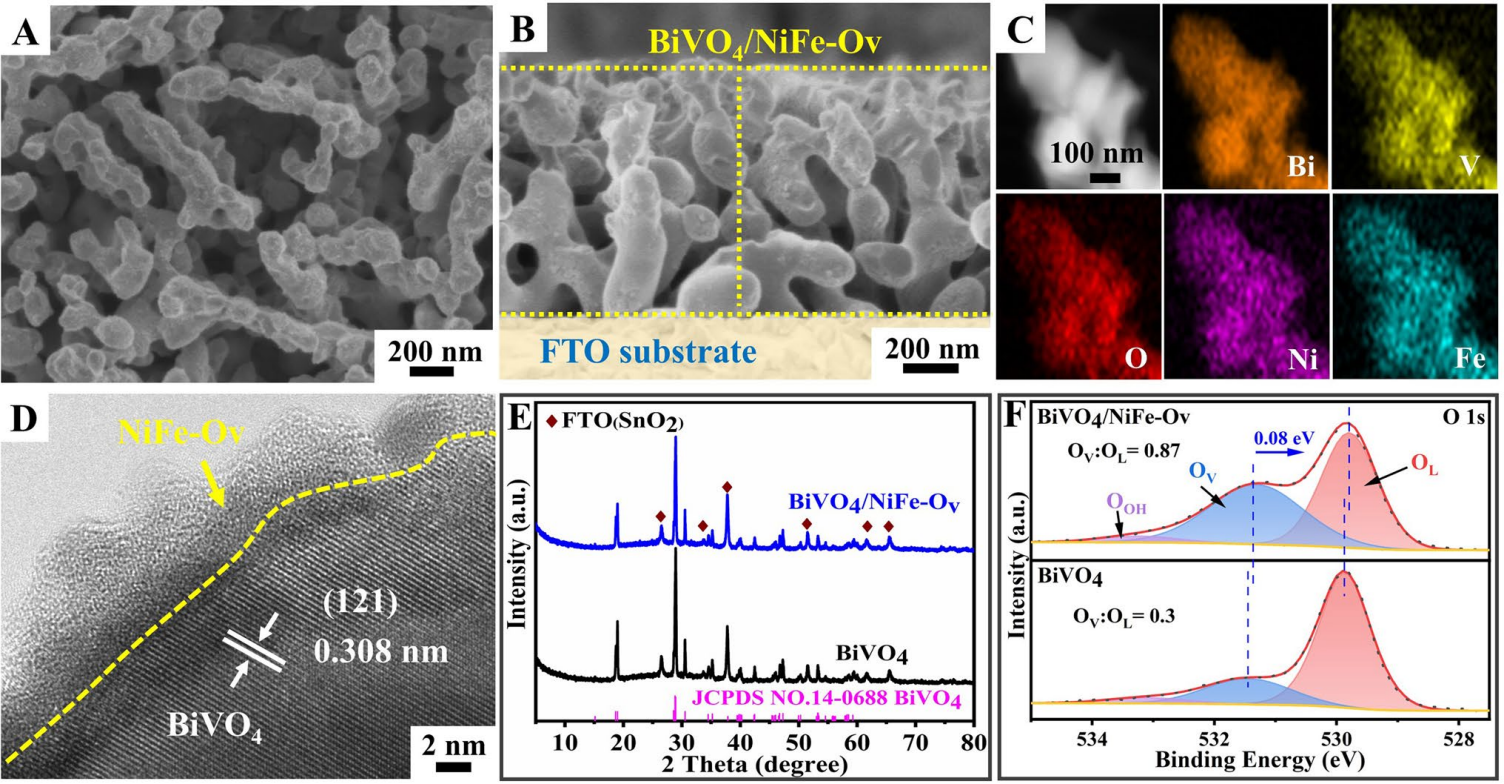
**A** Top-view SEM image, **B** cross-view SEM image, **C** EDS mapping images, and **D** HR-TEM image of the BiVO_4_/NiFe-Ov photoanode; **E** XRD patterns, and **F** XPS high-resolution O 1*s* spectra of the prepared BiVO_4_ and BiVO_4_/NiFe-Ov photoanodes

The BiVO_4_ photoanodes decorated with low oxygen-coordinated NiFe (NiFe-Ov) catalysts were fabricated by a facile Ar plasma treatment (Scheme S1). Firstly, the typical scanning electron microscopy (SEM) images of pristine BiVO_4_ photoanodes are shown in Fig. S1, revealing a worm-like nanoporous structure with the diameter of 200–300 nm as well as a relatively smooth surface. The high-resolution transmission electron microscopy (HR-TEM) image (Fig. S1D) clearly indicates that an evident lattice spacing of 0.293 nm could be corresponded to (040) plane of monoclinic BiVO_4_ phase. Noteworthy, after the decoration of NiFe-Ov catalysts, the smooth surfaces of BiVO_4_ photoanodes have transformed into a rough flocculent structure (Figs. 1A and S2). Figure 1B shows the cross-sectional SEM image of BiVO_4_/NiFe-Ov photoanodes, and the average thickness of photoanode films is about 1 μm. The HR-TEM images (Figs. 1D and S2D) clearly reveal that an amorphous nanolayer of NiFe-Ov catalysts with a thickness of ~ 5 nm uniformly covers the surface of BiVO_4_ nanocrystal. Furthermore, Figs. 1C and S3 present the energy dispersive spectroscopy (EDS) elemental mapping analysis, and the distribution of Fe element (~ 330 nm) is larger than that of Bi element (~ 320 nm), revealing the NiFe catalysts should be located on the BiVO_4_ surfaces. Figure 1E shows the XRD patterns of both pristine BiVO_4_ and BiVO_4_/NiFe-Ov photoanodes. Compared with the pristine BiVO_4_ samples, no evident change on the diffraction peaks could be observed, attributing to the amorphous structure and ultrathin thickness of NiFe-Ov catalysts. Moreover, both XPS high-resolution O 1*s* spectra and electron paramagnetic resonance spectroscopy (EPR) clearly represent that the oxygen vacancies have been significantly increased over BiVO_4_/NiFe-Ov photoanodes compared with the pristine BiVO_4_ (Figs. 1F and S16), confirming the formation of abundant oxygen vacancies on NiFe-Ov catalysts. Additionally, the O 1*s* peak of BiVO_4_/NiFe-Ov shifted to the low binding energy (~ 0.08 eV) region compared with pristine BiVO_4_, attributing to the electron enrichment induced by oxygen vacancies.

### Photoelectrochemical Properties of BiVO_4_/NiFe-Ov Photoanode

**Fig. 2:**
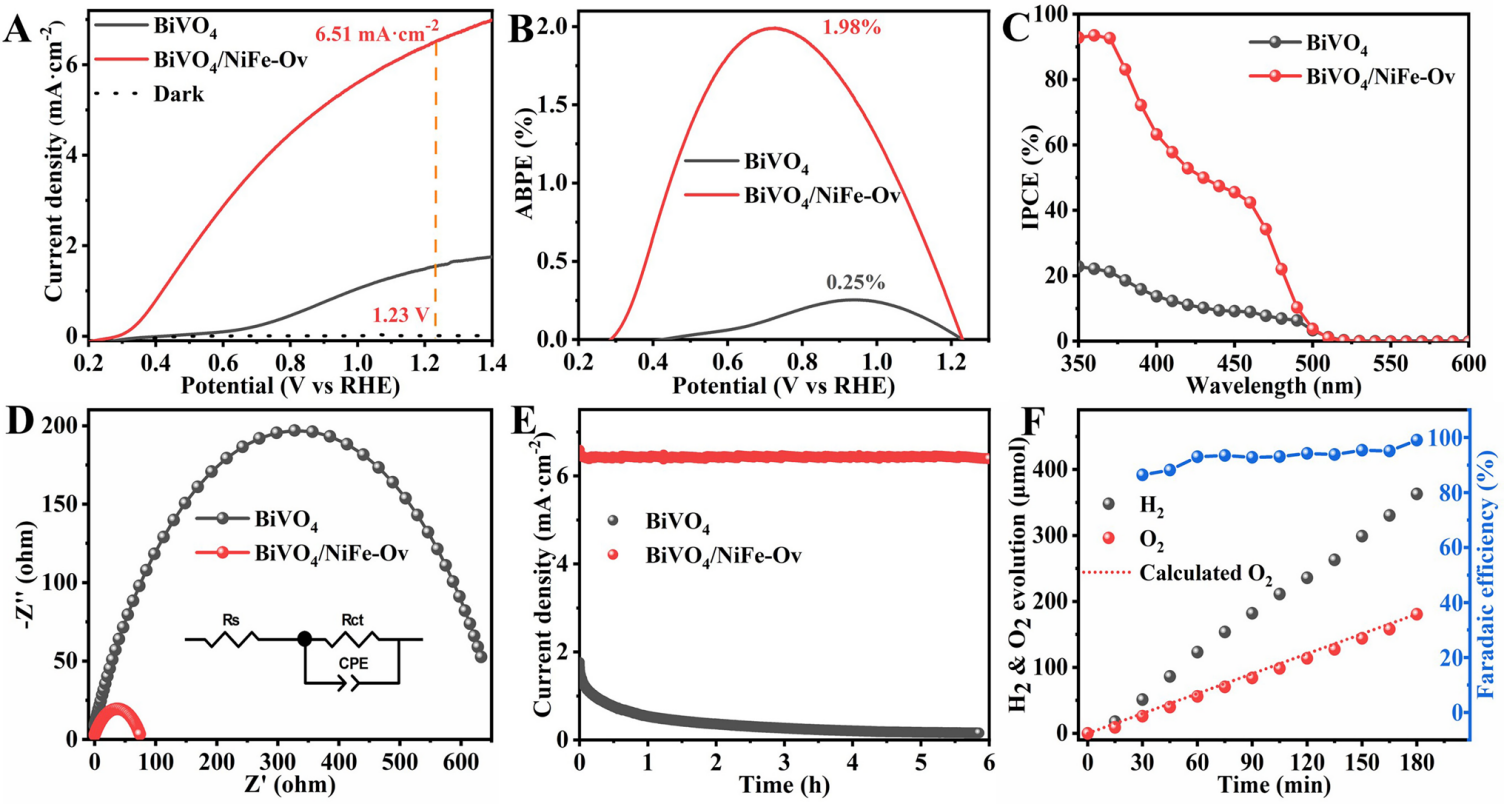
**A** LSV (scan rate of 10 mV s^−1^), **B** ABPE, **C** IPCE, **D** EIS plots at 0.75 V_RHE_ under illumination, and **E** PEC stability test at 1.23 V_RHE_ of BiVO_4_ and BiVO_4_/NiFe-Ov photoanodes; **F** the related H_2_ and O_2_ evolution amounts at 1.23 V_RHE_

The PEC water oxidation activities of pristine BiVO_4_ and BiVO_4_/NiFe-Ov photoanodes were performed in 0.5 M K_3_BO_3_ (pH = 9.5) electrolyte under AM 1.5G illumination (100 mW cm^−2^). As shown in Fig. 2A, the photocurrent density of BiVO_4_/NiFe-Ov photoanode could be remarkably increased up to 6.51 mA cm^−2^ at 1.23 V_RHE_ compared with the pristine BiVO_4_ of 1.55 mA cm^−2^, indicating the significant improvement in oxygen evolution at photoanode/electrolyte interfaces. The applied bias photon to current efficiencies (ABPE) were calculated and are shown in Fig. 2B, and the ABPE value of BiVO_4_/NiFe-Ov photoanode has been achieved up to 1.98% at 0.72 V_RHE_, which is much higher than that of pristine BiVO_4_ (0.25% at 0.94 V_RHE_). Additionally, Fig. 2C shows the incident photon to current conversion efficiency (IPCE) of pristine BiVO_4_ and BiVO_4_/NiFe-Ov photoanodes. Compared with the pristine BiVO_4_, a maximum value of 93.5% at the wavelength of 360 nm has been obtained on BiVO_4_/NiFe-Ov photoanode. Furthermore, the EIS has been performed to clarify the interfacial charge separation and transfer (Fig. 2D). According to the Nyquist plots and the fitting results (Table S1), the calculated resistance values of BiVO_4_/NiFe-Ov and pristine BiVO_4_ photoanodes were 76.1 and 665.1 Ω, respectively, revealing the preferable competence of NiFe-Ov catalysts for accelerating interface charge transfer.

Furthermore, the PEC water oxidation stability has been conducted at 1.23 V_RHE_ and is shown in Fig. 2E. It could be obviously found that after the decoration of NiFe-Ov catalyst, the photocurrent density of BiVO_4_ photoanodes could be well kept consistent during 6 h test process, indicating the constructive role of NiFe-Ov on restraining V^5+^ dissolution from BiVO_4_ lattices [43]. Figure S18 shows the chemical compositions and electronic structures of NiFe catalysts in the obtained BiVO_4_/NiFe-Ov photoanodes. It could be clearly seen that both Fe and Ni species in the OER catalyst layers exhibit two oxidation states of + 2 and + 3. More importantly, a high proportion of both Fe^2+^ and Ni^2+^ have been significantly improved on BiVO_4_/NiFe-Ov photoanodes, attributing to the low oxygen coordination and electron enrichment of NiFe-Ov catalysts. Accordingly, the photo-generated holes could be efficiently extracted from BiVO_4_ bulk by NiFe-Ov catalyst layer (Bi/V–O–Ni/Fe) for promoting charge separation and water oxidation performances (Scheme S3). Moreover, the hydrogen and oxygen amounts generated from PEC water splitting over BiVO_4_/NiFe-Ov photoanode were determined by a gas chromatography (GC). As shown in Fig. 2F, the production of H_2_ and O_2_ shows linear increase with prolonging reaction time. After 3 h light irradiation, the amounts of H_2_ and O_2_ products could be reached up to 362.9 (H_2_) and 180.8 μmol (O_2_), respectively, and an average faradaic efficiency of 93.2% has been acquired on BiVO_4_/NiFe-Ov photoanode, further confirming its prominent water oxidation capability. Thereby, the rational decrease in oxygen coordination of FeNi OER catalysts could significantly promote the PEC water oxidation performances.

### Characterization of CoPc-NC Cathode

**Fig. 3:**
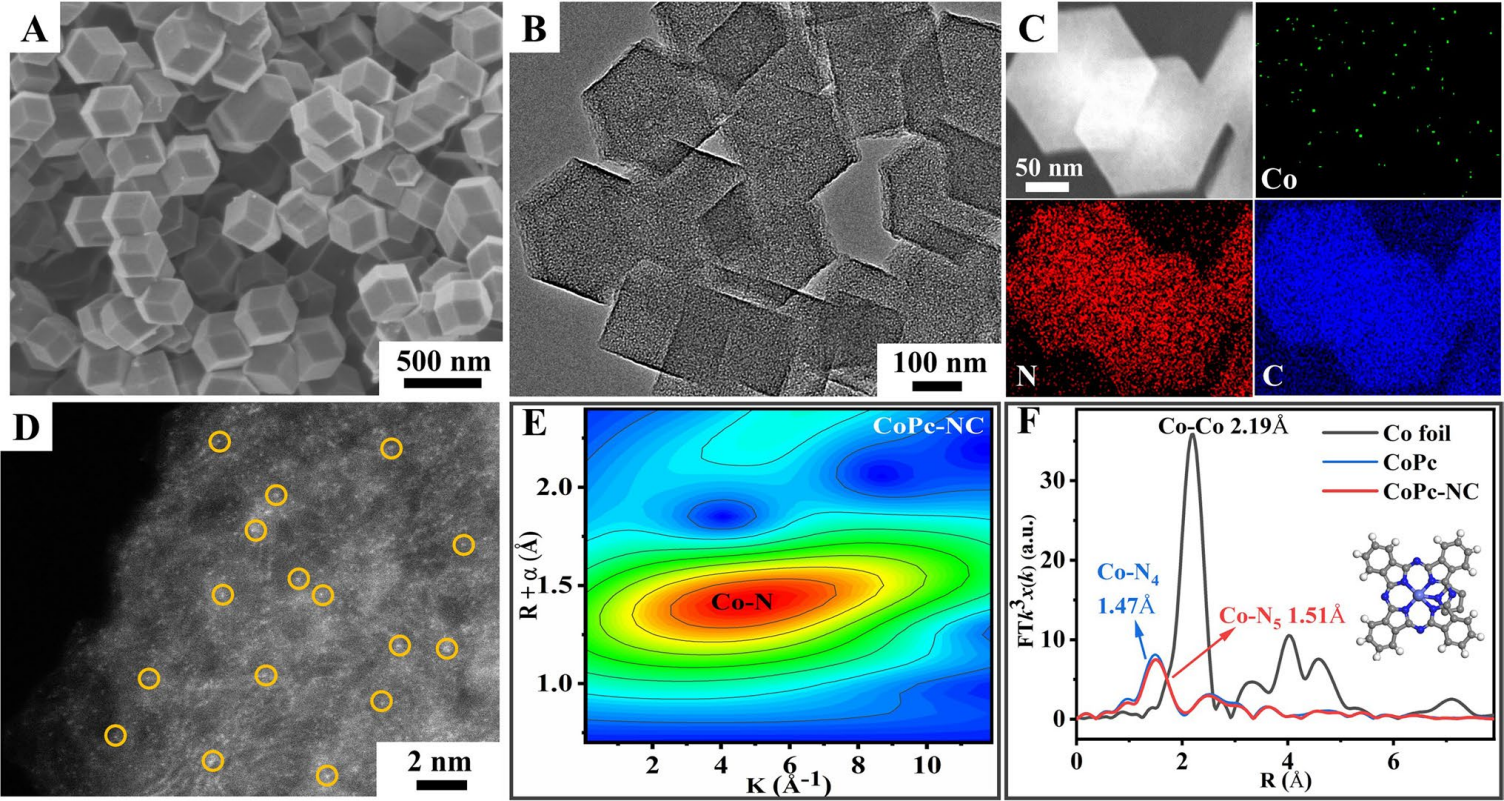
**A** SEM image, **B** TEM image, **C** EDS mapping image, **D** HAADF-STEM image, and **E** EXAFS wavelet transformed spectra of CoPc-NC; **F** Fourier transformation of EXAFS analysis of Co foil, CoPc, and CoPc-NC

To achieve efficient PEC CO_2_ reduction, a highly active photoanode must integrate with a highly selective cathode catalyst to ensure the CO_2_ adsorption and activation. Herein, single-atom CoPc anchoring on N-rich carbon substrates synthesized has been selected as the cathode catalyst to couple with the above BiVO_4_/NiFe-Ov photoanode. The SEM and TEM images clearly reveal that the obtained CoPc-NC catalysts possess the uniform rhombic dodecahedron morphology with an average size of ~ 250 nm (Figs. 3A, B and S20, S21), and no evident aggregation of CoPc clusters could be detected, which is agreement with the XRD and Raman results (Fig. S22). Moreover, the EDS elemental mapping images (Fig. 3C) reveal that Co, N, and C signals could be well detected in the whole sample regions, further confirming the uniform dispersion of CoPc on NC substrates. High-angle annular dark field-scanning transmission electron microscopy (HAADF-STEM) images (Fig. 3D) confirm that the Co single atoms are uniformly anchored on the N–C substrates. Furthermore, X-ray absorption structure (XAS) spectroscopy has also been applied to explore the chemical structures and coordination details. As shown in Fig. 3E, the EXAFS wavelet transformed (WT) spectra of CoPc-NC indicate that the maximum intensity of WT contour plots is located at ~ 4.3 Å^−1^, which corresponds to Co–N bonds instead of Co–Co bonds at ~ 7.2 Å^−1^ (Fig. S24), confirming the formation of Co single atoms on N-rich carbon substrates. More specifically, the Fourier transformation of EXAFS analysis (Fig. 3F) clearly reveals that the peak at ~ 1.47 and 1.51 Å could be assigned to Co–N bonds for CoPc and CoPc-NC, respectively. Furthermore, the Co K-edge XANES spectra (Fig. S25) indicate that the spectra of CoPc-NC could shift to higher energy compared with CoPc. The fitting results shown in Fig. S26 and Table S5 reveal that the coordination environment of Co single atom in CoPc-NC catalysts should be Co-N_5_, which could be further supported by XPS results shown in Fig. S27 and Table S7. As shown in Fig. S28, compared with pristine CoPc samples, the Co 2*p* peak of CoPc-NC catalysts shifted to the low binding energy region, indicating the efficient electron injection from NC substrates to single-atom Co sites [44, 45].

### Performance Evaluation of Photoelectrochemical CO_2_ Reduction

**Fig. 4:**
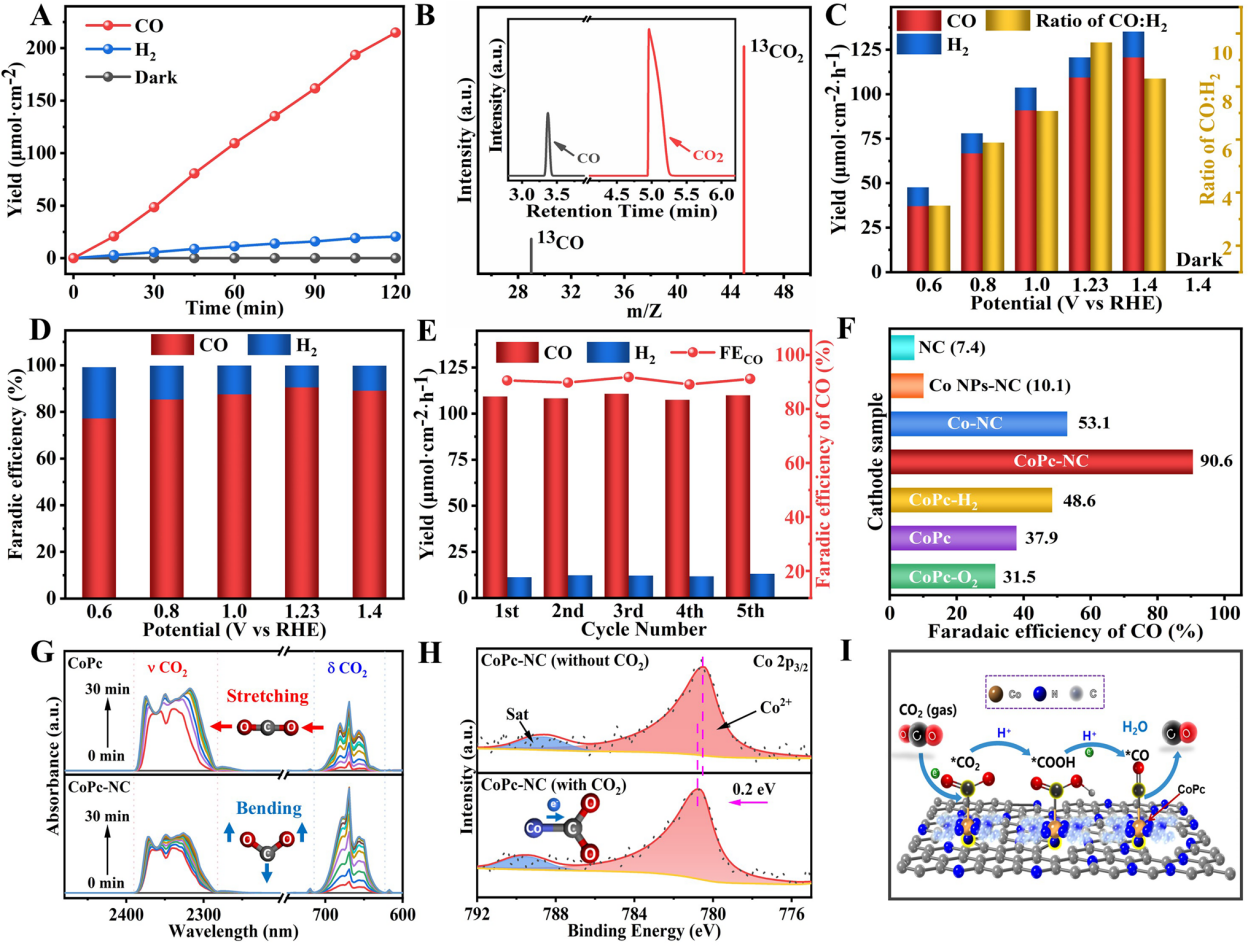
**A** CO and H_2_ evolution amount of BiVO_4_/NiFe-Ov‖CoPc-NC tandems at 1.23 V_RHE_; **B** GC–MS product analysis using ^13^CO_2_ as the carbon source; **C** CO/H_2_ yields and **D** total Faradaic efficiency of BiVO_4_/NiFe-Ov‖CoPc-NC tandems at different potential; **E** CO/H_2_ yields and Faradaic efficiency of CO during cycling tests at 1.23 V_RHE_; **F** Faradaic efficiency of CO by integrating BiVO_4_/NiFe-Ov photoanode with different cathodes; **G** In-situ FTIR spectra of CO_2_ adsorption for CoPc and CoPc-NC; **H** XPS high-resolution Co 2*p* spectra of CoPc-NC before and after CO_2_ adsorption; **I** The possible mechanism of BiVO_4_/NiFe-Ov‖CoPc-NC tandems for PEC CO_2_ reduction

The PEC CO_2_ reduction performances of CoPc-NC cathode integrated with BiVO_4_/NiFe-Ov photoanode have been carried out in the H-type cell with CO_2_ saturated 0.5 M KHCO_3_ as the electrolyte. As shown in Figs. 4A and S30, after 1 h irradiation on the BiVO_4_/NiFe-Ov photoanode at 1.23 V_RHE_, the amounts of CO and H_2_ production from CoPc-NC cathode chamber increased up to 109.4 and 11.3 μmol cm^−2^, respectively. Nevertheless, when light irradiation was removed from BiVO_4_/NiFe-Ov photoanode, no any gas production could be detected on CoPc-NC cathode, indicating that the PEC water oxidation on photoanodes for releasing electron/proton should be crucial to initiate CO_2_ reduction reaction into CO on the cathodes. Furthermore, the isotopic labeling experiment has been performed by using ^13^CO_2_ as a carbon source and is shown in Fig. 4B. The ^13^CO peak at *m*/*z* = 29 has been obviously detected in this PEC CO_2_ reduction system, confirming that CO could be produced from CO_2_ rather than other impurities. It is noteworthy that the ratio of CO: H_2_ (3.5–9.7) could be conveniently controlled by changing the applied voltage (Fig. 4C), and all the Faradic efficiencies of CO are higher than 85% when applied potential is greater than 0.8 V_RHE_ (Fig. 4D). Additionally, the stability of this PEC CO_2_ reduction system has been evaluated by the cyclic experiments. As shown in Fig. 4E, the production amount and faradaic efficiency of CO (FE_CO_) could be kept constant during the five cycles, confirming its outstanding durability for CO_2_ reduction. To further validate the high activity and selectivity of CoPc-NC cathode for PEC CO_2_ reduction, the NC, Co NPs-NC, Co-NC, and CoPc cathode catalysts have also been coupled with BiVO_4_/NiFe-Ov photoanodes. As shown in Figs. 4F and S32, the CO yields on the above cathodes were all much lower than that of CoPc-NC. In particular, the CO faradaic efficiency of CoPc has been only reached to 37.9%, indicating that the excellent activity and selectivity of CoPc-NC could be attributed to the coupling of CoPc with NC substrates.

To further clarify the details of CO_2_ adsorption and activation on CoPc-NC cathode surface, CO_2_ temperature-programmed desorption (CO_2_-TPD) has been conducted and is shown in Fig. S34. Accordingly, a broad CO_2_ desorption peak (~ 306 °C) has been detected on CoPc, while the peak of CoPc-NC mainly located at ~ 389 °C, indicating that the introduction of CoPc on NC substrate could markedly increase the chemisorption of CO_2_ molecules. Furthermore, in situ Fourier transform infrared reflection (FTIR) spectra shown in Fig. 4G reveal that the bending vibration (~ 667 cm^−1^) peaks of CO_2_ on CoPc-NC catalyst have been significantly enhanced compared with CoPc sample, indicating that CoPc-NC catalyst should be beneficial to the activation of CO_2_ due to the powerful O–C–O bending vibration [46, 47]. Therefore, the CO_2_-TPD and in situ FTIR results confirm that CoPc-NC possesses a much stronger CO_2_ adsorption and activation capability than pristine CoPc. Furthermore, quasi-in situ XPS spectra have been performed to explore the charge transfer processes during CO_2_ adsorption and activation process. Figure S35 shows that the O 1*s* spectra of CoPc and CoPc-NC before and after CO_2_ adsorption, and the O 1*s* peak of CO_2_ adsorption could be divided into two oxygen species of C=O (~ 533.2 eV) and C–O (~ 531.6 eV). Note that the CoPc-NC exhibits a larger proportion of C-O peak than CoPc, indicating the higher CO_2_ adsorption and activation capability for CoPc-NC catalyst. As shown in Fig. 4H, the Co 2*p* peaks could shift to high binding energy (BE) direction (~ 0.2 eV) after CO_2_ adsorption, indicating electron transfer from single-atom Co sites to CO_2_ molecules. Based on the above analysis, a possible mechanism of PEC CO_2_ reduction over CoPc-NC cathode catalysts coupled with BiVO_4_/NiFe-Ov photoanode has been proposed (Fig. 4I). Under light irradiation, the photo-generated holes have migrated from bulk BiVO_4_ to NiFe surfaces for water oxidation, while the CO_2_ molecules adsorbed Co single atom have been interacted with the protons and electrons from OER to form *COOH and *CO; subsequently, the CO molecules desorbed from CoPc-NC surfaces.

### Performance Evaluation of Bias-Free PV-PEC CO_2_ Reduction

**Fig. 5:**
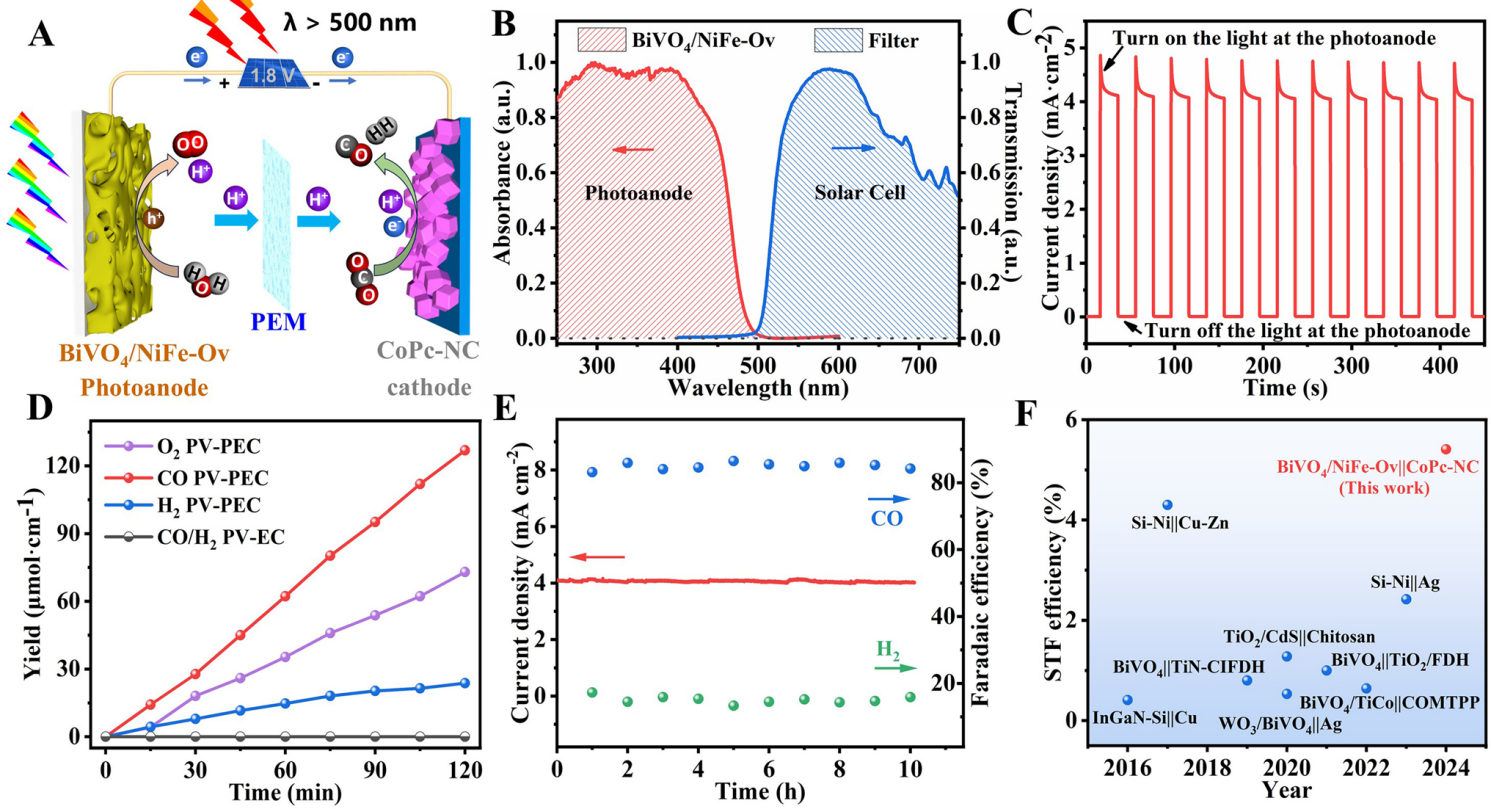
**A** Schematic diagram of BiVO_4_/NiFe-Ov‖CoPc-NC PV-PEC CO_2_ reduction system; **B** absorption and transmission spectra of BiVO_4_/NiFe-Ov photoanode and cutoff filter for solar cells, respectively; **C** chopped irradiation *i-t* curves, **D** O_2_, CO and H_2_ evolution amounts, **E** stability test of BiVO_4_/NiFe-Ov‖CoPc-NC PV-PEC CO_2_ reduction system; **F** comparisons of solar-to-fuel conversion efficiency for PEC-CO_2_ reduction

Note that BiVO_4_-based photoanodes could only absorb the sunlight spectra with a wavelength less than 500 nm, and a commercial polycrystal silicon solar cell for absorbing the residual sunlight (> 500 nm) has been integrated with above PEC device to construct an overall solar-driven CO_2_ reduction device (Fig. 5A, B). Under AM 1.5G illumination (100 mW cm^−2^), the photocurrent density of could be reached up to 4.1 mA cm^−2^ (Fig. 5C). However, when light irradiation on BiVO_4_ photoanode has been removed, the photocurrent density was rapidly dropped to near zero, and no any gas product could be detected in this PV-PEC reaction system (Fig. 5D), confirming that the PEC CO_2_ reduction should be driven by BiVO_4_/NiFe-Ov photoanode instead of solar cell. Additionally, the amounts of O_2_, CO and H_2_ products could reach up to 35.3, 62.2, and 14.7 μmol cm^−2^ after 1 h irradiation, respectively, and a stoichiometric ratio of ~ 2:1 (CO + H_2_/O_2_) has been obtained. As shown in Fig. 5E, the faradaic efficiency of CO and H_2_ could keep constant during the 10 h irradiation, suggesting the excellent stability of the PV-PEC system. The calculated the solar energy to CO and H_2_ conversion efficiency was 4.44% and 0.97%, respectively, corresponding to the solar-to-fuel (STF) conversion efficiency of 5.41%. Additionally, Fig. 5F and Table S11 show the STF conversion efficiency comparisons for the photoanode-driven PEC-CO_2_ reduction reported in recent years, and a recorded solar conversion efficiency has been achieved in this PV-PEC CO_2_ reduction system.

## Conclusions

In summary, we demonstrated the rational tailoring of the coordination environment of surface-active sites on both photoanode and cathodes, simultaneously achieving the highly active oxygen evolution and highly selective CO_2_ reduction. More specifically, one of the highest OER activities of 6.51 mA cm^−2^ (1.23 V_RHE_, AM 1.5G) has been obtained over BiVO_4_ photoanodes decorated with low-coordination ultrathin FeNi catalysts. Their integration with single-atom cobalt (II) phthalocyanine with Co-N_5_ coordination achieves a record production rate of 109.4 μmol mg^−1^ h^−1^ for CO production with a faradaic efficiency of 90.6%. Furthermore, a photovoltaic integrated this PEC system to construct an unbiased solar-driven CO_2_ reduction device for making full use of solar light, which could acquire a solar-to-fuel conversion efficiencies of 5.41%, accompanying with outstanding stability. Accordingly, developing highly efficient photoanode and integrating with highly selective CO_2_ reduction should be a promising strategy for solar-driven fuels production.

## Supplementary Information

Below is the link to the electronic supplementary material.

Supplementary file1 (DOCX 16765 KB)
