# Supplementary material for: Highly Active Oxygen Evolution Integrating with Highly Selective CO2-to-CO Reduction
Source: Nanomicro Lett. 2025 Mar 13;17:184. doi: 10.1007/s40820-025-01688-2 (PMC11904044; doi:10.1007/s40820-025-01688-2)
Supplement: Supplementary file 1 — Supplementary file1 (DOCX 16765 KB) [file 40820_2025_1688_MOESM1_ESM.docx]

Supporting Information for

**Highly Active Oxygen Evolution Integrating with Highly Selective CO_2_-to-CO Reduction**

Chaowei Wang^1,2^, Laihong Geng^3^, and Yingpu Bi^1,^*

^1^State Key Laboratory for Oxo Synthesis and Selective Oxidation, National Engineering Research Center for Fine Petrochemical Intermediates, Lanzhou Institute of Chemical Physics, Chinese Academy of Sciences, Lanzhou 730000, P. R. China

^2^University of Chinese Academy of Sciences, Beijing 100049, P. R. China

^3^Gansu Research Institute of Chemical Industry Co., Ltd, Lanzhou 730000, P. R. China

*Corresponding author. E-mail: [yingpubi@licp.cas.cn](mailto:yingpubi@licp.cas.cn) (Yingpu Bi)

**S1 Experimental Section**

**S1.1 Materials**

Bismuth nitrate pentahydrate (Bi(NO_3_)_3_·5H_2_O, 99%), potassium iodide (KI, 99%), quinhydrone, vanadium acetylacetone oxygen (VO(acac)_2_), nitric acid (HNO_3_), ethanol, iron chloride hexahydrate (FeCl_3_·6H_2_O, 99%), nickel chloride hexahydrate (NiCl_2_·6H_2_O, 98%), sodium hydroxide (NaOH, 96%), sodium sulfite (Na_2_SO_3_), boric acid (H_3_BO_3_), potassium hydroxide (KOH, 99%), zinc nitrate (Zn(NO_3_)_2_·6H_2_O, 99%), cobaltous nitrate hexahydrate (Co(NO_3_)_2_·6H_2_O, 99%), potassium bicarbonate (KHCO_3_, 99.5%) were purchased from Sinopharm Chemical Reagent Co., Ltd. 2-Methylimidazole (C_4_H_6_N_2_) and Cobalt phthalocyanine were bought from Shanghai Aladdin Biochemical Technology Co., Ltd. Deionized water (18.25 MΩ) was used in all reactions. All reagents were analytical grade and used without further purification. Fluorine doped Tin Oxide coated glasses (FTO) as substrates were obtained from Zhuhai Kaivo Electronic Components Co., Ltd. China. FTO was washed by ultrasonic cleaning with acetone, ethanol, isopropanol and deionized water, successively before use.

**S1.2 Preparation of BiVO_4_-Ov and BiVO_4_-Ov/NiFe photoanodes**

The BiVO_4_ photoanodes were treated by Ar plasma with a medium power (10.5 W) under 300 Pa for 2 min to obtain BiVO_4_-Ov. BiVO_4_-Ov/NiFe photoanodes were prepared by the above impregnation method excepted for BiVO_4_ was replaced with BiVO_4_-Ov.

**S1.3 Preparation of Co NPs-NC**

4 mmol Co(NO_3_)_2_·6H_2_O was dissolved in 40 mL deionized water, then 8 mmol NaBH_4_ was added into the solution and stirred for 12 h. Co NPs were obtained by centrifugation, washed with deionized and dried at 60 ℃ under vacuum. The 60 mg as-prepared NC was dispersed in 60 mL deionized water with ultrasonication for 30 min. Then 12 mg Co NPs were added to the suspension and followed by ultrasonication for 30 min, then stirred for 24 h at room temperature. The precipitate was centrifuged and washed by deionized water, followed by dried at 60 ℃ under vacuum to yield Co NPs-NC.

**S1.4 Preparation of Co-NC**

A solution of Zn(NO_3_)_2_·6H_2_O (1.67 g) and Co(NO_3_)_2_·6H_2_O (0.041 g) dissolved in 42 mL of methanol was mixed with 2-methylimidazole methanol solution (1.84 g/21 mL) and stirred vigorously for 1 h, then kept still for 24 h at room temperature. The solid (Co-MOF) was collected by centrifugation, washed with methanol for three times and dried at 60 ℃ under vacuum. The Co-MOF samples were calcined at 1000 ℃ in a tube furnace under Ar flow for 2 h with the ramping rate of 2 ℃/min to obtain Co-NC.

**S1.5 Performance evaluation of PEC CO_2_ reduction with two-electrode system**

In a typical artificial photosynthetic system, the cells consisting of the anode cell, cathode cell, and a proton exchange membrane (Nafion 115). The electrolyte was 0.5 M K_3_BO_3_ for anode and CO_2_-saturated 0.5M KHCO_3_ for cathode. And the prepared BiVO_4_/NiFe-Ov and CoPc-NC were used as the working electrode (photoanode) and counter electrode (cathode), respectively. In addition, the BiVO_4_/NiFe-Ov photoanode was connected with a working electrode, and the CoPc-NC cathode was connected with a counter electrode and reference electrode. And the working electrode was placed under simulated sunlight irradiation (100 mW/cm^2^) with different cell voltage, while the cathodic cell was placed in dark condition. Before the reaction, the cathode cell was saturated by bubbling with CO_2_ gas for 30 min. During the reaction, the gas composition of the cathode cell was analyzed by Shimadzu GC-2014C gas chromatography.

**S1.6 Faradaic efficiency**

$$FE \left( \% \right)=\frac{m\times n\times F}{I\times t}\times100$$

*FE* is the Faradaic efficiency; *m* is the mole amount of the product, mol; *n* is the number of electrons transferred in the reaction; *I* is the current density in the system, A; *t* is the reaction time, s; *F* is the Faradaic constant, 96485.3 C/mol.

**S1.7 Solar energy conversion efficiency**

The total reaction equation of the artificial photosynthetic cells:

$$xCO_{2} \left( g \right)+yH_{2}O \left( l \right)=xCO \left( g \right)+yH_{2} \left( g \right)+ \frac{x+y}{2}O_{2} (g)$$

The Gibbs free energy of the whole reaction:

$$\Delta_{r}G^{\theta}=x\Delta_{f}G_{m}^{\theta}\left( CO\left( g \right) \right)+y\Delta_{f}G_{m}^{\theta}\left( H_{2}\left( g \right) \right)+\frac{x+y}{2}\Delta_{f}G_{m}^{\theta}\left( O_{2}\left( g \right) \right)-x\Delta_{f}G_{m}^{\theta}\left( CO_{2}\left( g \right) \right)-y\Delta_{f}G_{m}^{\theta}\left( H_{2}O\left( l \right) \right)=257.2x+237.1y$$

Solar-to-CO conversion efficiency (STC):

$$STC \left( \% \right)= \frac{257.2x}{P_{Irradiate}\times t_{Irradiate}} \times100=\frac{257.2\times62.2}{100\times3600}\times100=4.44\%$$

Solar-to-H_2_ conversion efficiency (STH):

$$STH \left( \% \right)= \frac{237.1y}{P_{Irradiate}\times t_{Irradiate}} \times100=\frac{237.1\times14.7}{100\times3600}\times100=0.97\%$$

*P_Irradiate_* is the total power of the irradiation light, mW. *t_Irradiate_* is the time of irradiation, s. *x* and *y* are the production of CO and H_2_, respectively, μmol.

Solar-to-fuel conversion efficiency (STF):

$$STF (\%)=STC+STH=4.44\%+0.97\%=5.41\%$$

**S2 Supplementary Figures and Discussions**


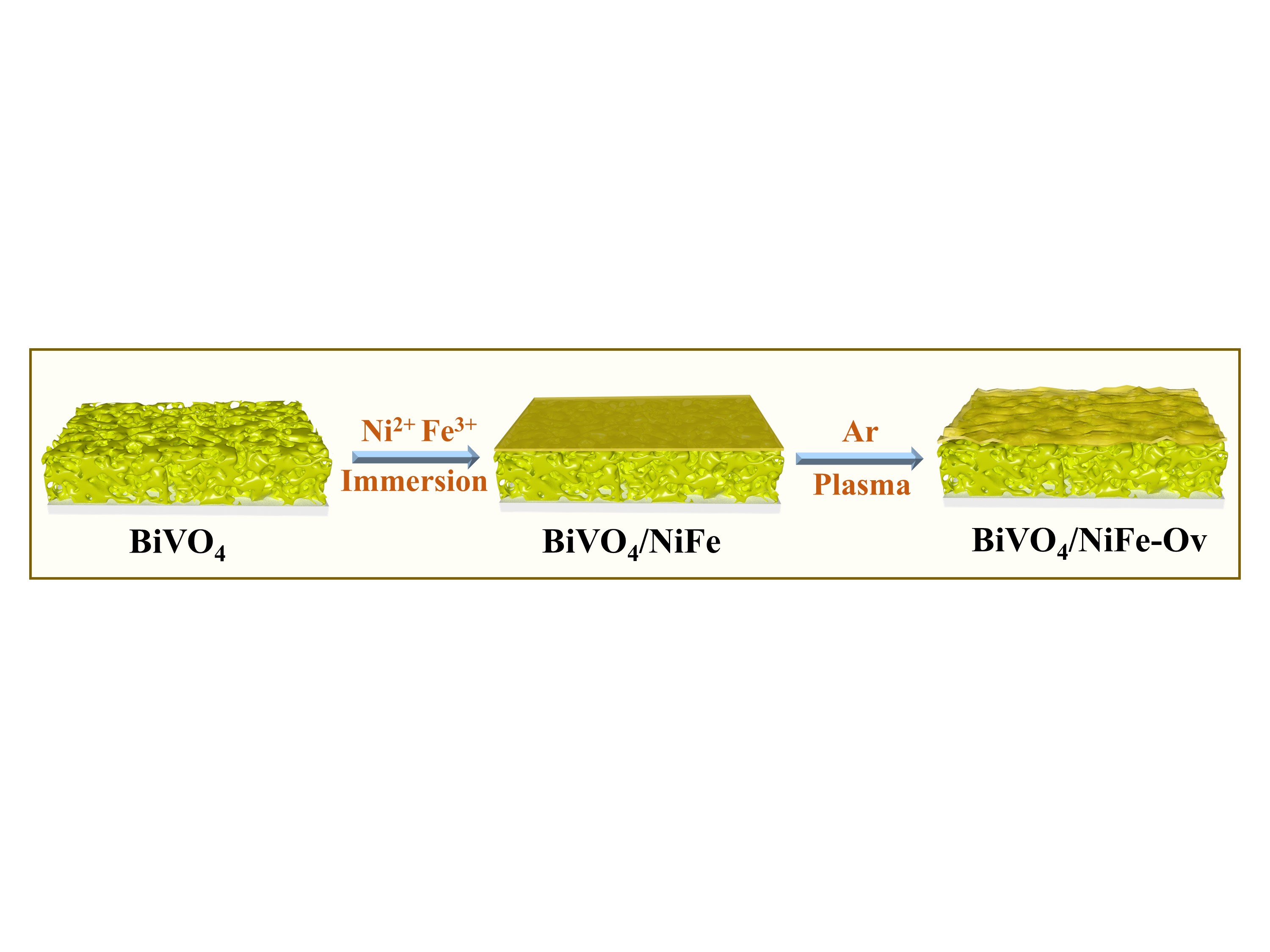


**Scheme S1** Diagram of the preparation process of BiVO_4_/NiFe-Ov photoanodes


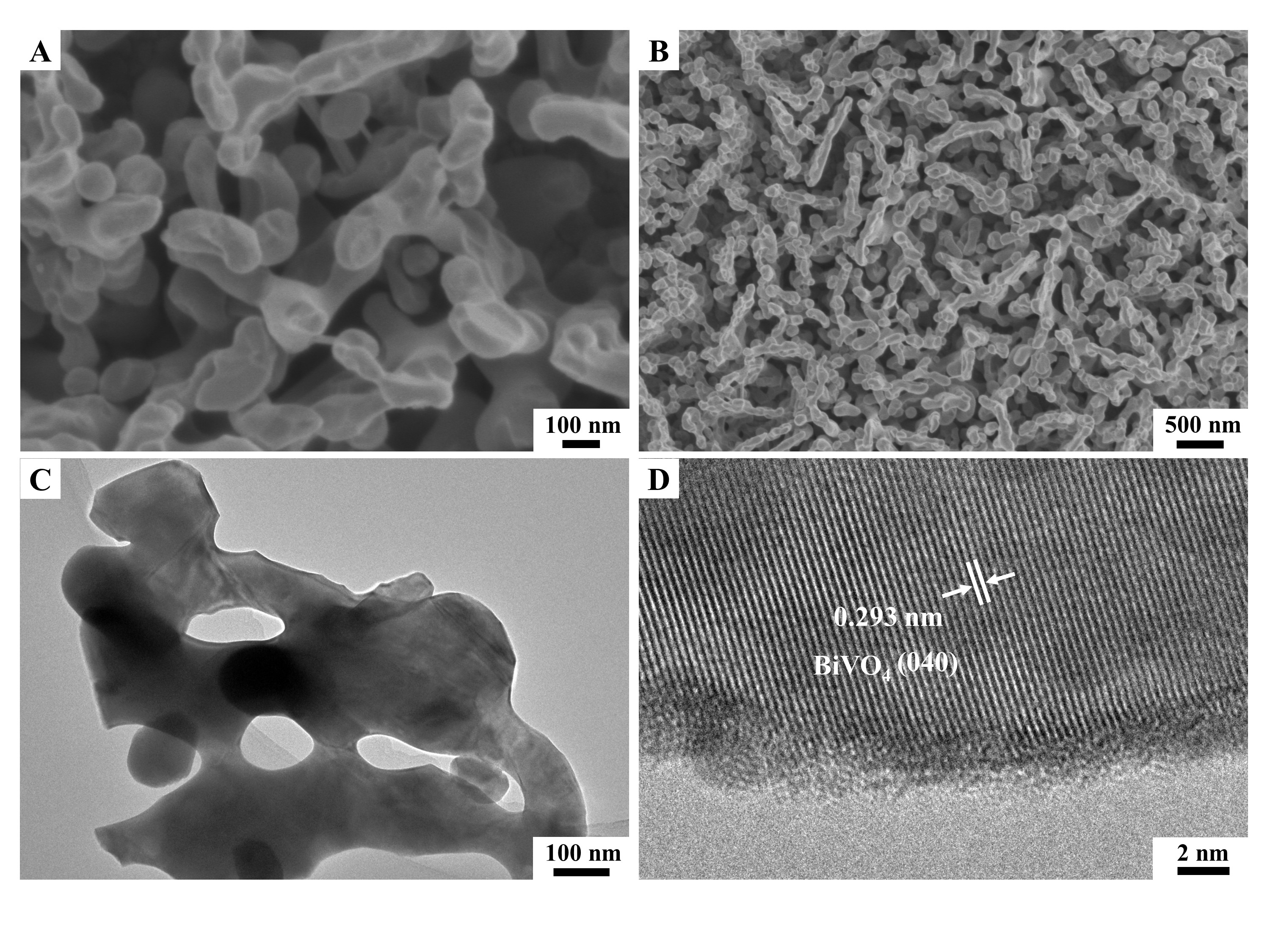


**Fig. S1** (**A, B**) SEM images of and (**C, D**)TEM images of BiVO_4_ photoanodes


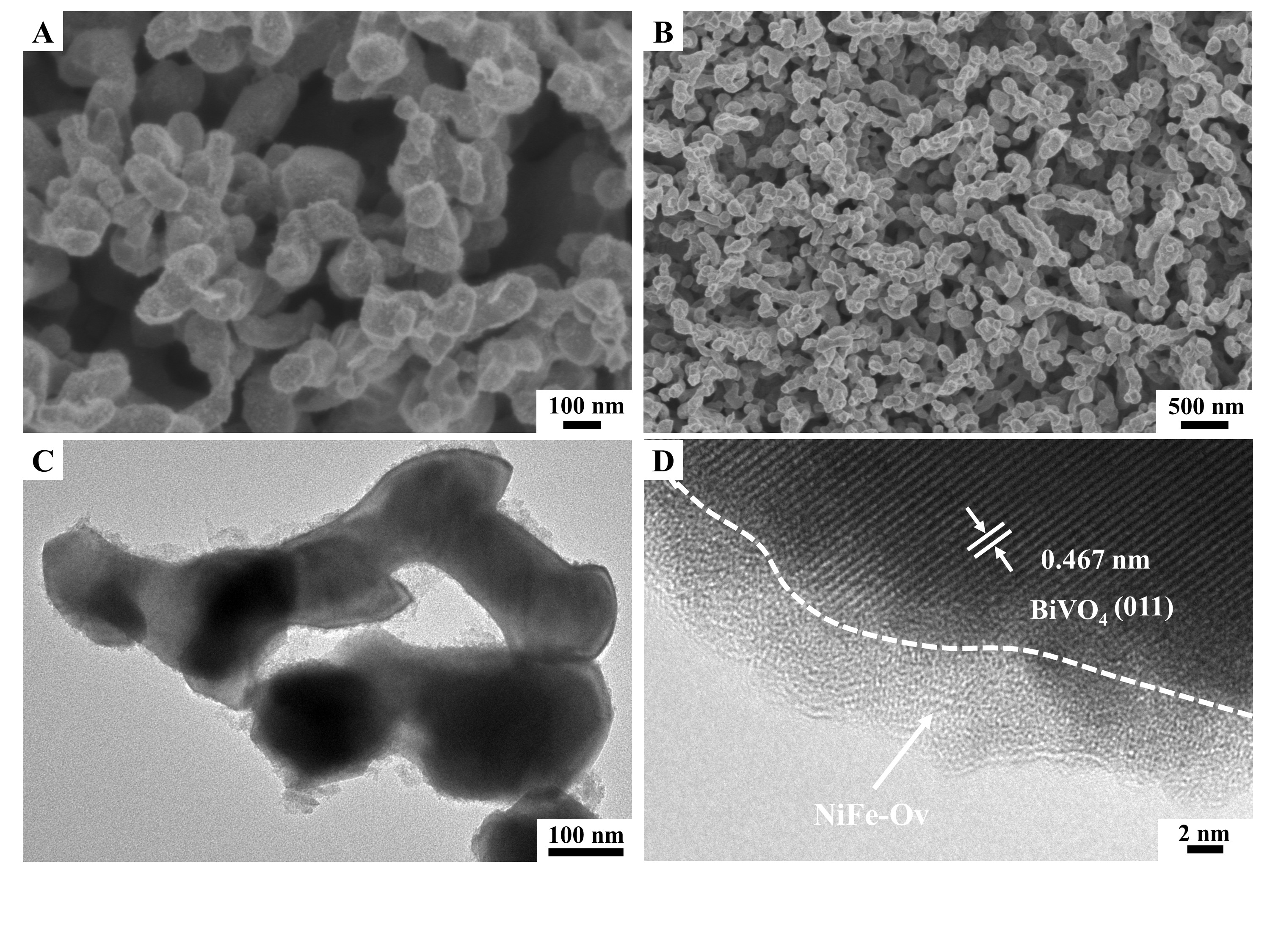


**Fig. S2** (**A, B**) SEM images of and (**C, D**)TEM images of BiVO_4_/NiFe-Ov photoanodes


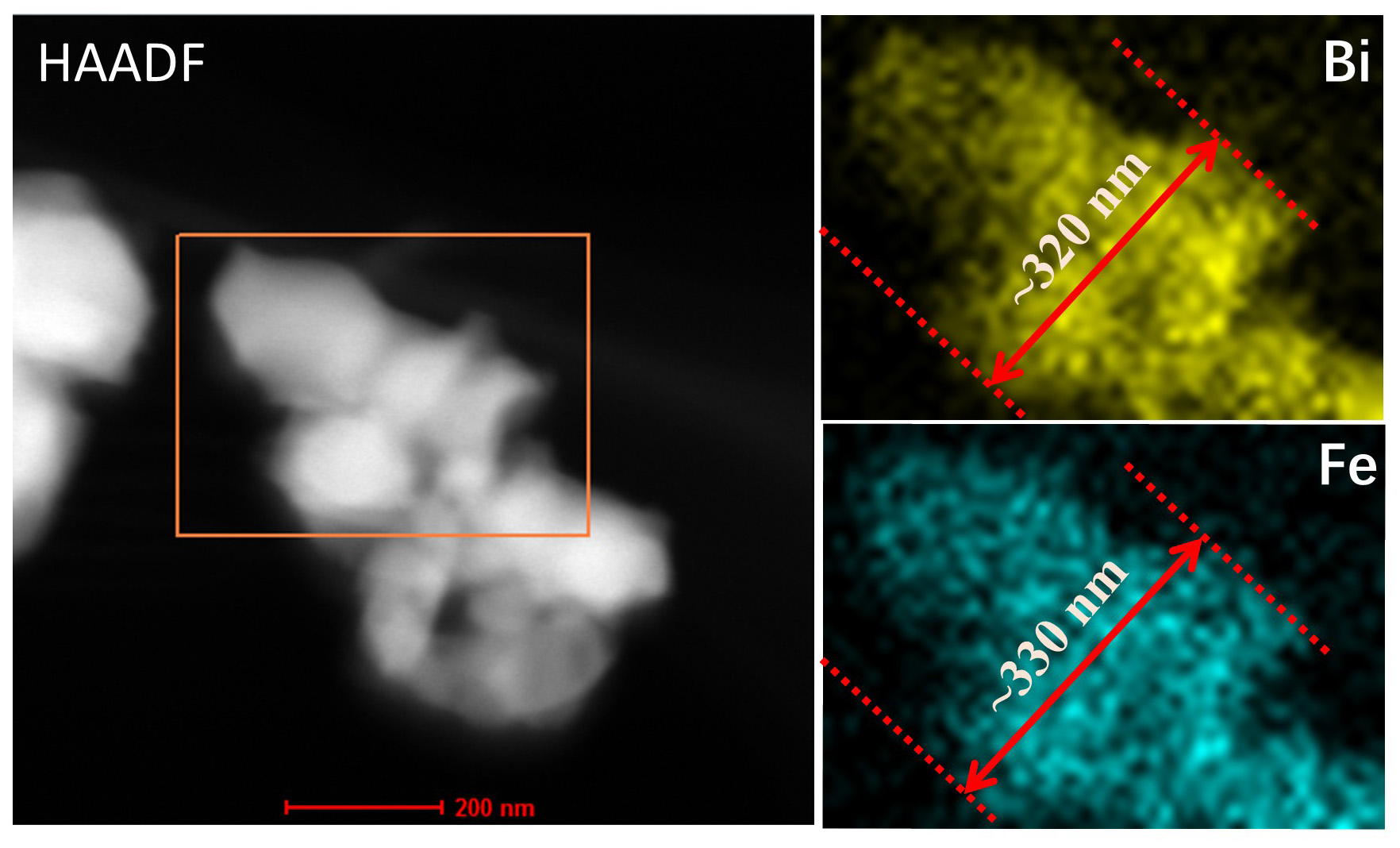


**Fig. S3** EDS mapping analysis of BiVO_4_/NiFe-Ov photoanodes


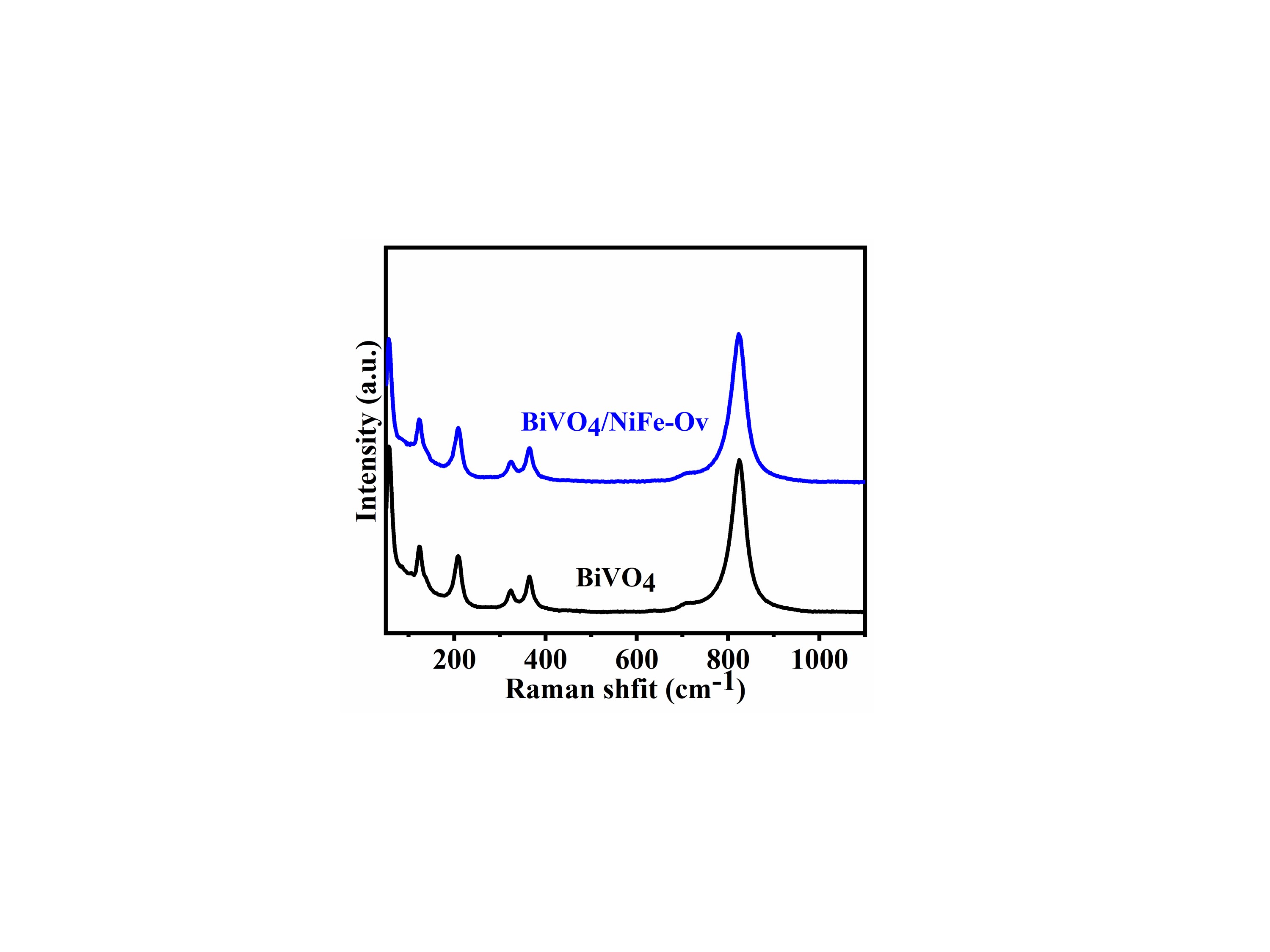


**Fig. S4** Raman spectra of BiVO_4_ and BiVO_4_/NiFe-Ov photoanodes

**Additional discussion**

Raman spectra was performed to explore the local structures under the green (532 nm) laser. It was found that the typical vibrational bands of BiVO_4_ located at 210, 324, 366, and 826 cm^-1^ were observed for BiVO_4_, which could be assigned to the vibration of VO_4_^3-^ (324 and 366 cm^-1^) and V-O (826 cm^-1^) [S1], and no bands of NiFeOOH was observed for BiVO_4_/NiFe-Ov. This result confirms the amorphous structure and ultrathin thickness of NiFe-Ov.


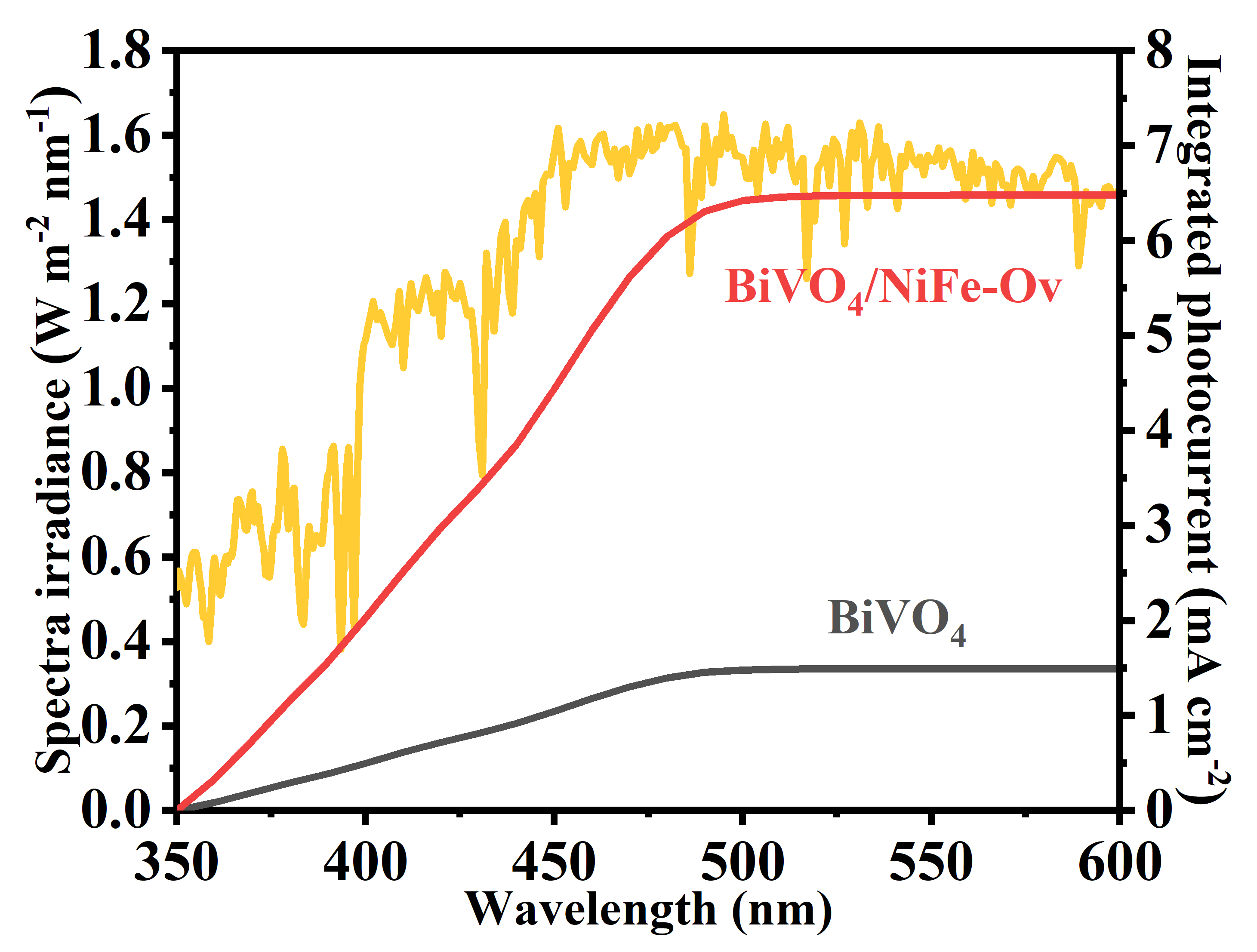


**Fig. S5** The calculated photocurrent density curves by integrating IPCE curves with the standard AM1.5 G spectrum

**Additional discussion**

The estimated photocurrent densities (J_c_) were calculated by integrating the IPCE values with the standard solar spectrum (ASTMG-173-03) using the following equation:

$$J_{c}=\int_{350}^{600} \frac{\lambda\times IPCE\left( \lambda\right)\times E\left( \lambda\right)}{1240}d(\lambda)$$

Specifically, λ and E(λ) represent the light wavelength (nm) and the corresponding solar power density (mW cm^-2^ nm^-1^), respectively. According to the above equation, the calculated photocurrent densities for BiVO_4_ and BiVO_4_/NiFe-Ov photoanodes were 1.49 and 6.48 mA cm^-2^ at 1.23 V_RHE_, respectively, which are both close to the measured values (1.55 and 6.51 mA cm^-2^ for BiVO_4_ and BiVO_4_/NiFe-Ov, Fig. 2a).


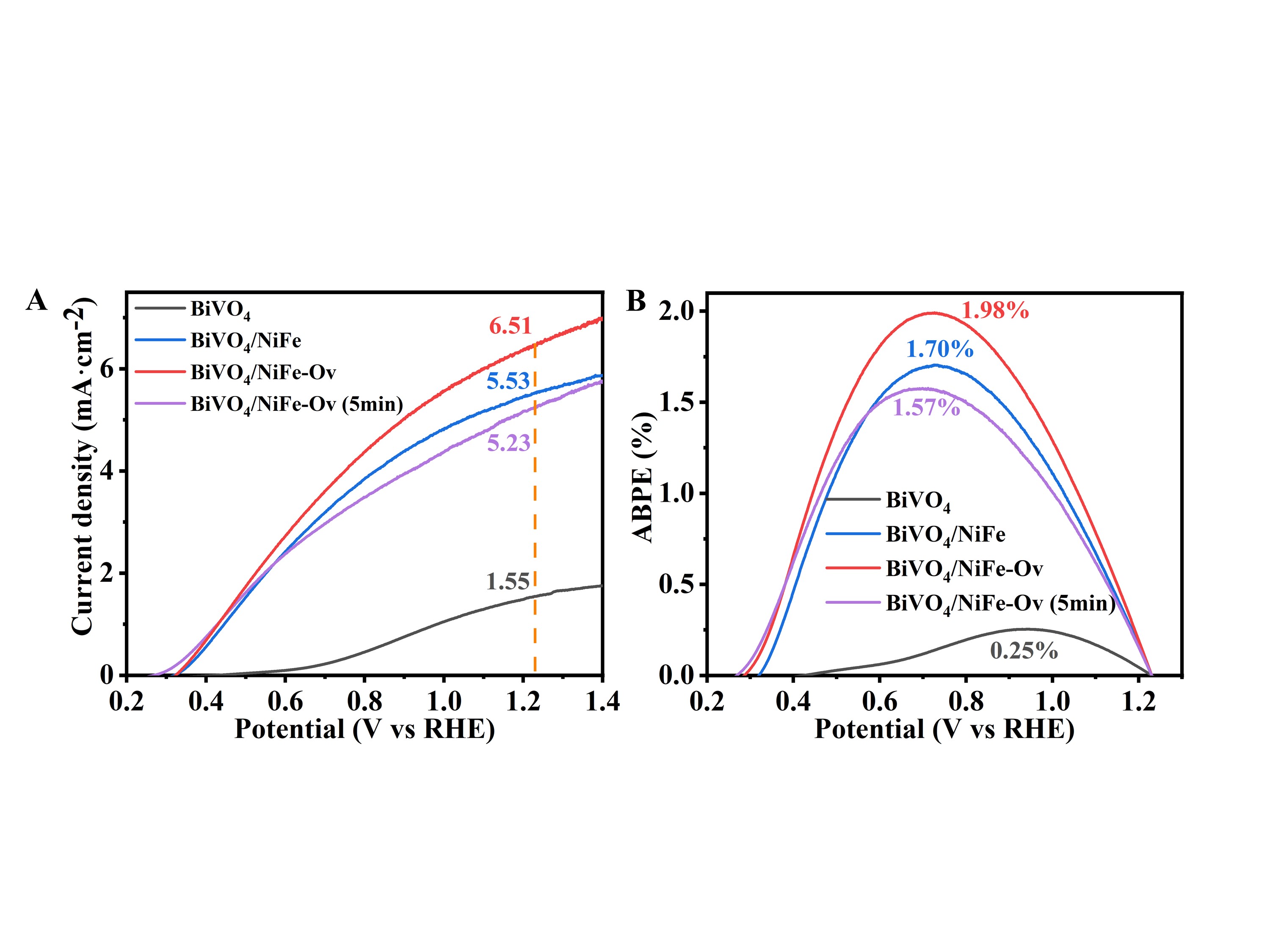


**Fig. S6** LSV (**A**) and ABPE (**B**) curves for BiVO_4_, BiVO_4_/NiFe, BiVO_4_/NiFe-Ov, and BiVO_4_/NiFe-Ov (5min) photoanodes measured in 0.5 M K_3_BO_3_ electrolyte

**Additional discussion**

Figure S6 shows the infuence of Ar-plasma treatment time on PEC water oxidation performance of BiVO_4_/NiFe photoanodes. At 1.23 V_RHE_, the BiVO_4_/NiFe-Ov (2 min) possesses a higher photocurrent density of 6.51 mA·cm^-1^ than BiVO_4_/NiFe (0 min, 5.53 mA·cm^-1^) and BiVO_4_/NiFe-Ov (5 min, 5.23 mA·cm^-1^) photoanodes, respectively. In addition, the BiVO_4_/NiFe-Ov also exihibits a highest ABPE value of 1.98% at 0.72 V_RHE_. This results indicate the Ar-plasma treatment time on NiFeOOH catalyst play a crucial roles in PEC activity of BiVO_4_/NiFe photoanodes.


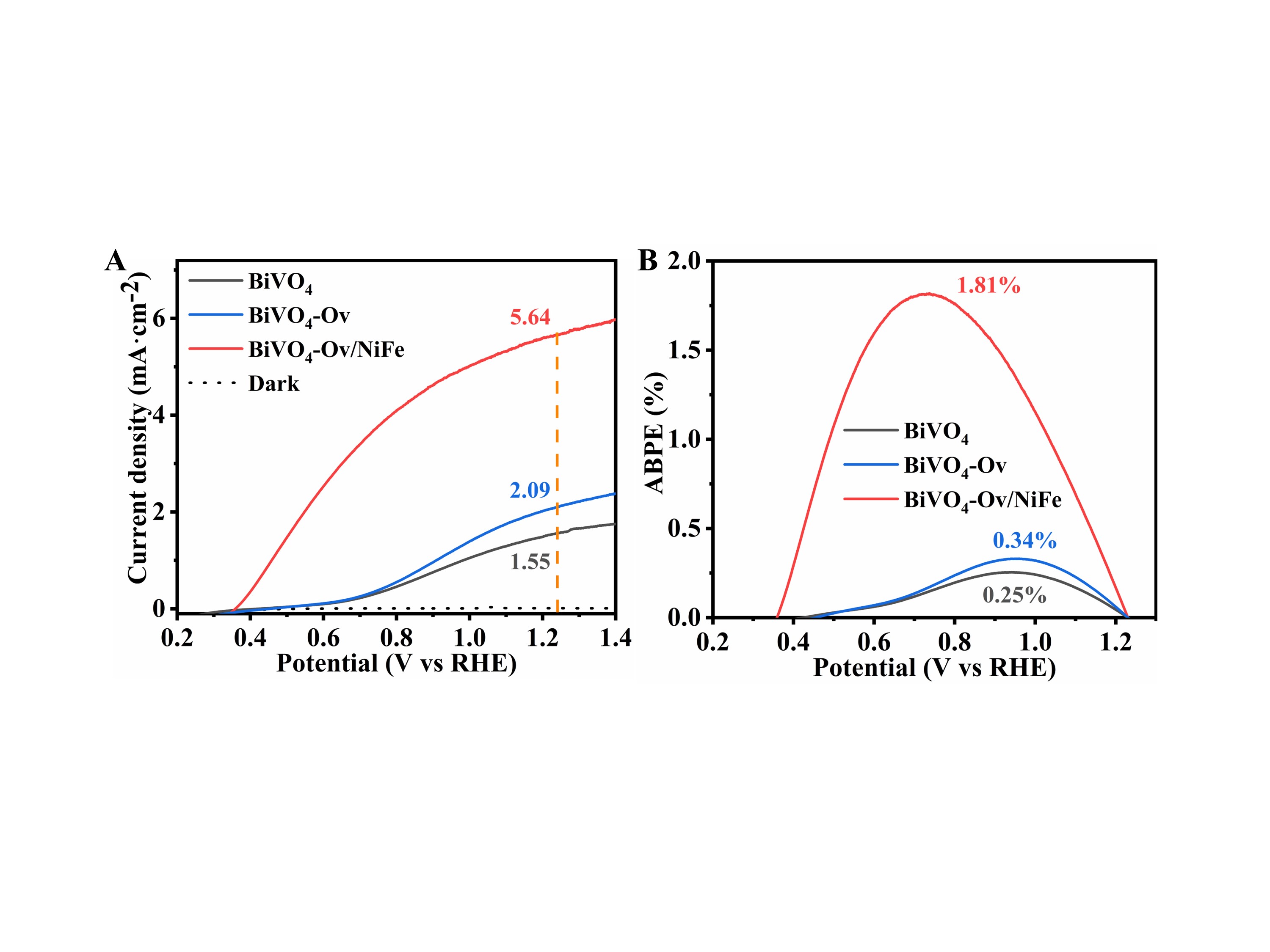


**Fig. S7** LSV (**A**) and ABPE (**B**) curves for BiVO_4_, BiVO_4_-Ov, and BiVO_4_-Ov/NiFe photoanodes measured in 0.5 M K_3_BO_3_ electrolyte

**Additional discussion**

The effects of Ar-plasma treatment on the PEC performances of pristine BiVO_4_ have also been studied. The photocurrent density of BiVO_4_-Ov (2.09 mA cm^-1^) is slightly higher than that of pristine BiVO_4_ (1.55 mA cm^-1^), while the photocurrent of BiVO_4_-Ov/NiFe (5.64 mA cm^-1^) is nearly consistent with BiVO_4_/NiFe (5.53 mA cm^-1^) but obviously lower than BiVO_4_/NiFe-Ov (6.51 mA cm^-1^). These results clearly indicate the improvement of PEC activity for BiVO_4_/NiFe-Ov should be mainly attributed to the oxygen vacancy formation on NiFe catalyst nanolayers instead of BiVO_4_.


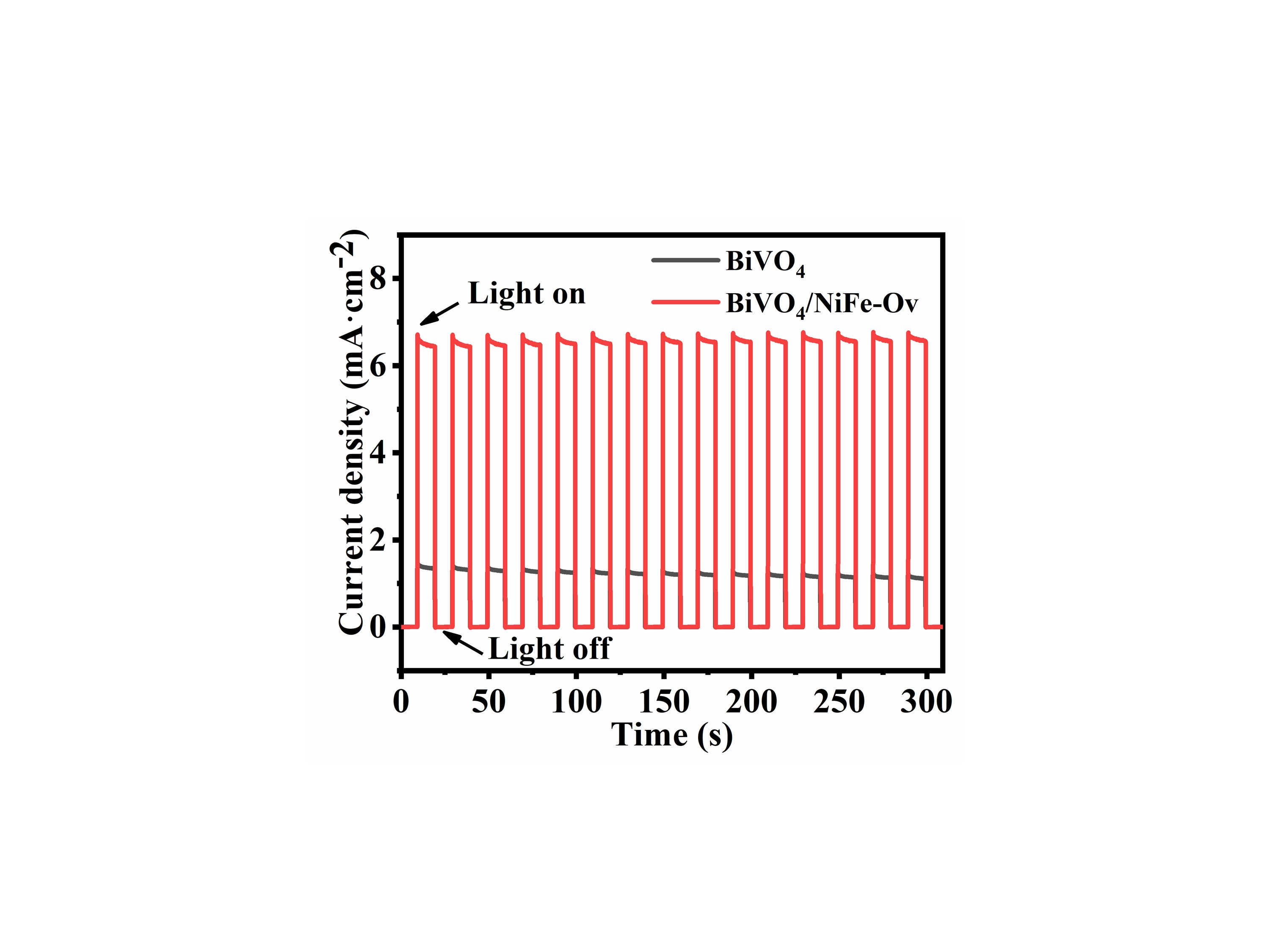


**Fig. S8** The chopped irradiation i-t curve of BiVO_4_ and BiVO_4_/NiFe-Ov photoanodes

**Additional discussion**

The chopped irradiation i-t curve exhibits the low charge recombination of BiVO_4_/NiFe-Ov compared with BiVO_4_ photoanodes.


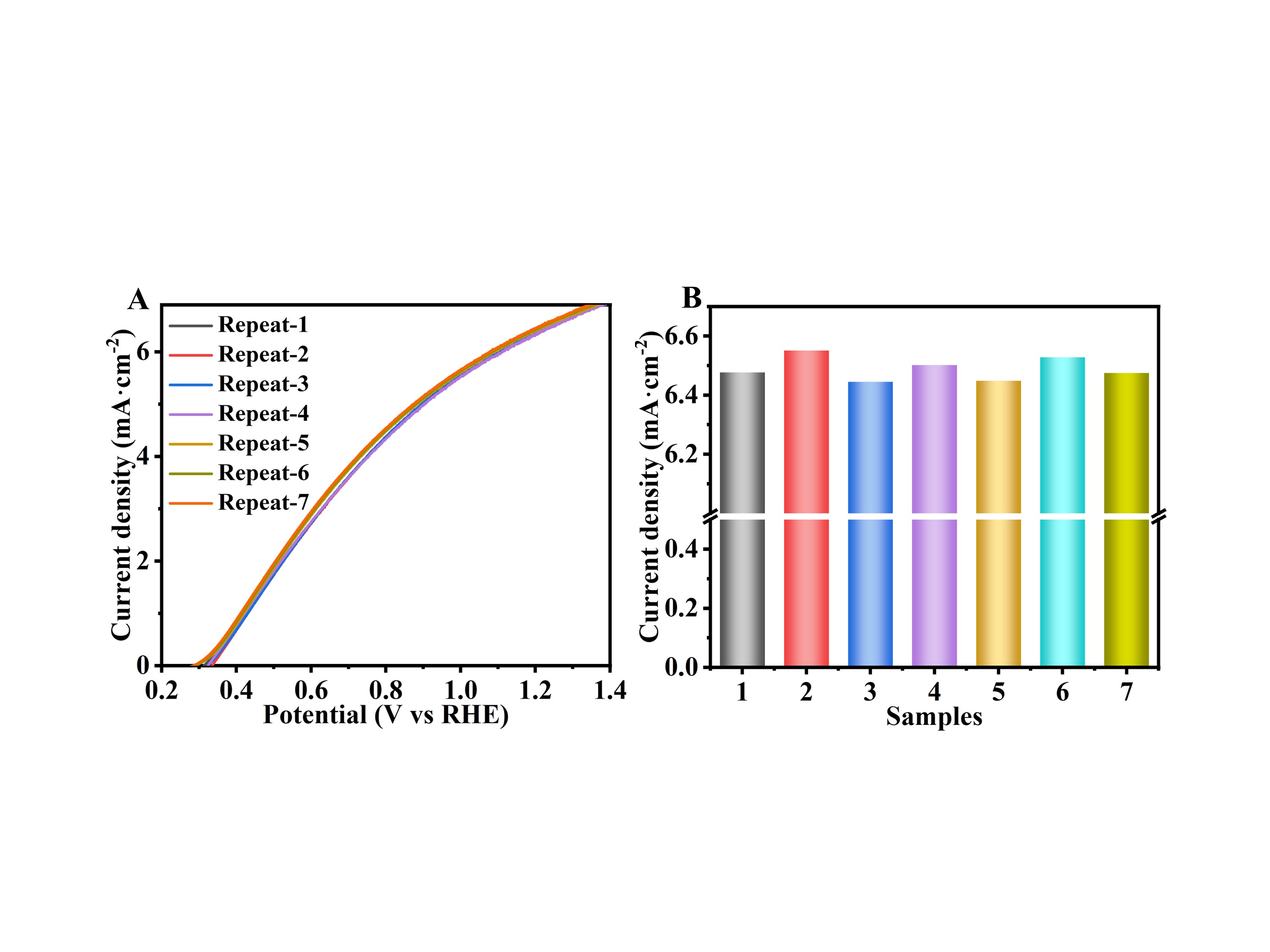


**Fig. S9** (**A**) Reproducibility of the LSV curves of the BiVO_4_/NiFe-Ov photoanodes, and (**B**) the statistics of the corresponding photocurrent at 1.23 V_RHE_

**Additional discussion**

The LSV curves for seven prepared BiVO_4_/NiFe-Ov photoanodes under the same conditions have been measured (Fig. S9A), and their current densities at 1.23 V_RHE_ have been summarized in Fig. S9B. Obviously, the obtained average current density of 6.51 mA cm^-2^ at 1.23 V_RHE_, suggesting the relatively high repeatability of BiVO_4_/NiFe-Ov photoanodes.


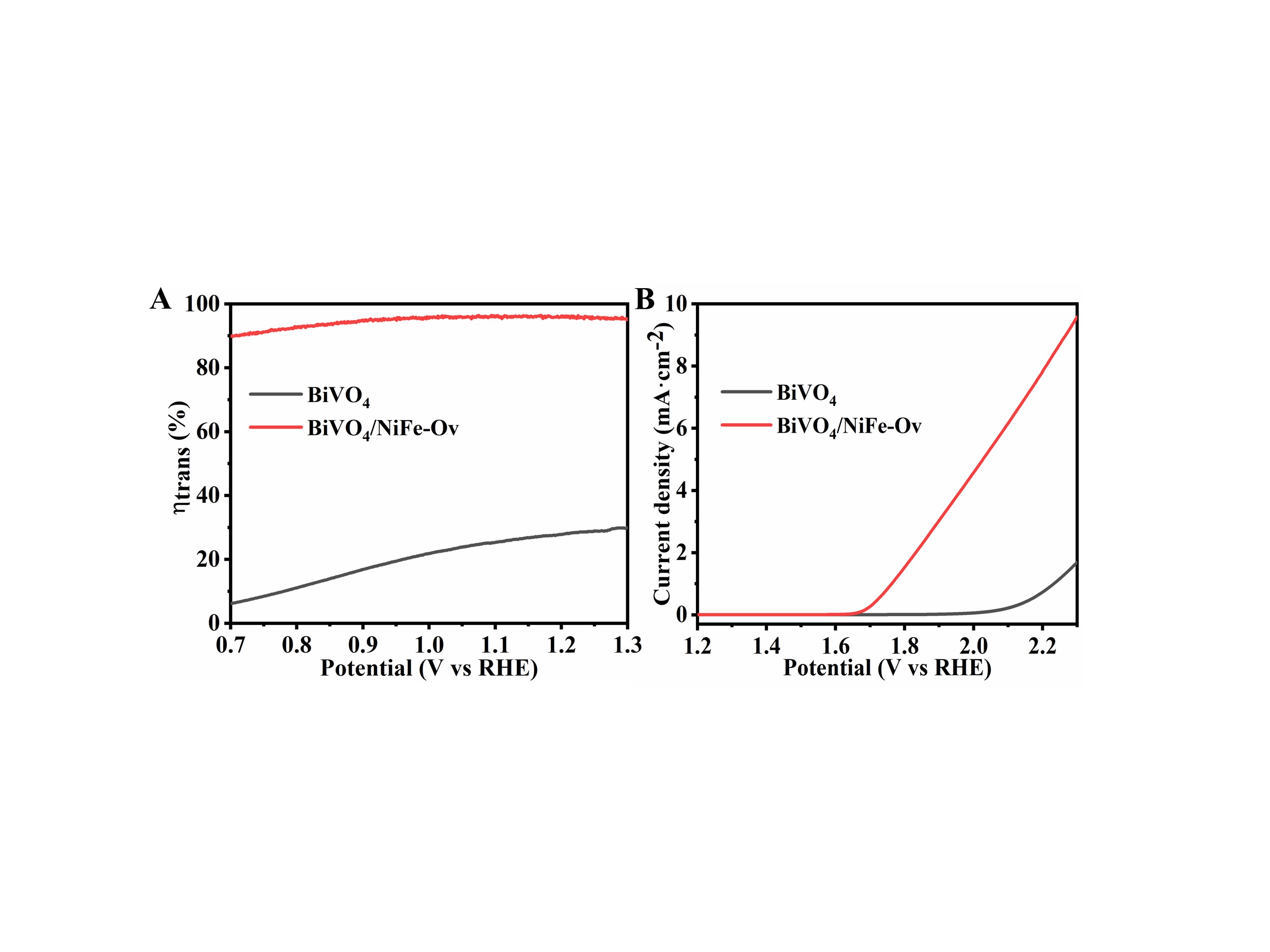


**Fig. S10** (**A**) The charge transfer efficiencies (η_trans_) and (**B**) LSV curves in the dark of BiVO_4_ and BiVO_4_/NiFe-Ov photoanodes

**Additional discussion**

As shown in Fig. S10A, the interfacial charge separation efficiencies (η_trans_) are important for PEC water oxidation, which was explored by using 0.2 M Na_2_SO_3_ as a hole scavenger. According to the equation of J_water_/J_sulfite_ from the LSV curves, a much higher charge separation efficiency have been obtained on the BiVO_4_/NiFe-Ov compared with that of the BiVO_4_ photoanodes, indicating the crucial roles of the NiFe-Ov catalyst for promoting the interfacial charge separation and hole transport. Moreover, the electrochemical OER properties under darkness have also been investigated and shown in Fig. S10B. Obviously, BiVO_4_/NiFe-Ov exhibits a lower overpotential and higher water oxidation current density compared with BiVO_4_, further demonstrating the outstanding OER performance of the BiVO_4_/NiFe-Ov photoanodes.


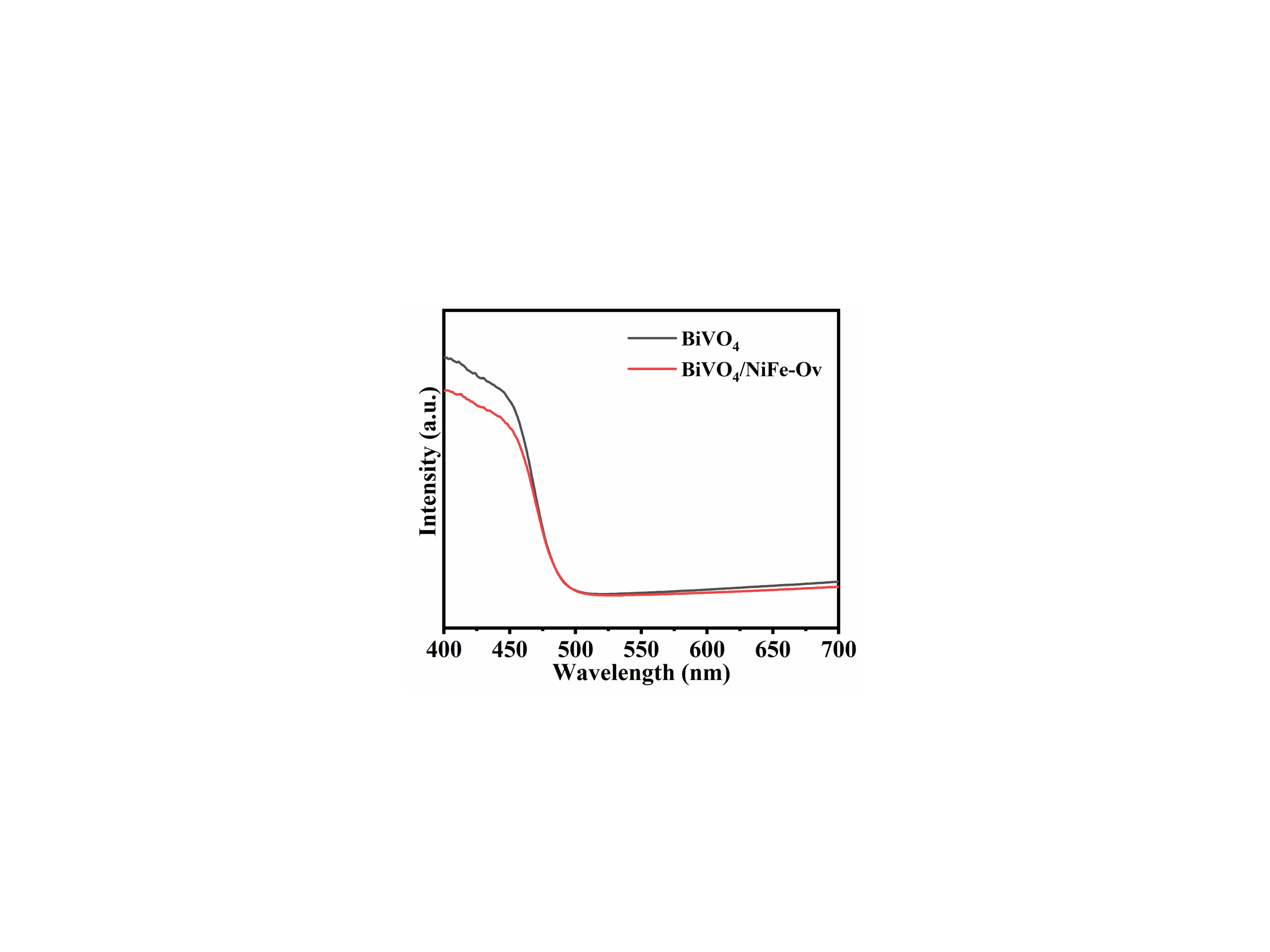


**Fig. S11** UV-vis diffuse reflectance spectra of BiVO_4_ and BiVO_4_/NiFe-Ov photoanodes

**Additional discussion**

Figure S11 shows the UV-vis diffuse reflectance spectra of the BiVO_4_ and BiVO_4_/NiFe-Ov photoanodes, and no obvious change for the absorption band-edge could be observed, varifing that the decoration of NiFe-Ov catalyst has no evident effect on the optical absorption properties of BiVO_4_ photoanodes.


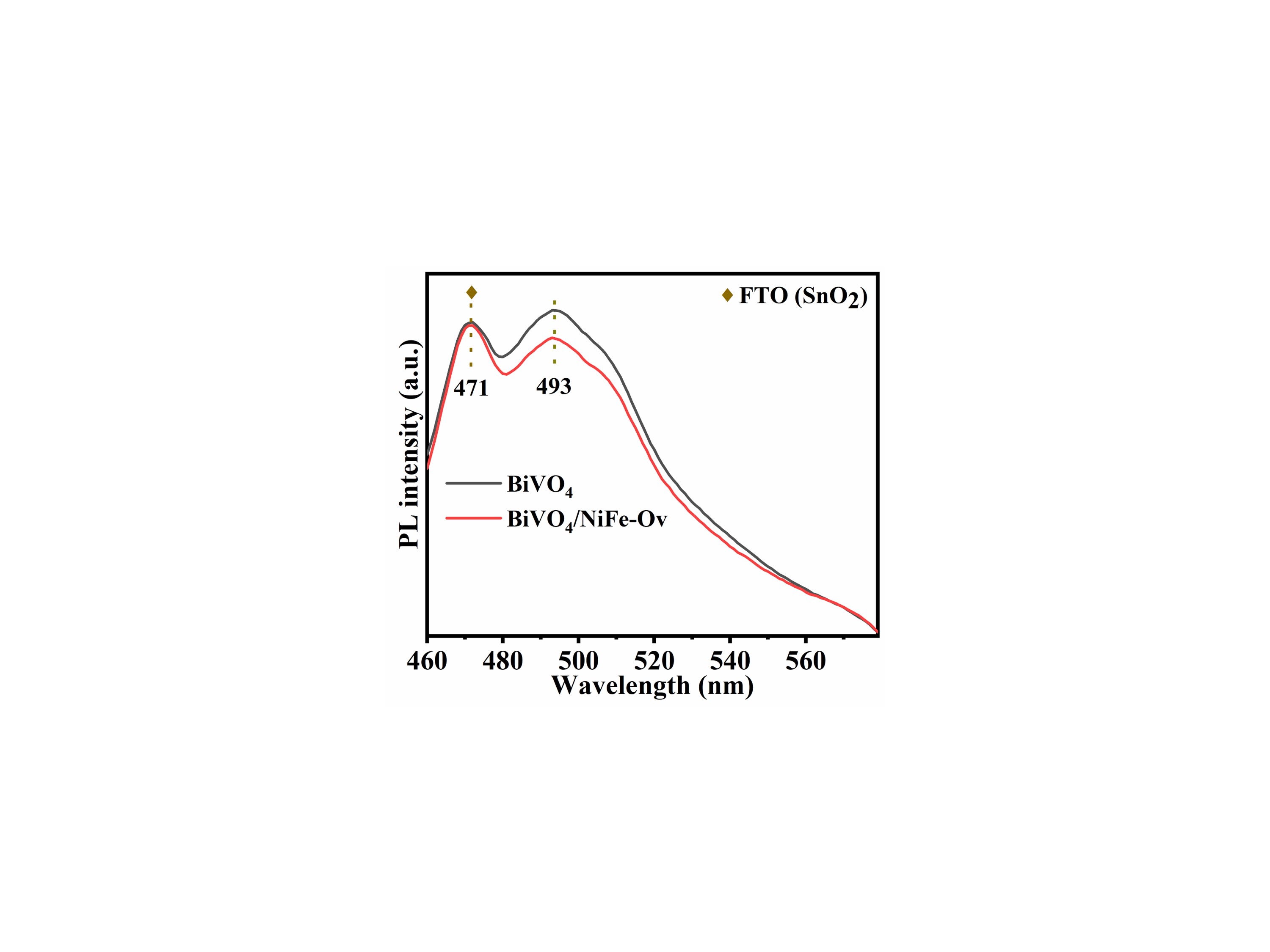


**Fig. S12** Photoluminescence spectra of BiVO_4_ and BiVO_4_/NiFe-Ov photoanodes

**Additional discussion**

As shown in Fig. S12, to further explore the charge separation capability, the Photoluminescence spectras (PL) of the BiVO_4_ and BiVO_4_/NiFe-Ov photoanodes were measured under laser excitation of 355 nm. Obviously, two PL peaks at 471 and 493 nm have been detected, which assigned to FTO (SnO_2_) substrate and the radiative recombination of hole in O 2p band and electron in V 3d band for BiVO_4_, respectively [S2, S3]. A much weaker PL peak intensity has been obtained on BiVO_4_/NiFe-Ov compared with that of the BiVO_4_ photoanodes, demonstrating the relatively low electron-hole recombination after decorating NiFe-Ov catalyst on BiVO_4_. Therefore, BiVO_4_/NiFe-Ov photoanodes possess more efficient capability of charge separation.


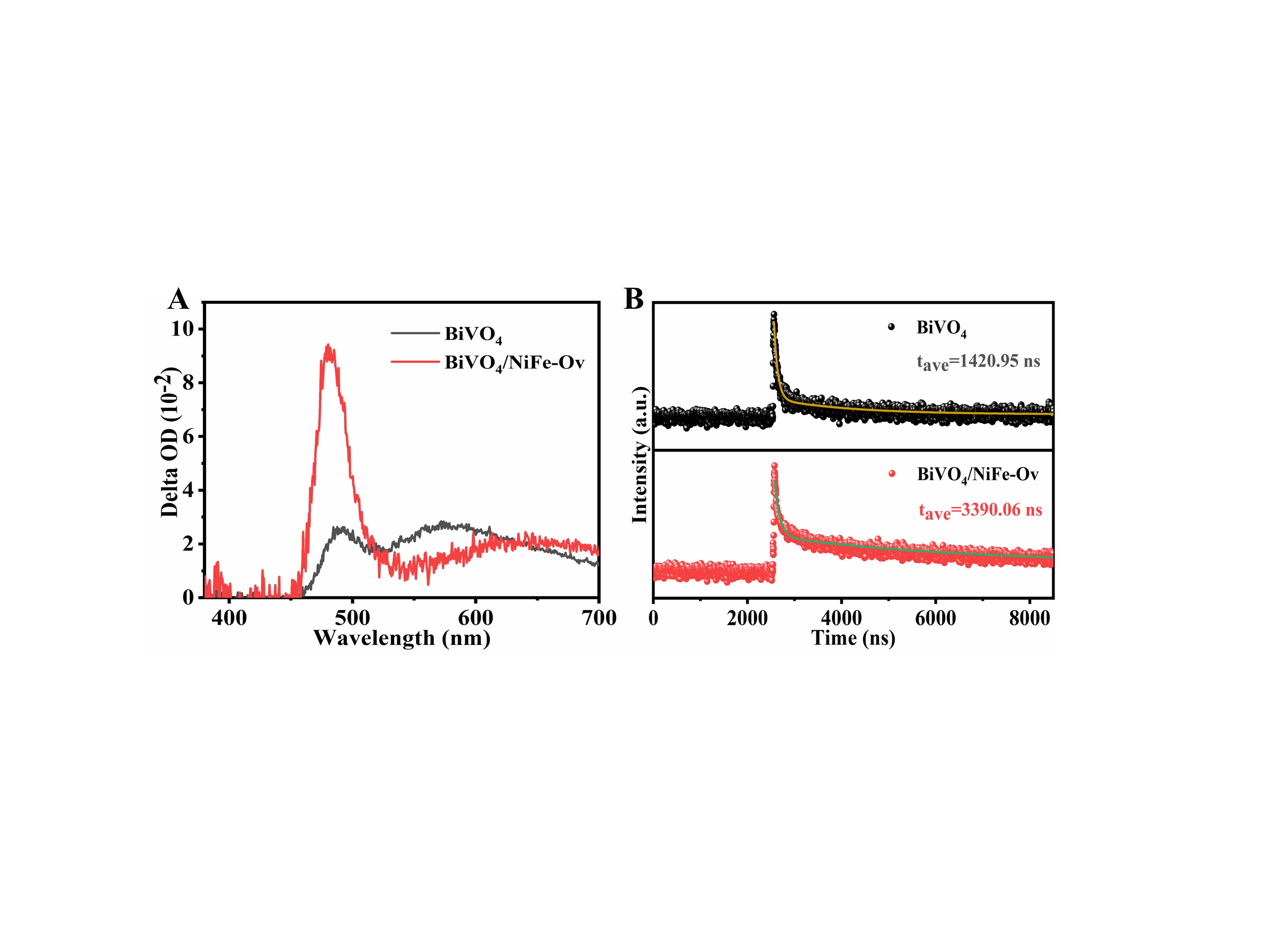


**Fig. S13** (**A**) Transient absorption (TA) spectra and (**B**) time-resolved TA curves of BiVO_4_ and BiVO_4_/NiFe-Ov photoanodes

**Additional discussion**

The energy relaxation process and charge transfer behavior under excited states have been evaluated by the time-resolved transient absorption spectra (TR-TAS). Fig. S13A shows that the BiVO_4_/NiFe-Ov photoanodes exhibit a higher peak intensity than BiVO_4_, indicating that the photo-induced absorption processes have been significantly improved after the decoration of the NiFe-Ov catalysts. As shown in Fig. S13B and Table S2, the BiVO_4_/NiFe-Ov photoanodes possess a longer carrier lifetime compared with BiVO_4_ photoanodes. Based on the above analysis, it can be concluded that the NiFe-Ov catalysts exhibit a preferable capability for promoting charge separation and extending the carrier lifetimes.





**Fig. S14** The spectra of SPECM surface tip currents of the BiVO_4_ photoanode with and without NiFe-Ov catalysts under light irradiation

**Additional discussion**

To further investigate the surface photoactive properties, scanning photoelectrochemical microscopy (SPECM) has been conducted. As shown in Fig. S14, a relatively low photocurrent was obtained under light illumination on the pristine BiVO_4_ regions, while the photocurrent drastically improved on the BiVO_4_/NiFe-Ov regions. This result clearly indicates that BiVO_4_/NiFe-Ov possesses the higher water oxidation capability than BiVO_4_, which is line with their PEC water oxidation activities.


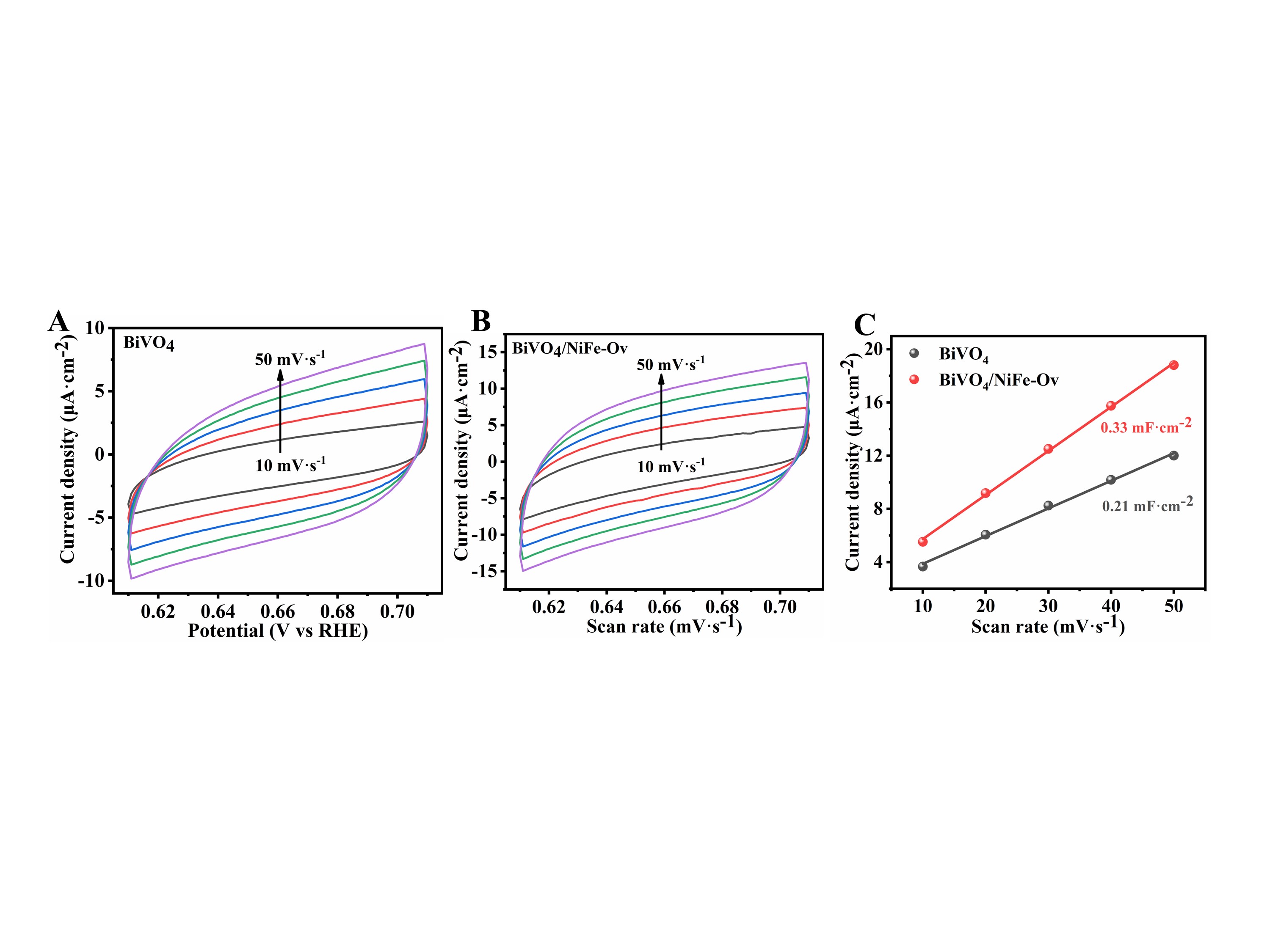


**Fig. S15** The cyclic voltammetry curves of (A) BiVO_4_, and (B) BiVO_4_/NiFe-Ov with the scan rates from 0.01 to 0.05 V/s, and (C) the electrochemical double layer capacitance of BiVO_4_ and BiVO_4_/NiFe-Ov

**Additional discussion**

To explore the electrochemical active surface area, cyclic voltammetry curves have been performed at 0.66 V_RHE_ in 0.5 M K_3_BO_3_ under dark conditions. As shown in Fig. S15, the electrochemical double layer capacitance (C_dl_) of BiVO_4_ and BiVO_4_/NiFe-Ov have been achieved to 0.21 and 0.33 mF·cm^-2^, respecyively. This result confirms the significantly increases the surface active sites of BiVO_4_ photoanodes after the decoration of NiFe-Ov catalyst.


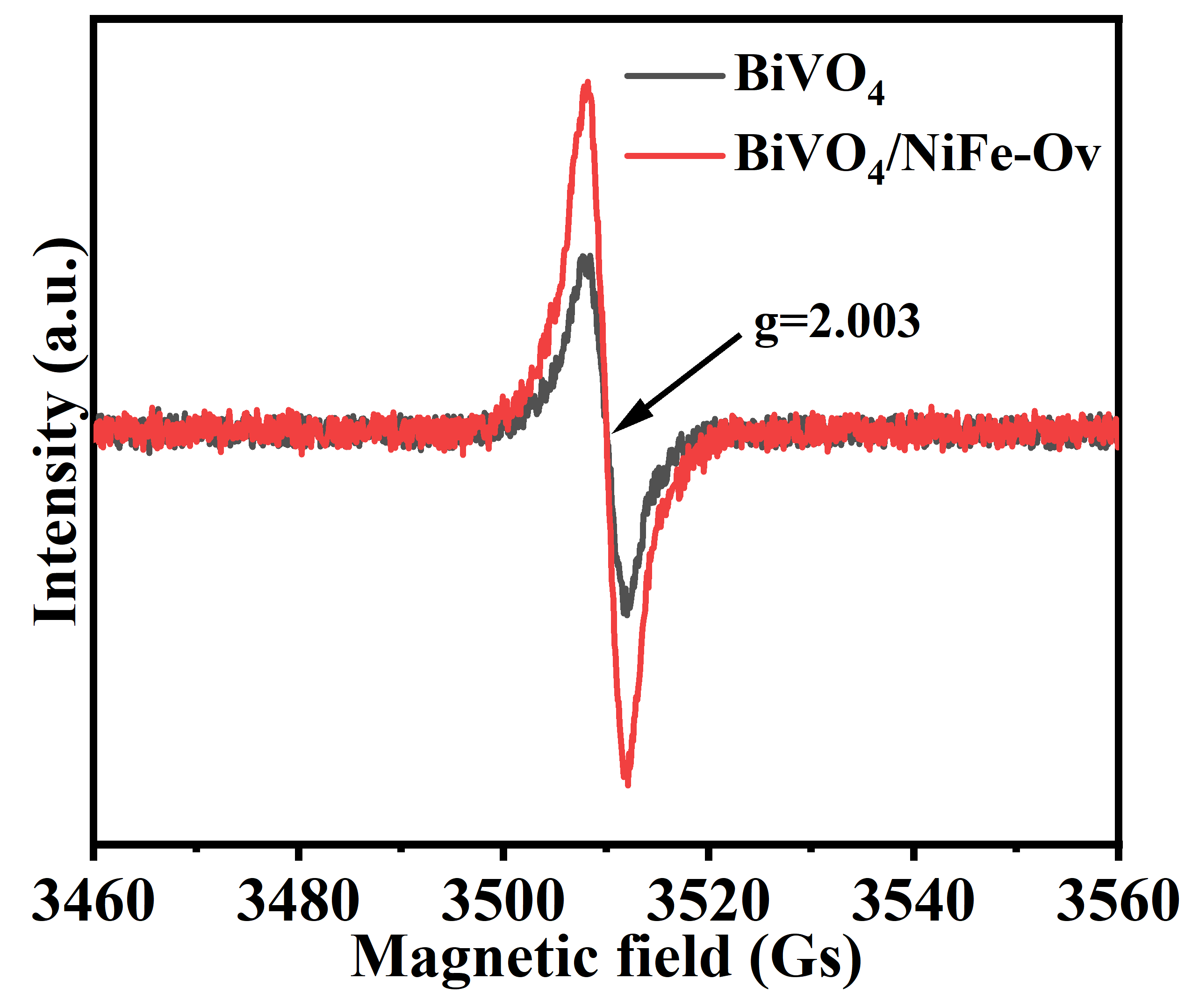


**Fig. S16** Room-temperature EPR spectra of BiVO_4_ and BiVO_4_/NiFe-Ov

**Additional discussion**

The oxygen vacancies were further investigated by electron paramagnetic resonance (EPR) spectroscopy (Fig. S16). Compared with BiVO_4_ photoanodes, BiVO_4_/NiFe-Ov exhibits a significantly enhanced EPR signal at g = 2.003, indicating that more oxygen vacancies should be formed in NiFe-Ov catalysts.


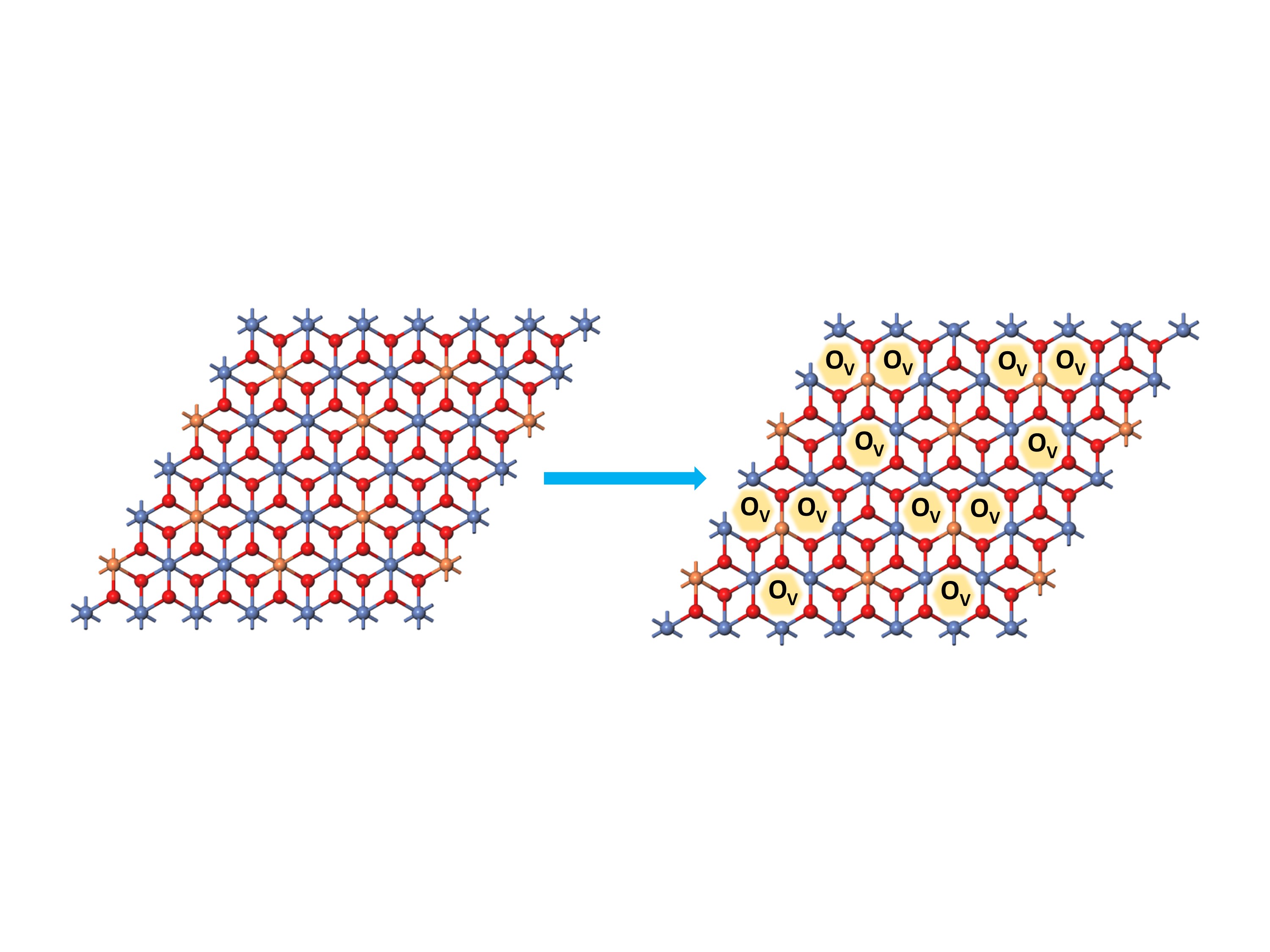


**Scheme S2** Diagram of NiFeOOH and NiFe-Ov, Ni (blue), Fe (orange), O (red)

**Additional discussion**

According to the XPS O 1s spectra and EPR result (Fig. 1F and Fig. S16), the BiVO_4_/NiFe-Ov photoanodes possess the abundant oxygen vacancies. Additionally, as shown in Scheme. S2, the coordination number of NiFe catalyst could be reduced in the presence of oxygen vacancies


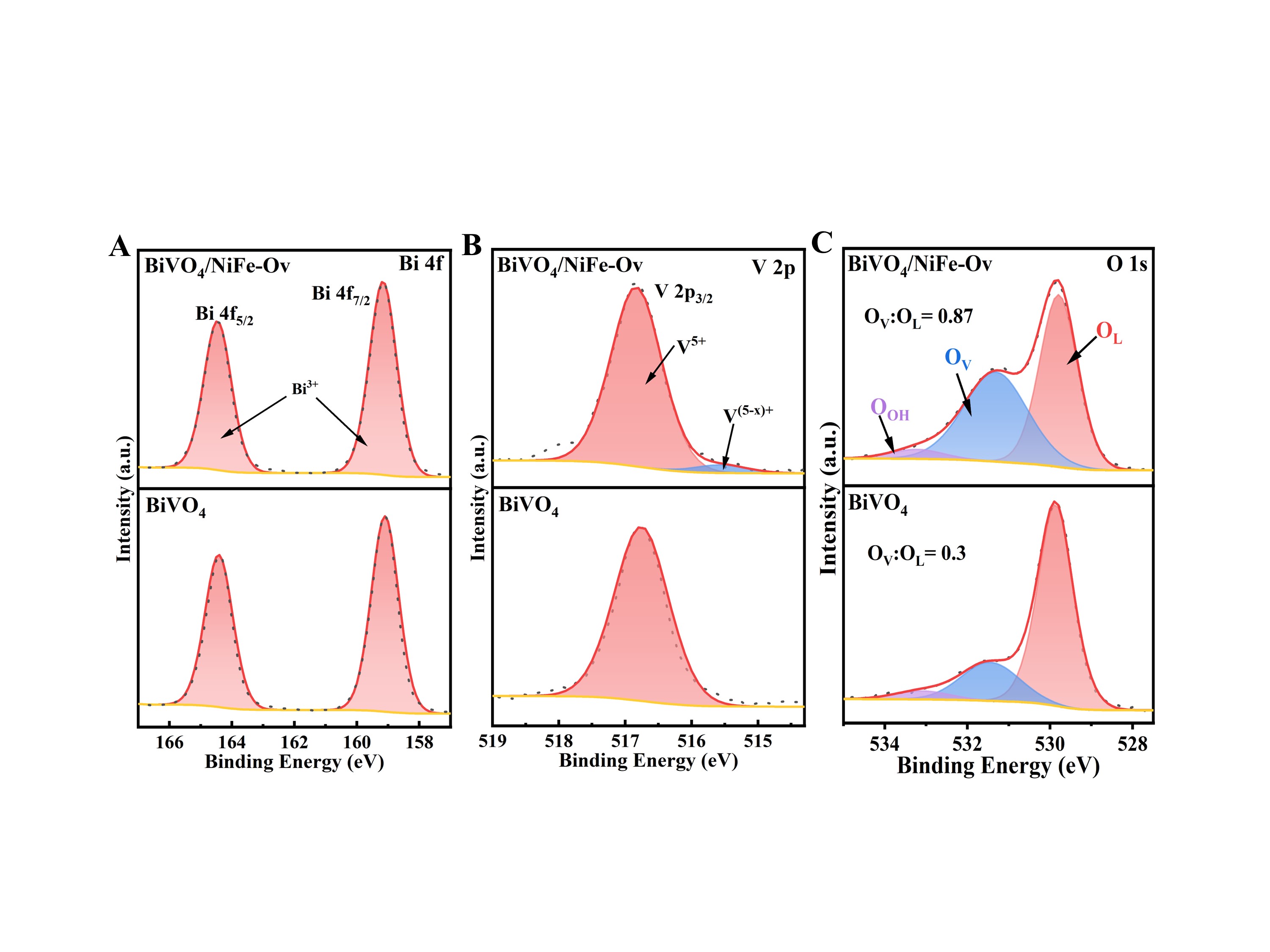


**Fig. S17** XPS high-resolution (**A**) Bi 4f, (**B**) V 2p and (**C**) O 1s spectra of BiVO_4_ and BiVO_4_/NiFe-Ov photoanodes

**Additional discussion**

The chemical states and electronic structures of the BiVO_4_/NiFe-Ov photoanodes were investigated by high-resolution X-ray photoelectron spectroscopy (XPS). The evidently changes of Bi 4f, V 2p, and O 1s peaks were observed for BiVO_4_/NiFe-Ov photoanodes compared to BiVO_4_ (Fig. S17). Specifically, for BiVO_4_/NiFe-Ov, the peak at binding energy of 159.1 and 164.3 eV for Bi 4f spectra, attributed to the Bi^3+^ species, while a shoulder peak at 515.5 eV for V 2p spectra, assinged to the low-valence V^(5-x)+^ species caused by the injection of eletrons from electron-rich NiFe-Ov to BiVO_4_. In addition, the peaks of O 1s spectra at 529.8, 531.2, and 532.4 eV could be assinged to the lattice oxygens (O_L_), oxygen vacancies (O_V_), hydroxyl oxygens (O_OH_), respectively. Apparently, the intensity of Ov peak has remarkedly increased for BiVO_4_/NiFe-Ov photoanodes, indicating the nature of the BiVO_4_/NiFe-Ov photoanodes with abundant oxygen vacancies.


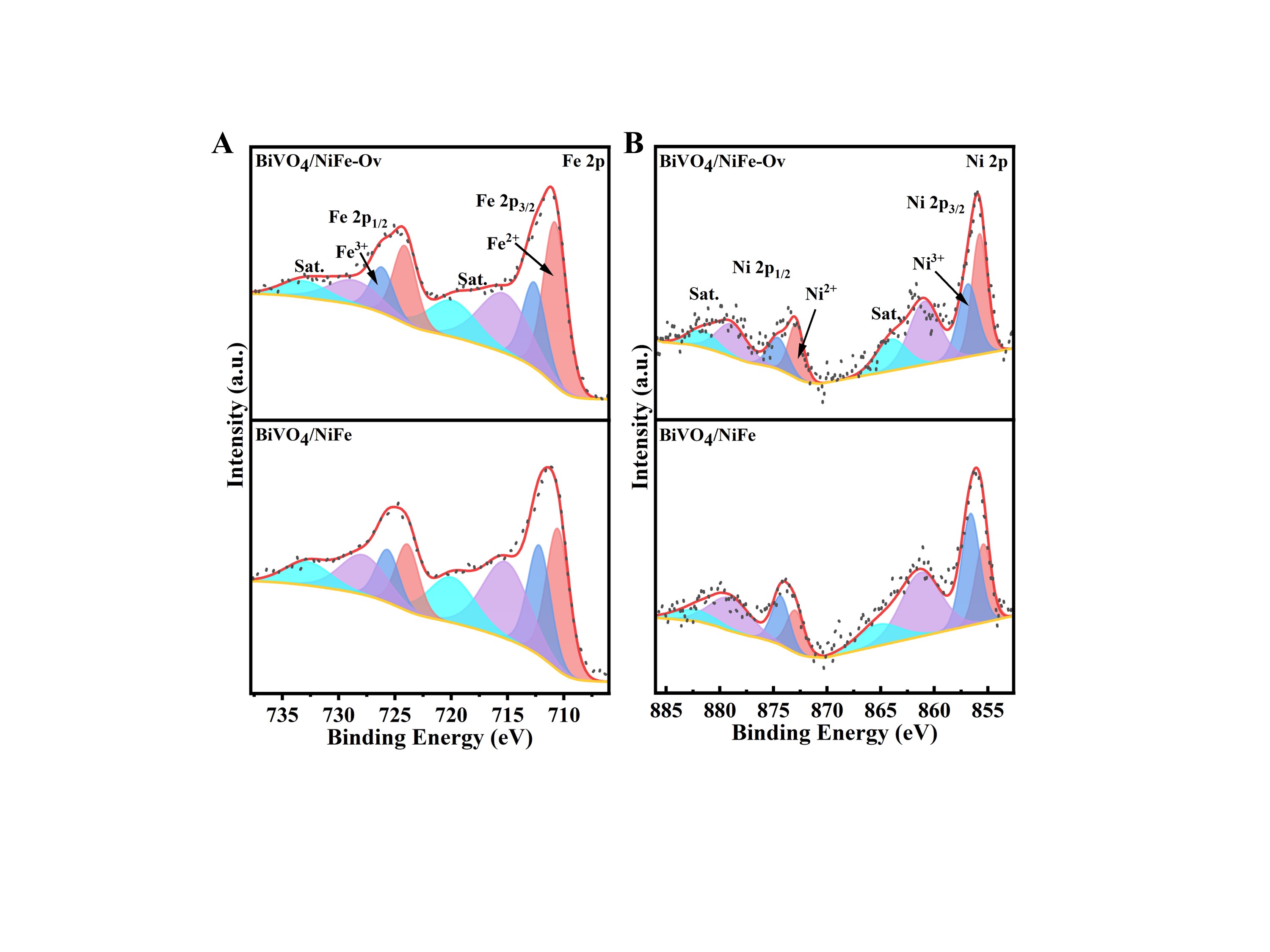


**Fig. S18** XPS high-resolution (A) Fe 2p and (B) Ni 2p spectra of BiVO_4_/NiFe and BiVO_4_/NiFe-Ov photoanodes


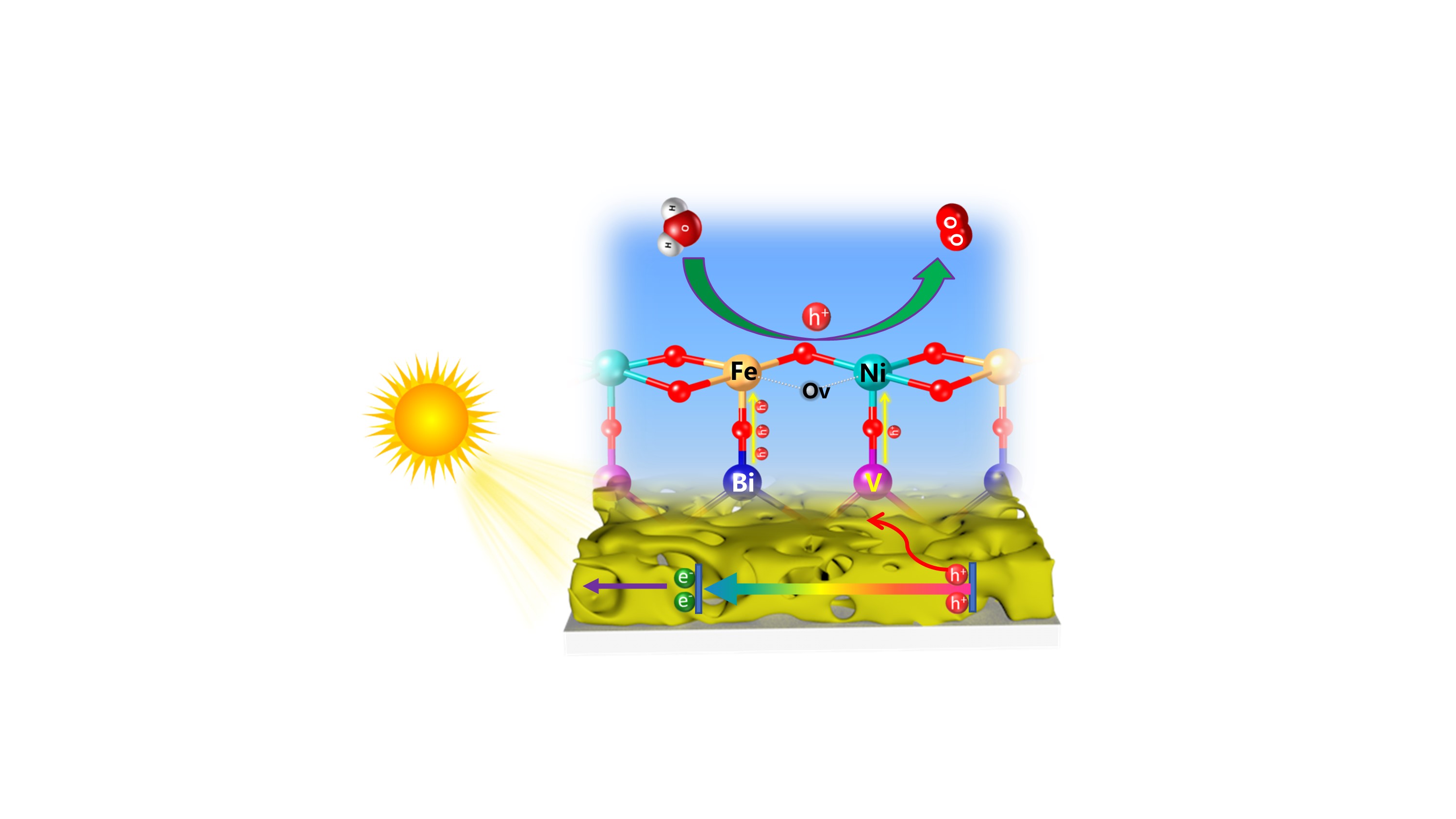


**Scheme S3** Diagram of the PEC water oxidation process on BiVO_4_/NiFe-Ov photoanodes


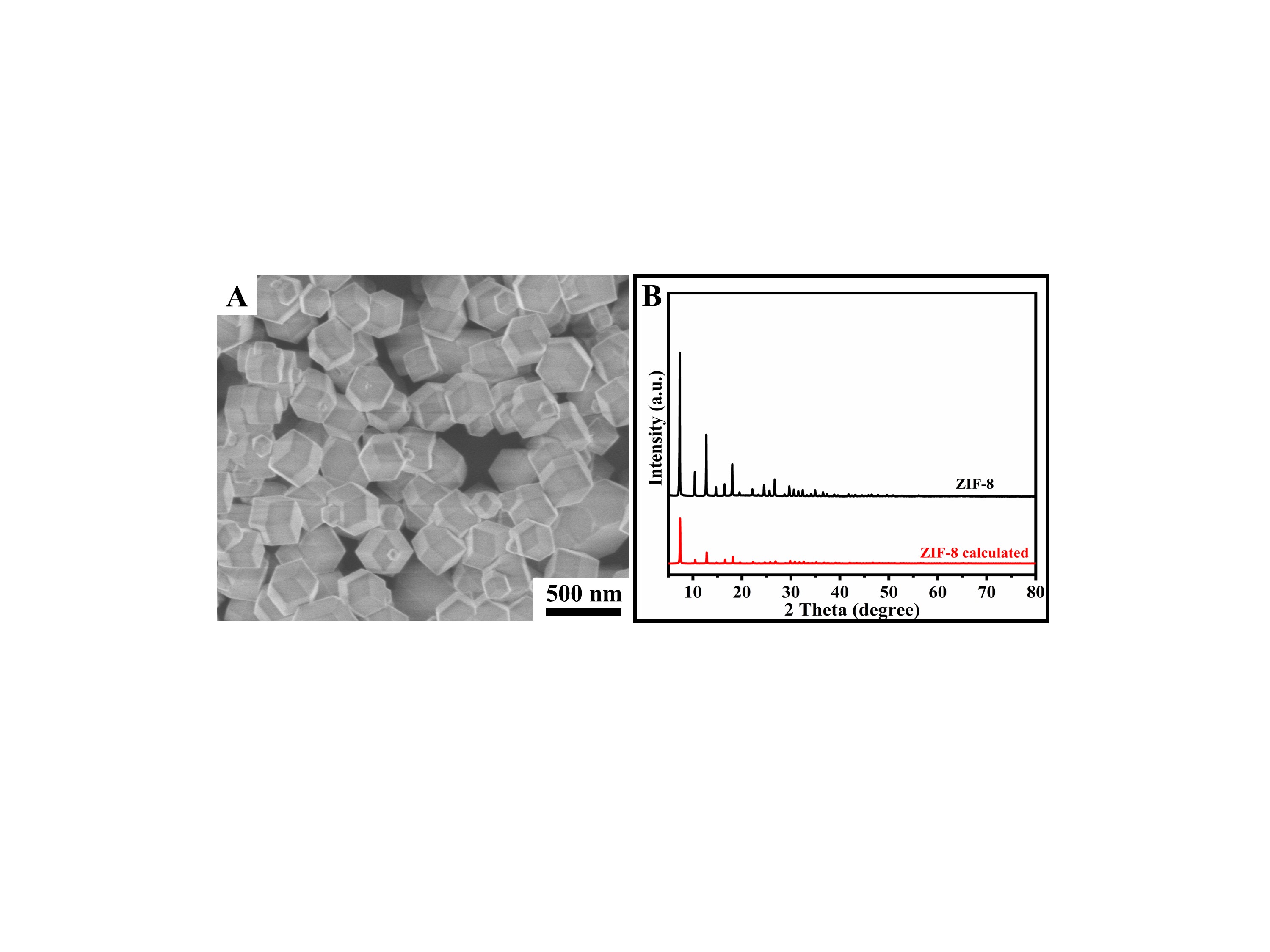


**Fig. S19** (**A**) SEM image and (**B**) XRD pattern of prepared ZIF-8

**Additional discussion**

As a precursor of N-C substrate, ZIF-8 was prepared by a simple stirring process at room trmperature. As shown in Fig. S19A, ZIF-8 exhibits an uniform rhombic dodecahedron morphology with an average size of ~250 nm. And the XRD characterize diffraction patterns of prepared ZIF-8 have been consistent with the standard ZIF-8 calculated by the cif data.


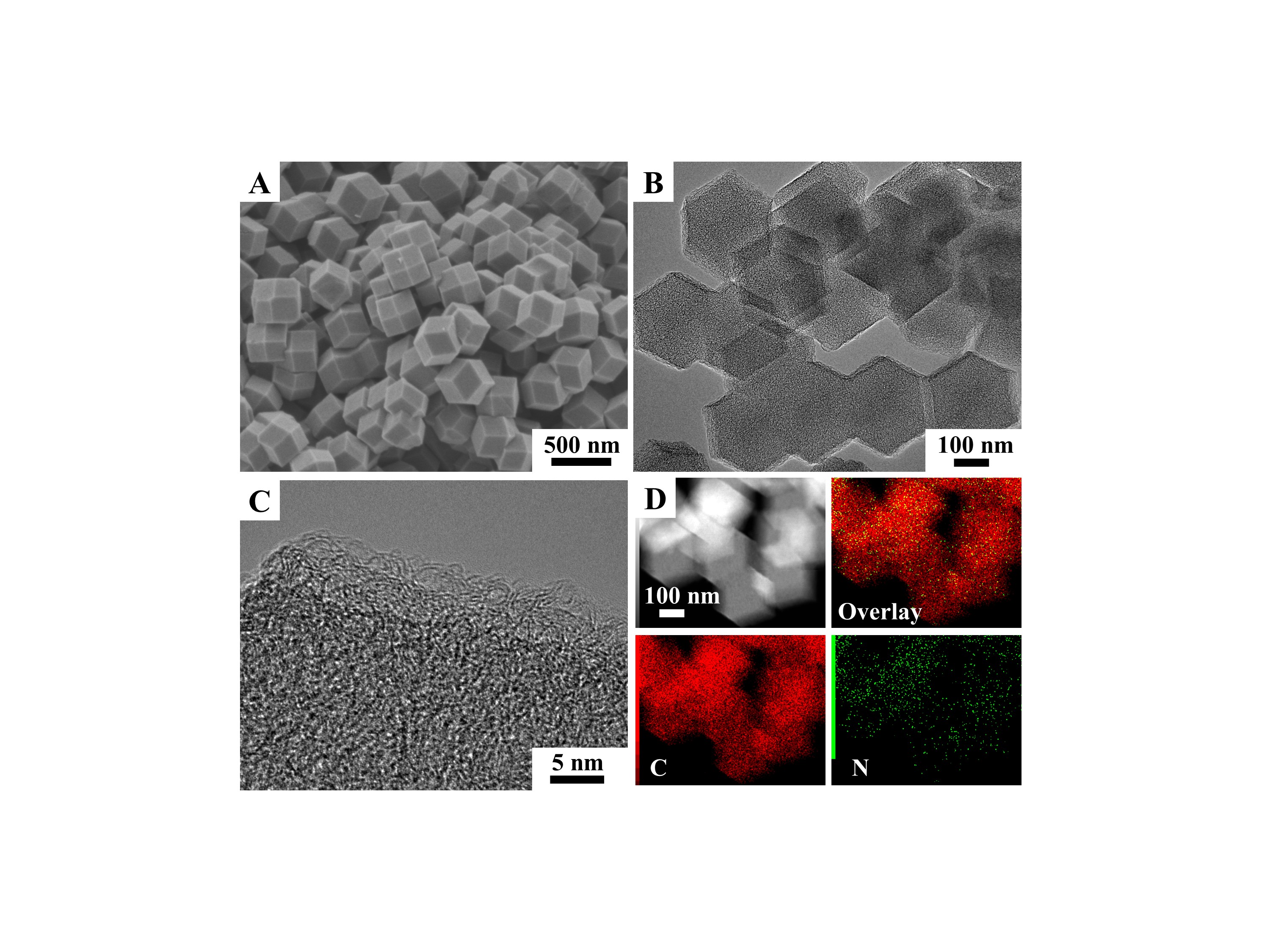


**Fig. S20** (**A**) SEM, (**B**) TEM, (**C**) HR-TEM, and (**D**) EDS Mapping images of NC
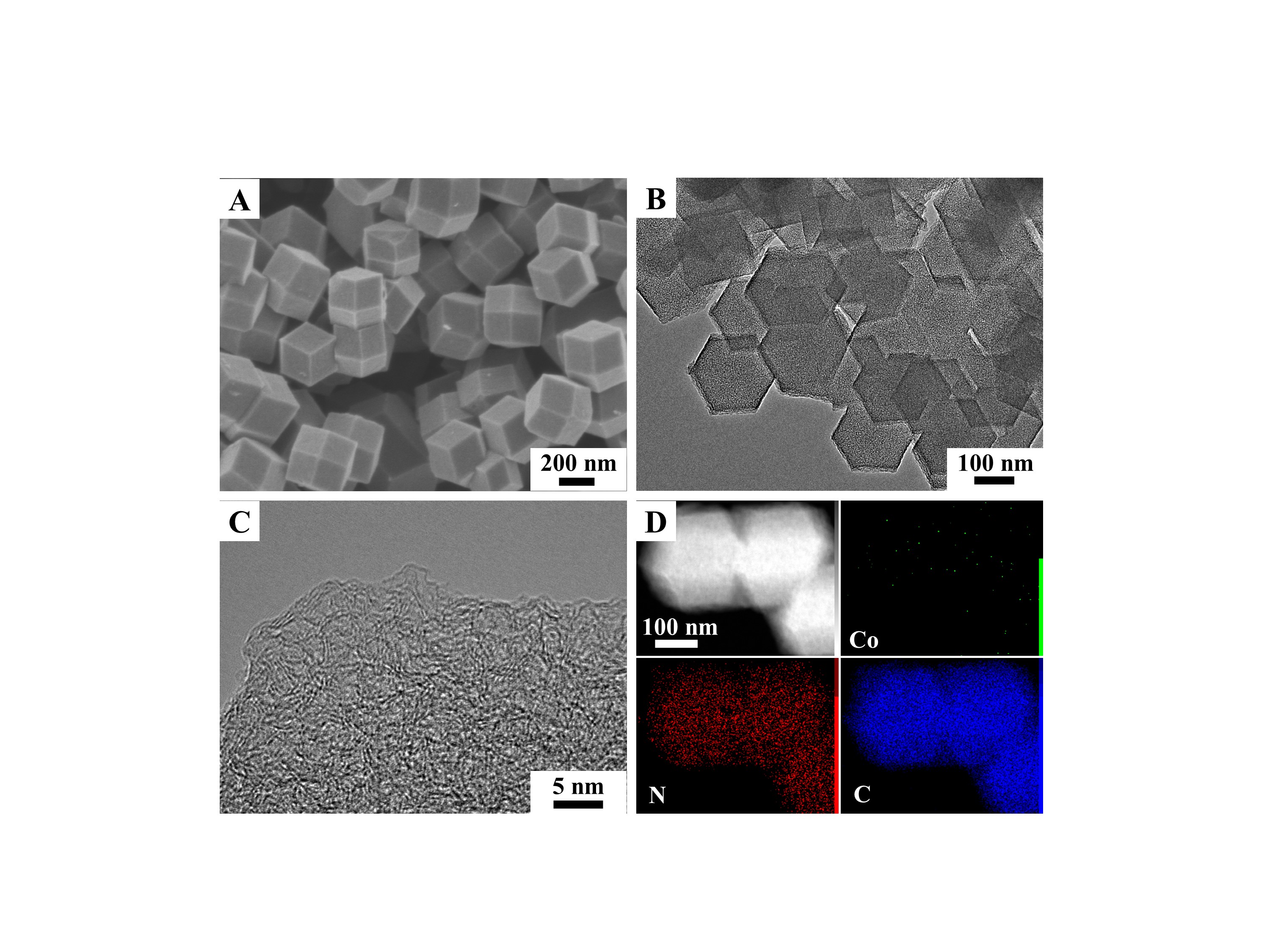


**Fig. S21** (**A**) SEM, (**B**) TEM, (**C**) HR-TEM, and (**D**) EDS Mapping images of CoPc-NC


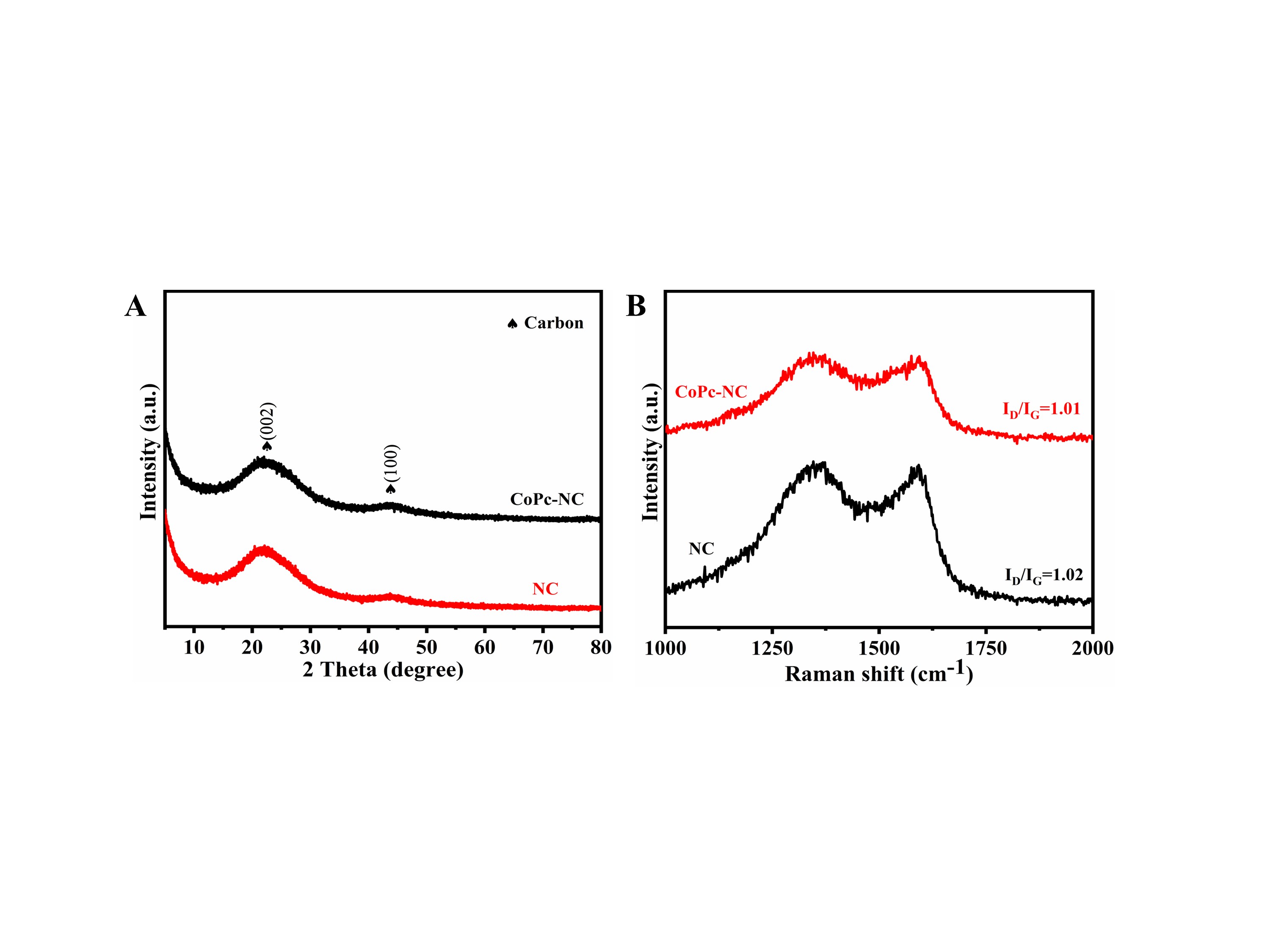


**Fig. S22** (**A**) XRD pattern and (**B**) Raman spectra of NC and CoPc-NC

**Additional discussion**

As shown in Fig. S22A, two broad peaks at 23° and 44° could be correspond to the (002) and (100) planes of graphitic carbon, respectively [S4]. No diffraction peaks of CoPc are observed, indicating no aggreation of CoPc on NC substrate. In Raman spectra (Fig. S22B), the peak at 1330 cm^-1^ is assigned to the D-band, which is commonly releated to the structural defects and disorder of carbon. While the G-band (1590 cm^-1^) usually reveals the existence of graphite structure in carbon materials. The near I_D_/I_G_ ratios for NC and CoPc-NC demonstrate the similar graphitized structure.


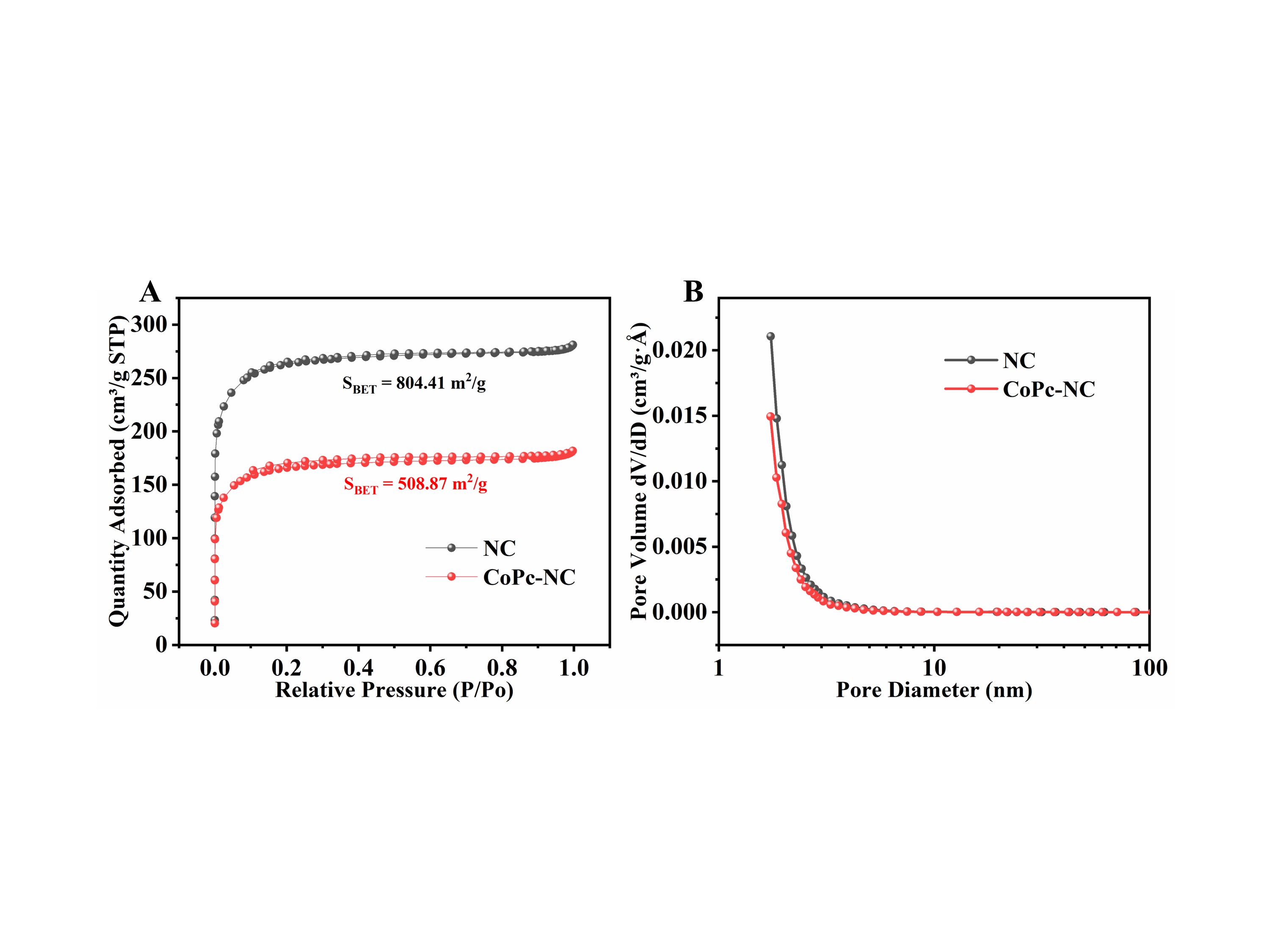


**Fig. S23** (**A**) N_2_ adsorption-desorption isotherm and (**B**) Pore size distribution of NC and CoPc-NC

**Additional discussion**

The textures of NC and CoPc-NC have been evaluated by N_2_ adsorption-desorption isotherm measurement. As shown in Fig. S23A, the Brunauer-Emmett-Teller (BET) surface areas of NC and CoPc-NC are 804.41 m^2^/g and 508.87 m^2^/g, respectively. The decrease of surface area for CoPc-NC compared to NC could be attributed to the incorporation of CoPc species into the channels of NC substrates. Additionally, the pore size distribution plots of NC and CoPc-NC indicate a narrow distribution centered at about 2 nm (Fig. S23B).


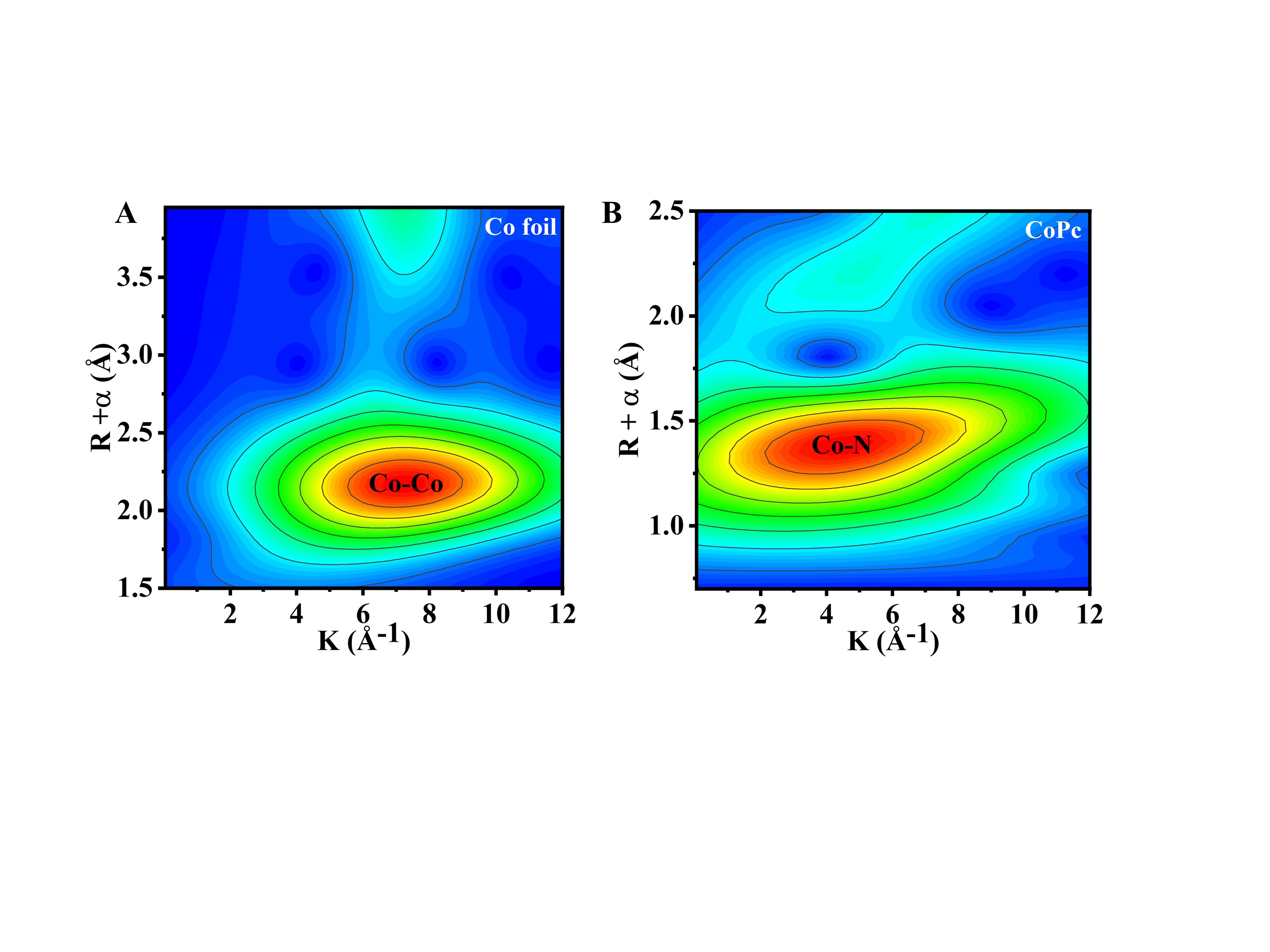


**Fig. S24** Fourier transformation of EXAFS analysis of (**A**) Co foil and (**B**) CoPc

**Additional discussion**

For comparion, the Fourier transformation of EXAFS analysis of Co foil and CoPc have shown in Fig. S24. The maximum intensity of WT contour plots for Co-Co bonds and Co-N bonds is located at 7.2 Å^-1^ and 4.3 Å^-1^, respectively.

**
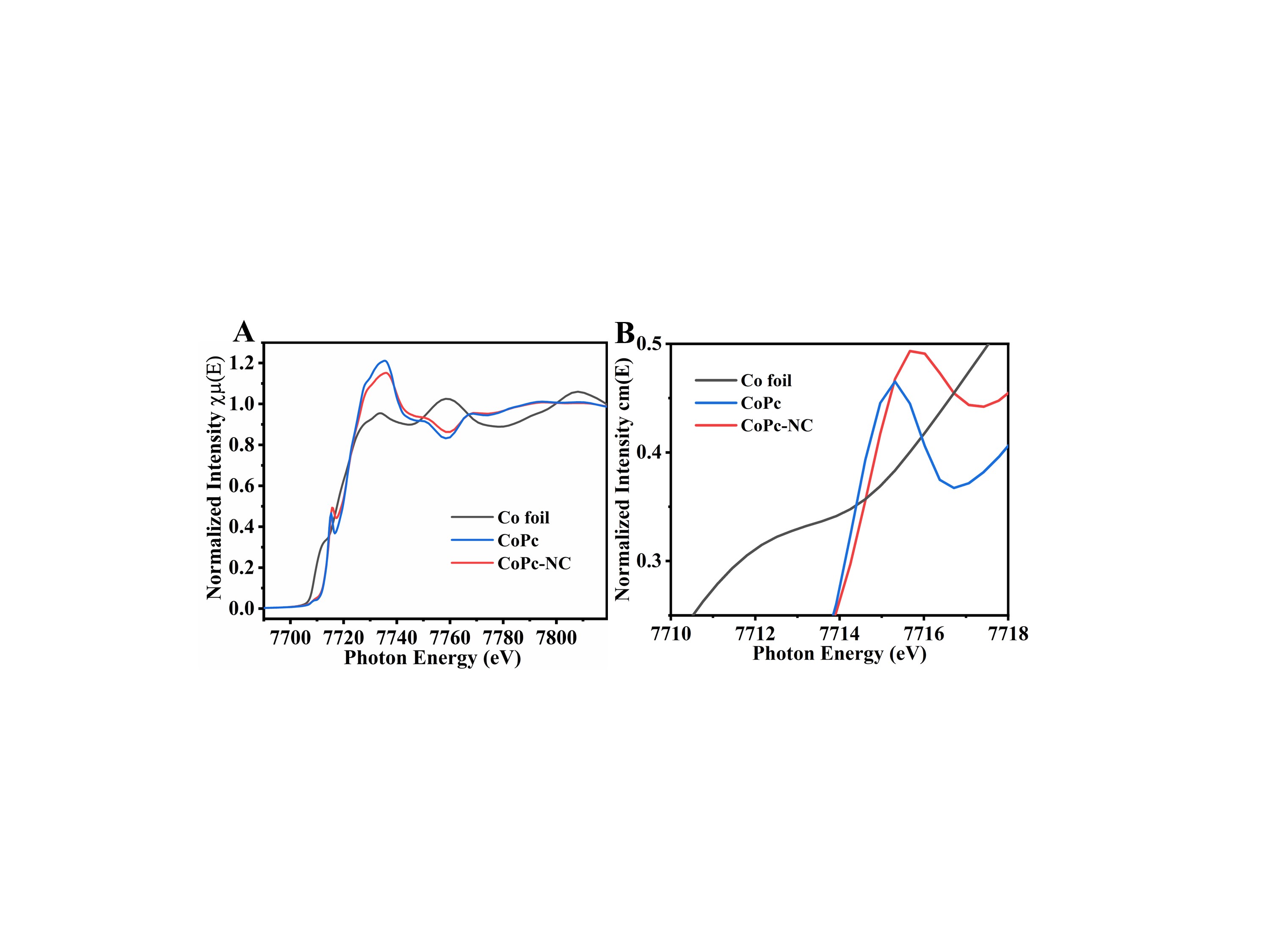
**

**Fig. S25** Co K-edge XANES spectra of CoPc-NC, CoPc, and Co foil


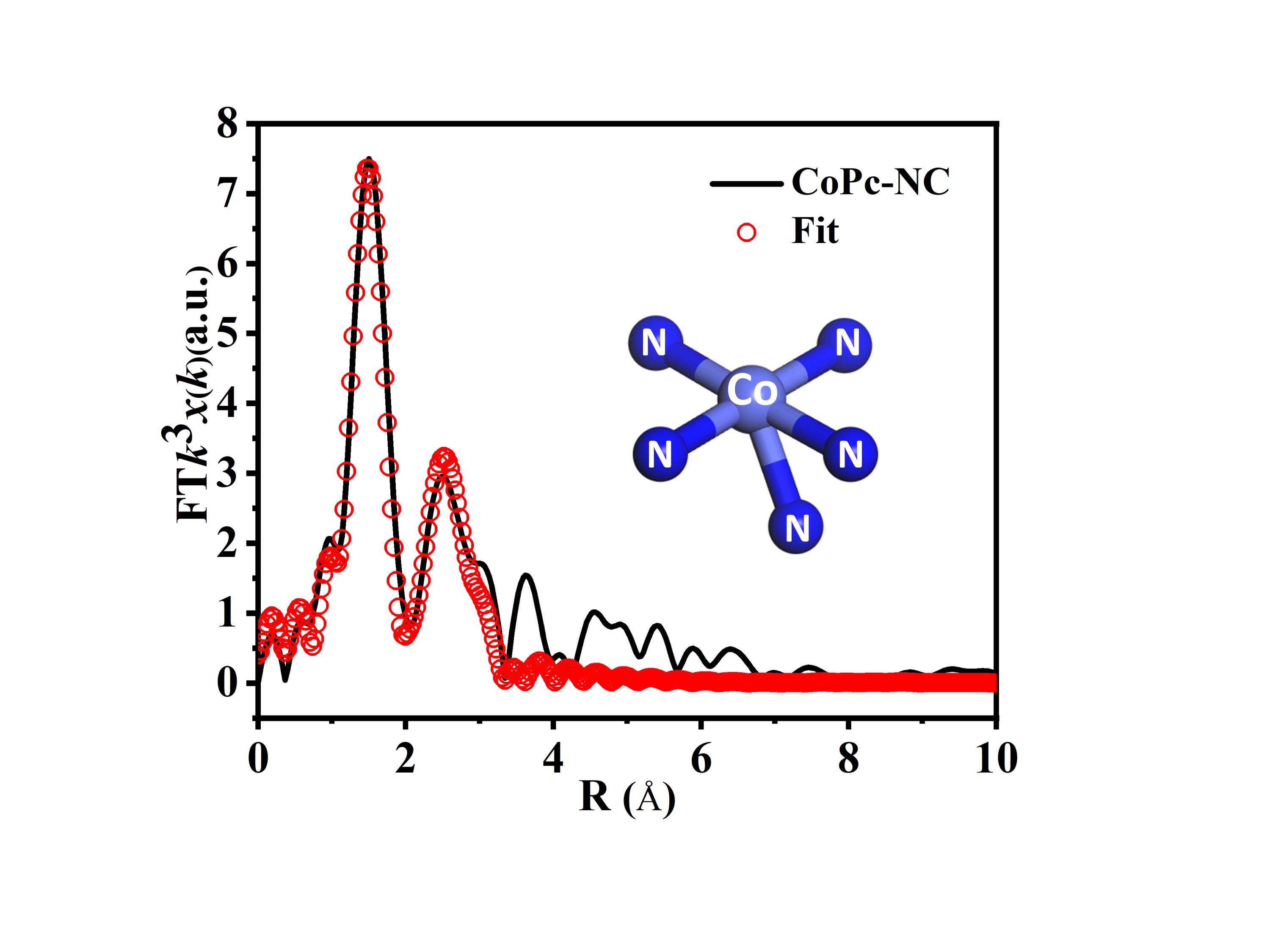


**Fig. S26** FT-EXAFS fitting curve and (inset) corresponding structure model of CoPc-NC


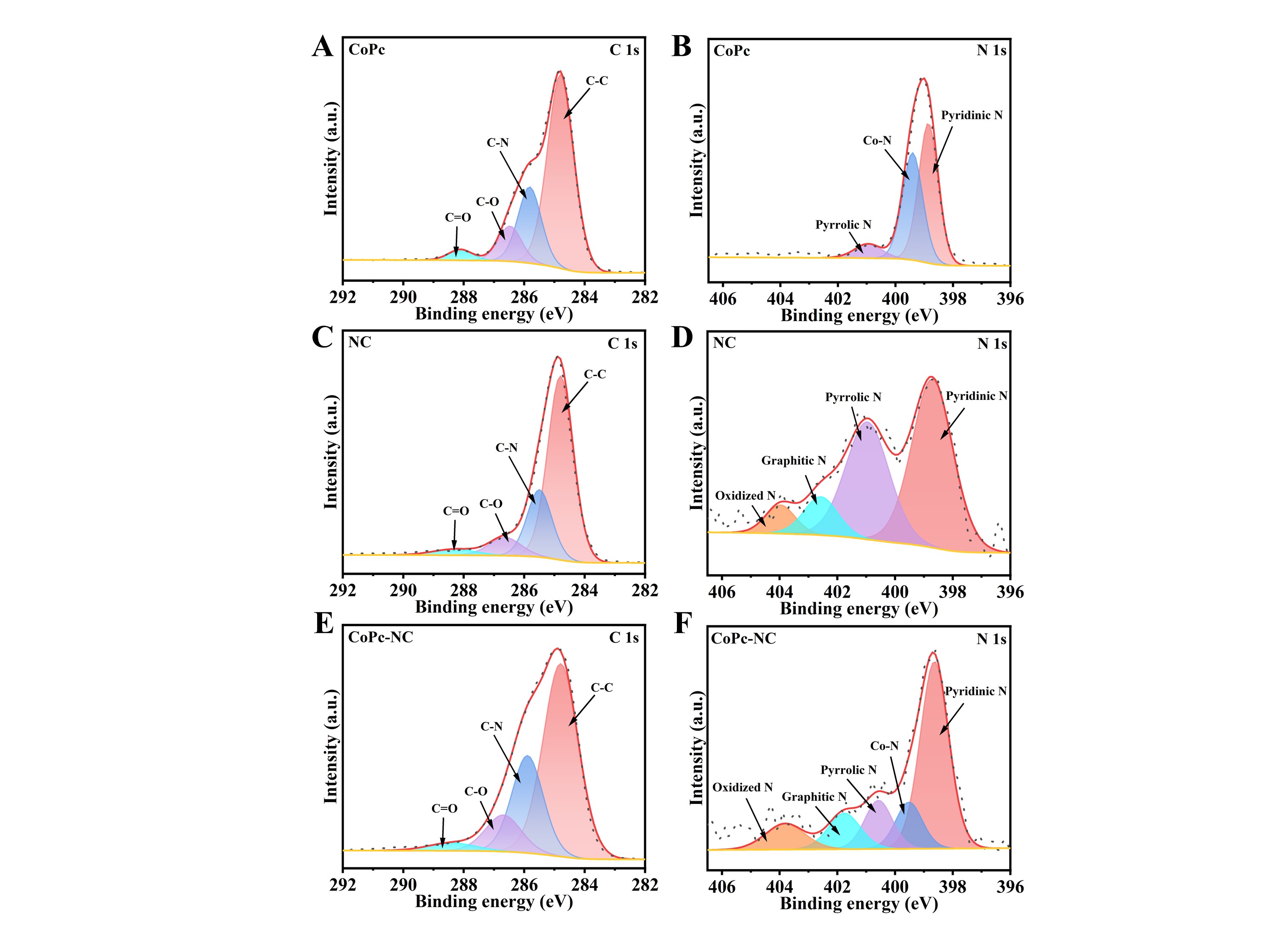


**Fig. S27** XPS high-resolution (**A, C,** and **E**) C 1s and (**B, D**, and **F**) N 1s spectra of CoPc, NC, and CoPc-NC, respectively

**Additional discussion**

As shown in Fig. S27, the XPS high-resolution C 1s spectra of CoPc, NC, and CoPc-NC could be divided into four peaks at 284.8, 285.7, 286.5, and 288.3 eV, which are related to C-C, C-N, C-O, and C=O, respectively. The N 1s spectra of CoPc, NC, and CoPc-NC display that N mainly existed as pyridinic N (398.6 eV), Co-N (399.2 eV), pyrrolic N (400.7 eV), graphitic N (401.9 eV), and oxided N (403.8 eV). Note that the peak intensity of pyrrolic N for CoPc-NC has evently decreased compared to NC, this may be caused by the coordination of pyrrolic N in NC substrate with Co single atom. And the strong coordination ability of Co atom with pyrrolic N is higher than that of other N species was reported by Li et al. [S5].


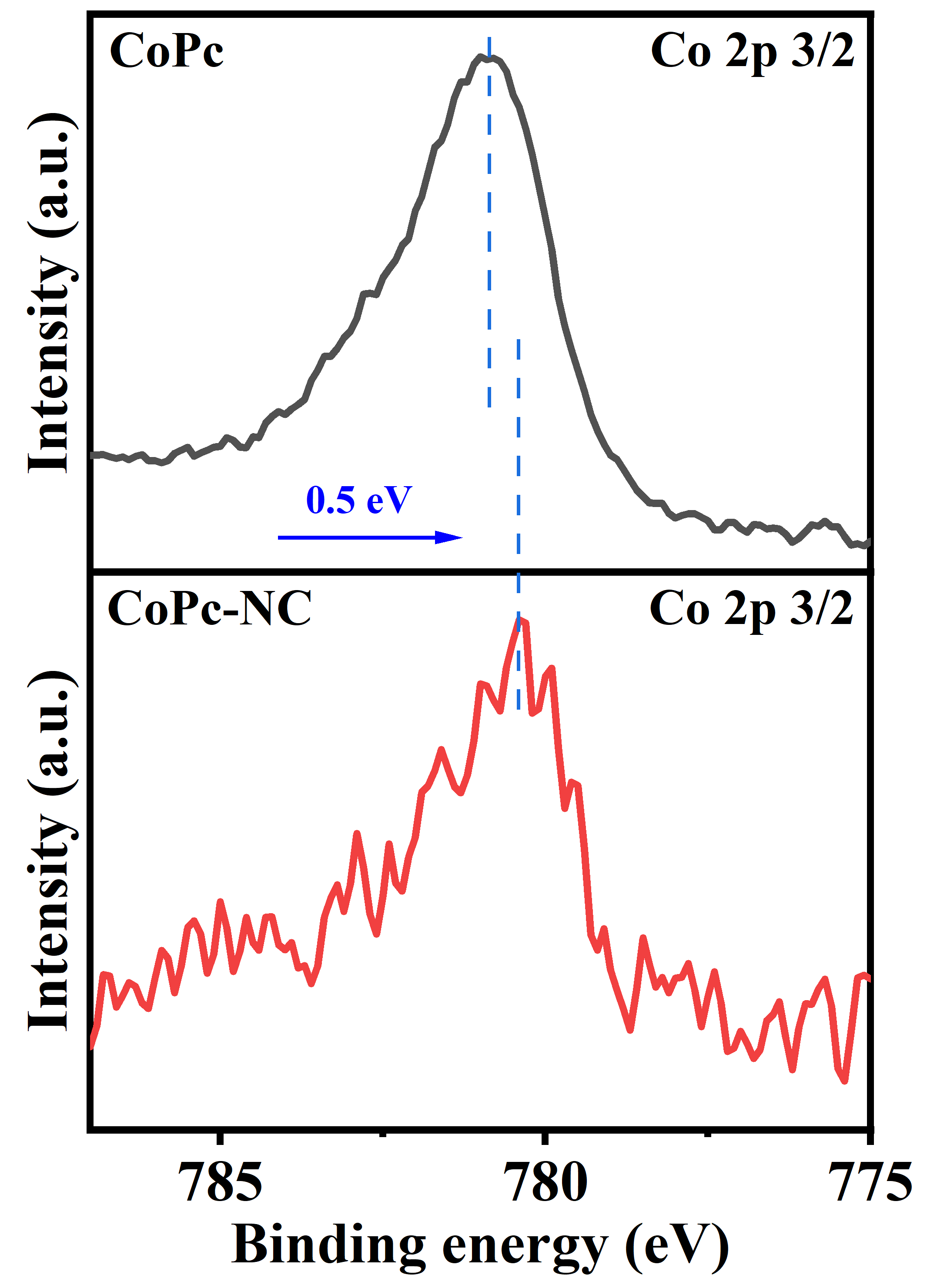


**Fig. S28** XPS high-resolution Co 2p spectra of CoPc and CoPc-NC


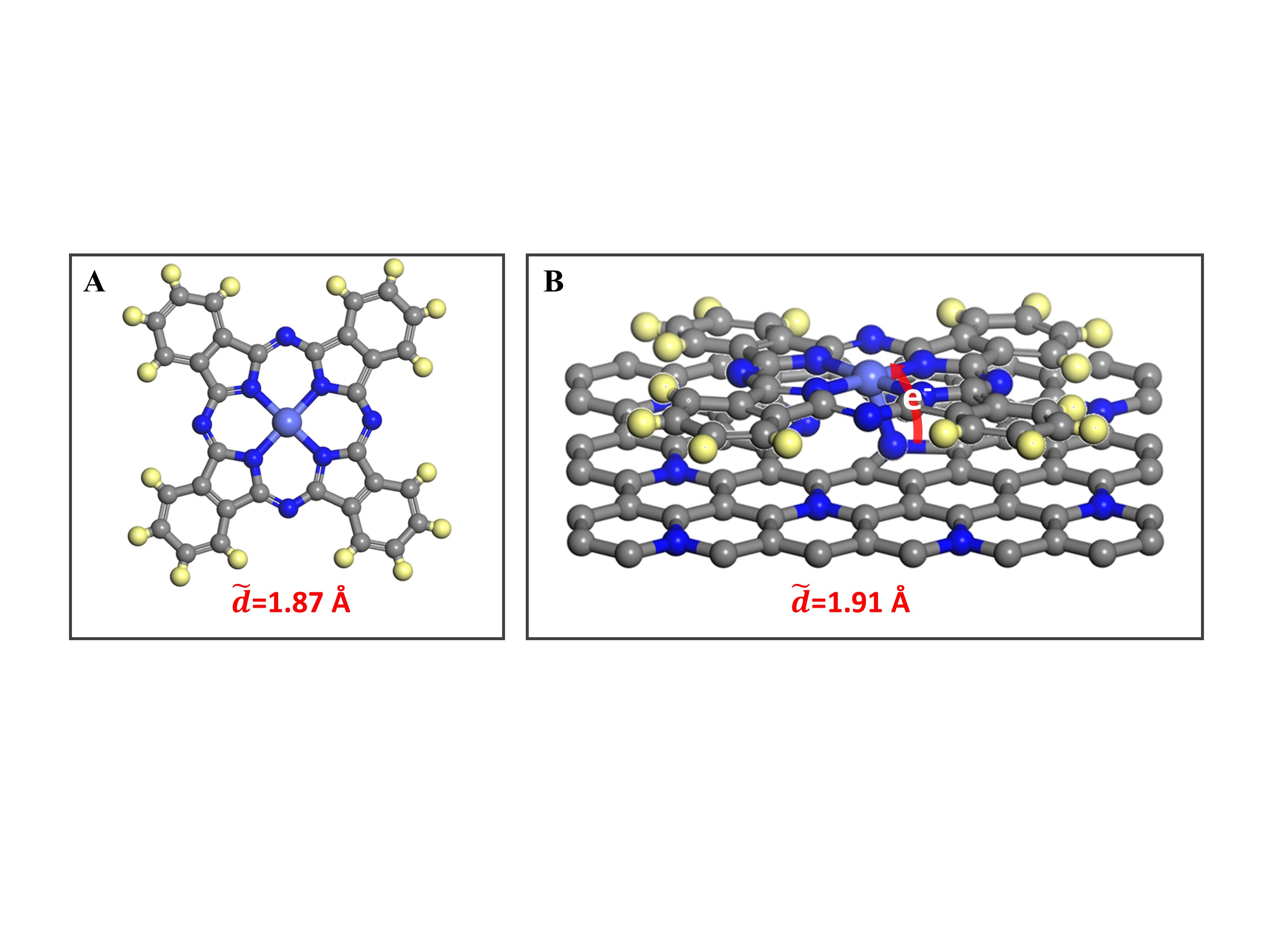


**Fig. S29** Diagram of (**A**) CoPc and (**B**) CoPc-NC, Co (wathet), N (blue), C (gray), H (yellow)


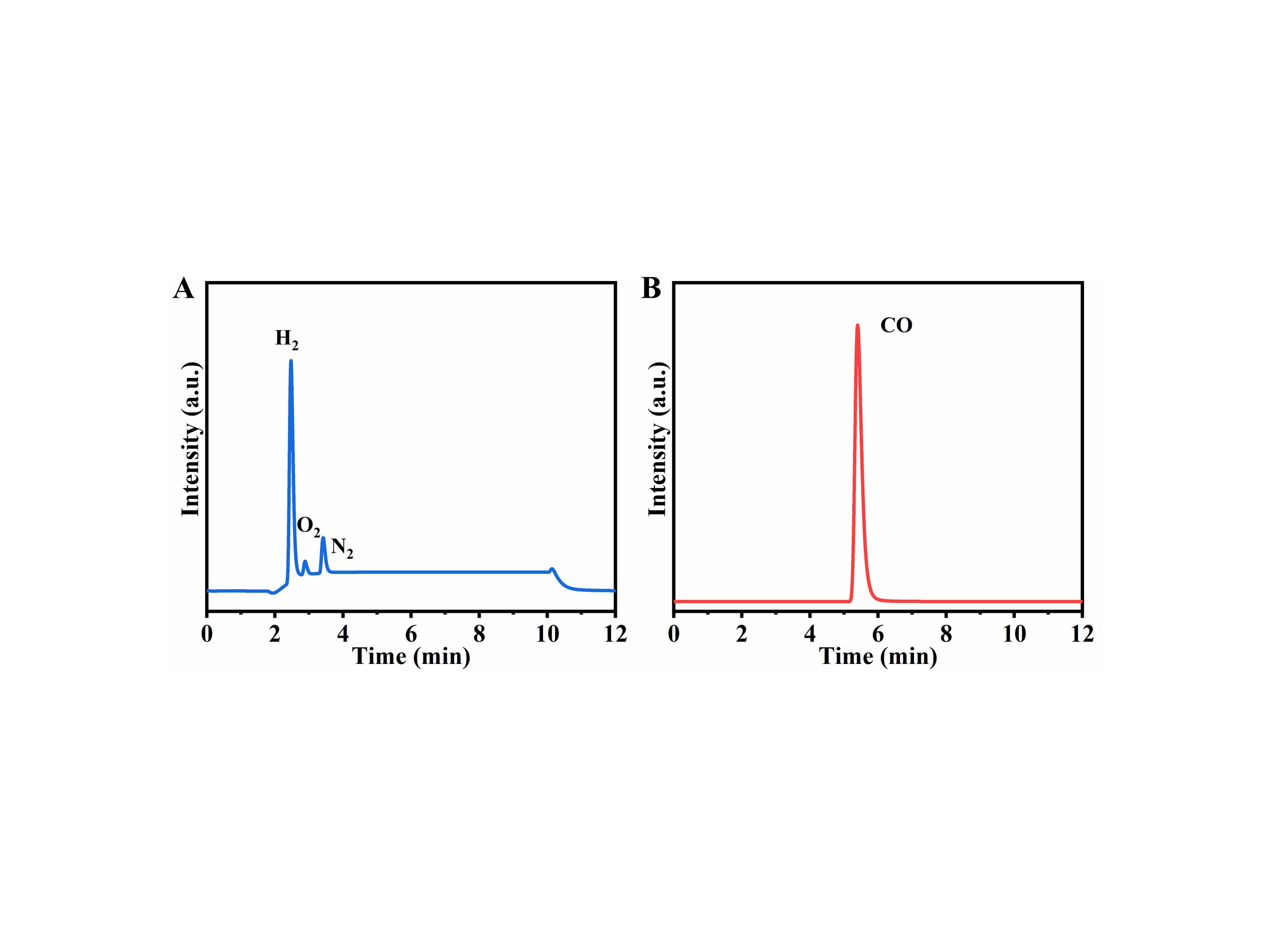


**Fig. S30** The gas chromatogram of gases produced by the cathode during PEC CO_2_RR at 1.23 V_RHE_

**Additional discussion**

The gas products in the cathode cell of the PEC CO_2_RR system was analyzed by a gas chromatograph (GC). A certain amount of gas was injected into the GC and was automatically entered two separate chromatographic columns for analysis. The columns equipped with TCD detector provide the accurate quantification of H_2_ (Fig. S30A), while the columns equipped with nickel reformers and FID detectors provide the accurate quantification of carbonaceous products (Fig. S30B).


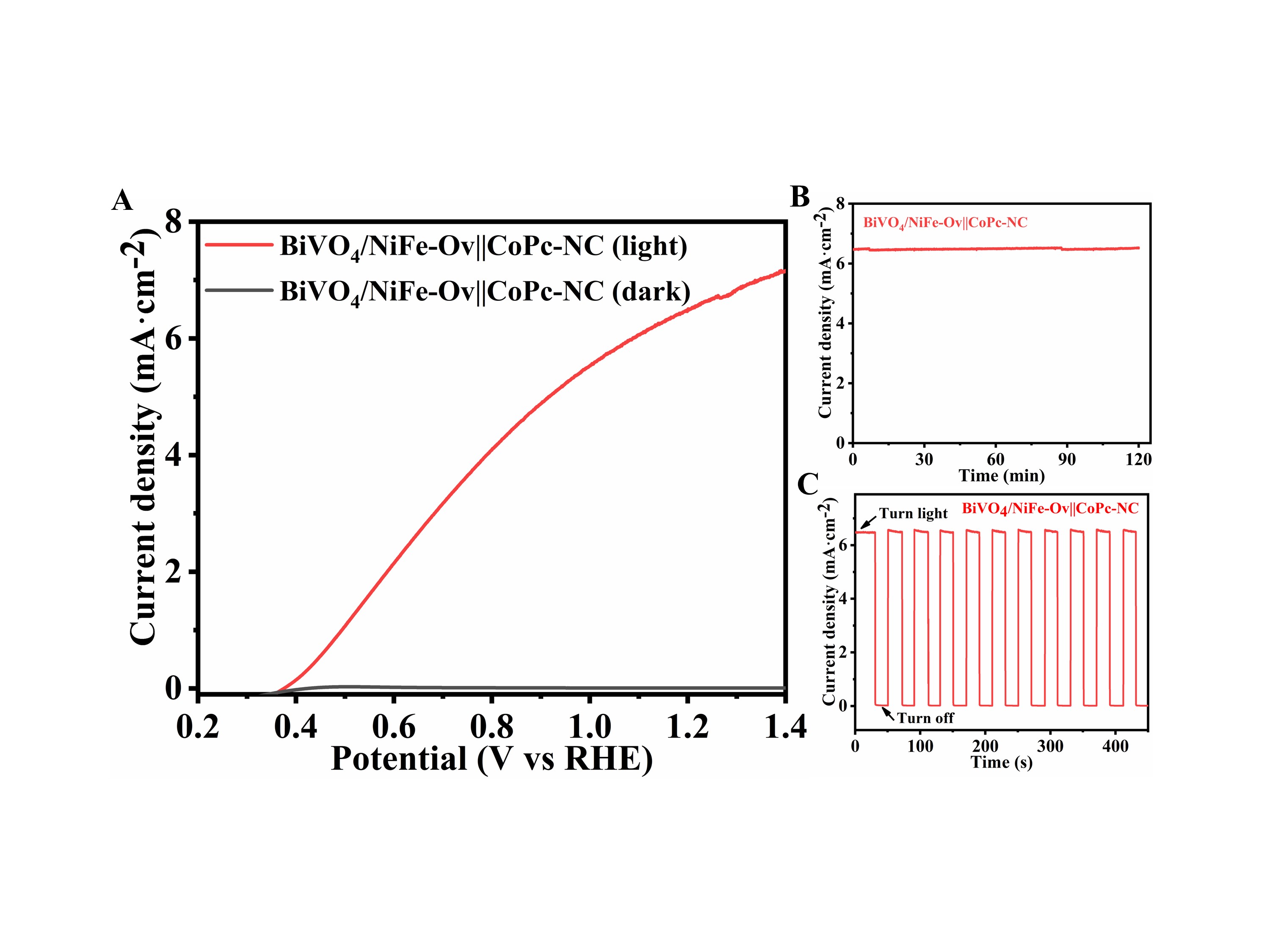


**Fig. S31** (**A**) LSV of BiVO_4_/NiFe-Ov‖CoPc-NC tandems for PEC CO_2_RR under illumination and dark; (**B**) I-t of BiVO_4_/NiFe-Ov‖CoPc-NC tandems for PEC CO_2_RR at 1.23 V_RHE_; (**C**) I-t curves of chopped irradiation on BiVO_4_/NiFe-Ov‖CoPc-NC tandems for PEC CO_2_RR at 1.23 V_RHE_


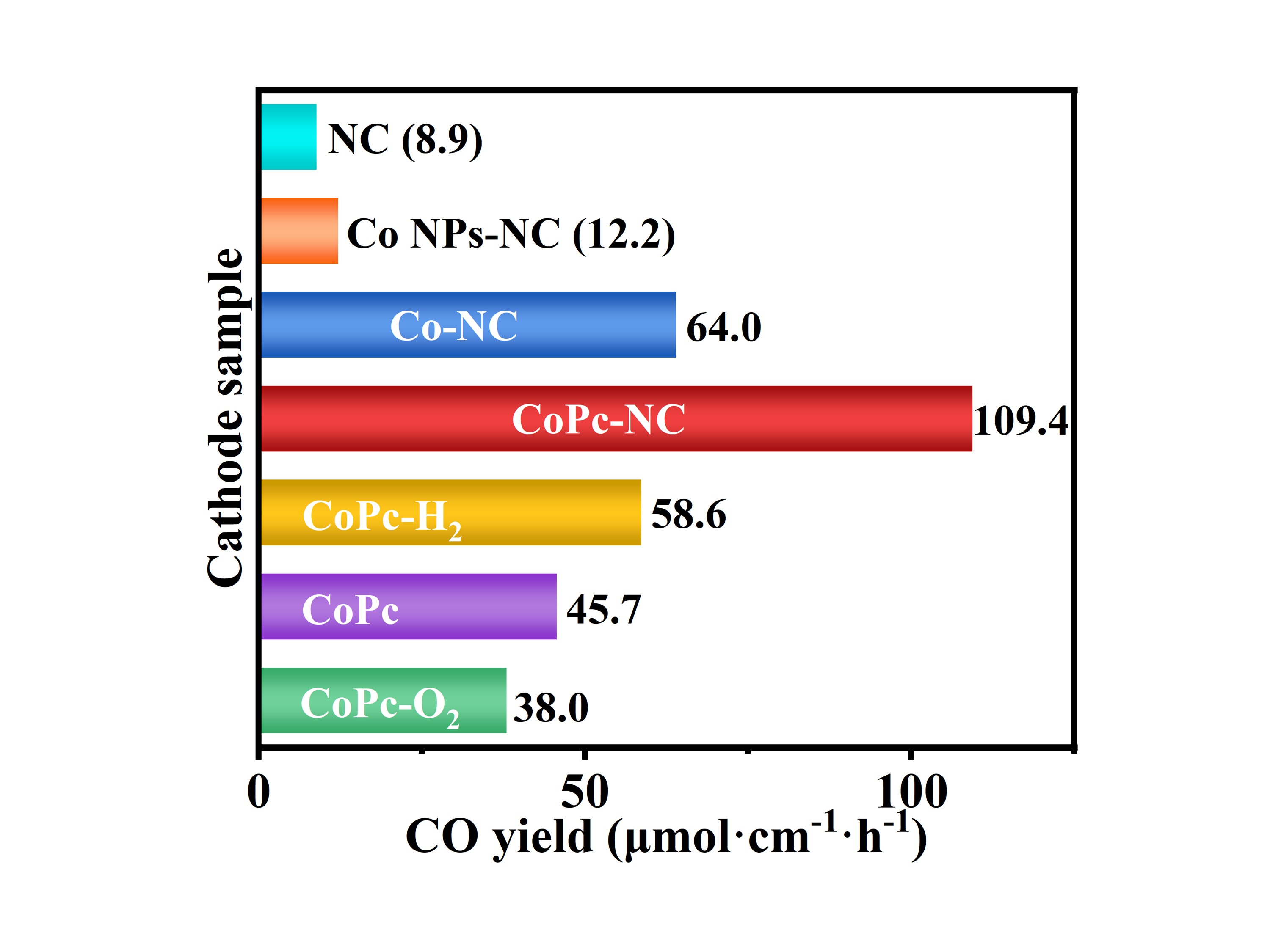


**Fig. S32** CO yield by integrating BiVO_4_/NiFe-Ov photoanode with different cathodes
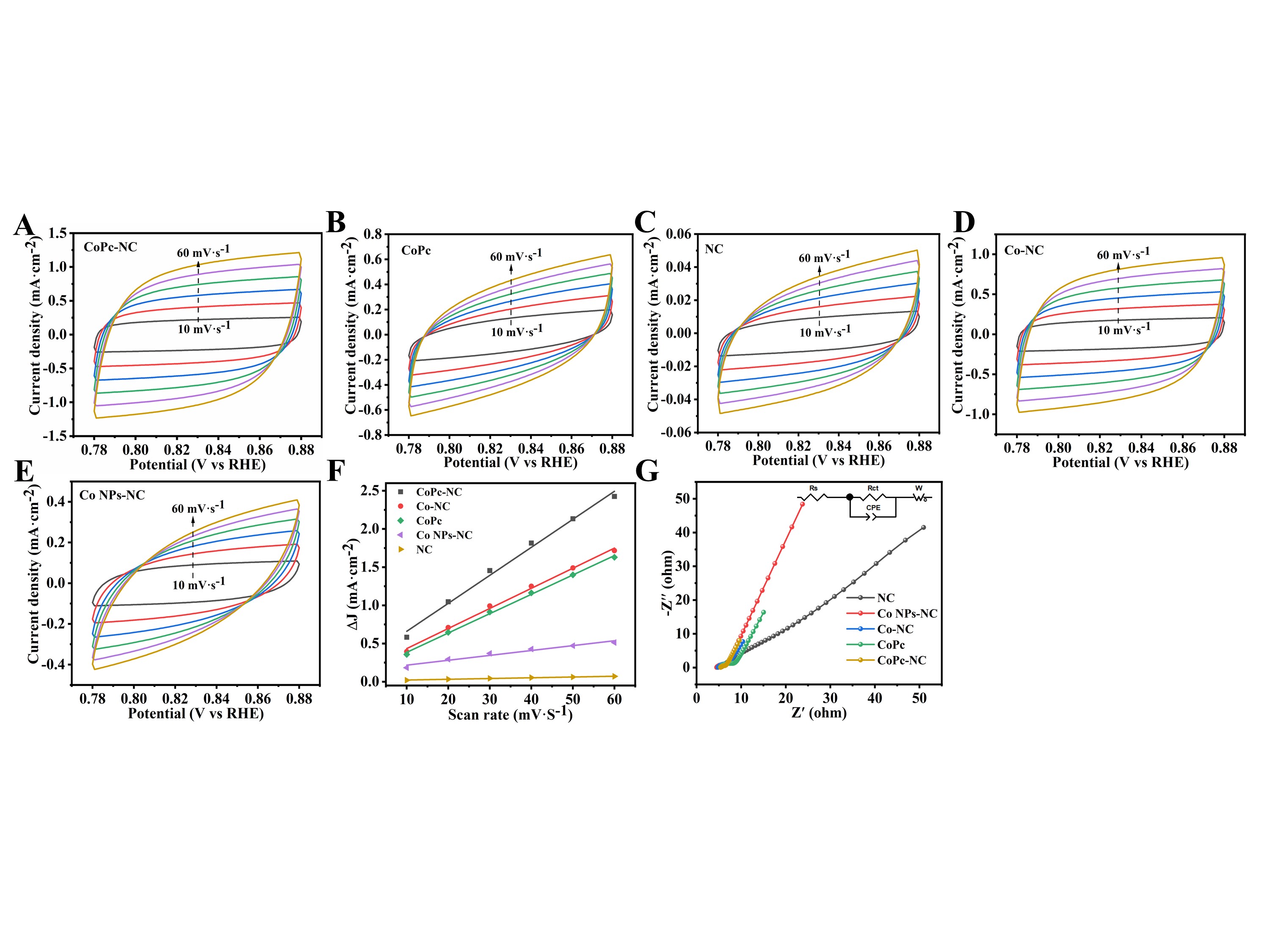


**Fig. S33** (**A-E**) CV curves, (**F**) the electrochemical double layer capacitance (C_dl_), and (G) the electrochemical impedance spectroscopy (EIS) plots of the CoPc-NC, CoPc, NC, Co-NC, and Co NPs-NC

**Additional discussion**

To explore the electrochemical active surface area, cyclic voltammetry curves have been performed at 0.83 V_RHE_ in 0.5 M KHCO_3_ under dark conditions. As shown in Fig. S33F, the electrochemical double layer capacitance (C_dl_) of CoPc-NC, CoPc, NC, Co-NC, and Co NPs-NC have been achieved to 21.7, 14.2, 0.7, 18.1, and 4.9 mF·cm^-2^, respecyively. Moreover, the electrochemical impedance spectroscopy (EIS) has been explored and shown in Fig. S33G, according to the Nyquist plots and the fitting results, the calculated resistance values of CoPc-NC, CoPc, NC, Co-NC, and Co NPs-NC are 4.5, 5.2, 5.5, 5.0, and 5.4 Ω, respectively.

**
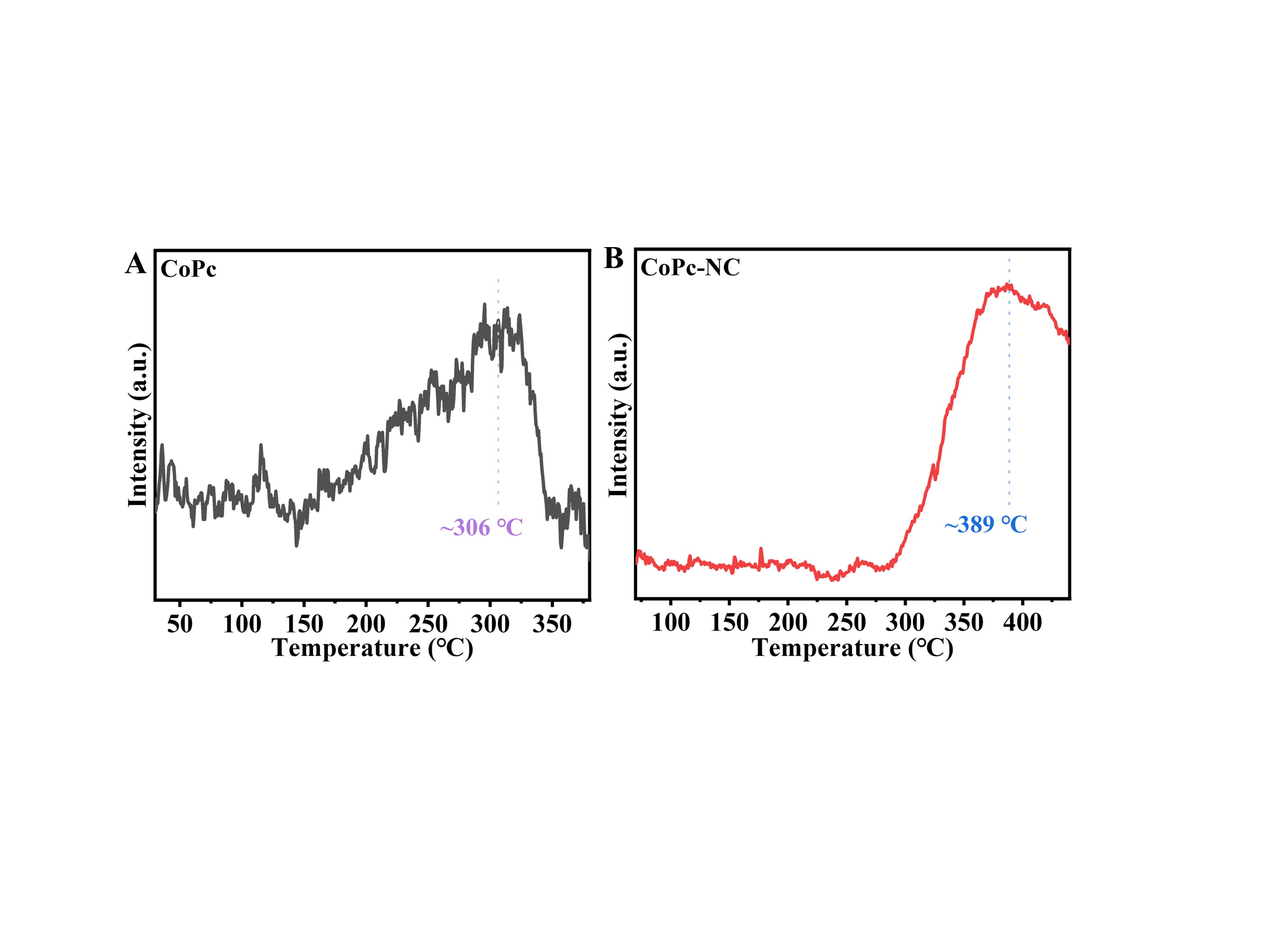
**

**Fig. S34** CO_2_-TPD curves of CO_2_ gas for (**A**) CoPc and (**B**) CoPc-NC


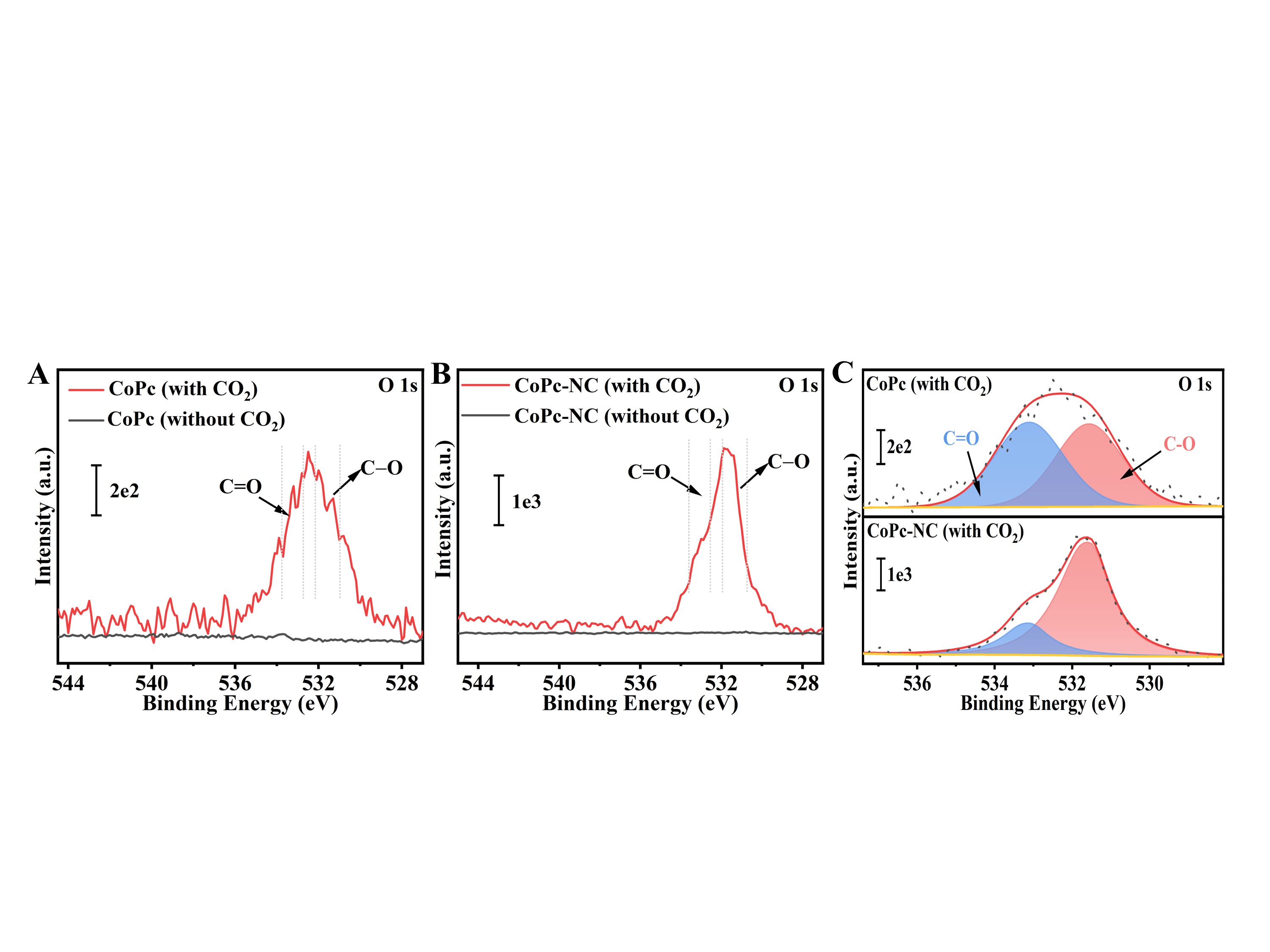


**Fig. S35** XPS high-resolution O 1s spectra of (**A**) CoPc and (**B**) CoPc-NC of before and after CO_2_ adsorption, and (**C**) O1s spectra comparion of CoPc and CoPc-NC after CO_2_ adsorption

**Additional discussion**

As shown in Fig. S35A and B, the CO_2_ adsorption capacity of CoPc-NC could be ~5 times that of pristine CoPc, which attributed to the high surface area of CoPc-NC and the excellent electronic regulation capabality between CoPc molecule and NC substrate.


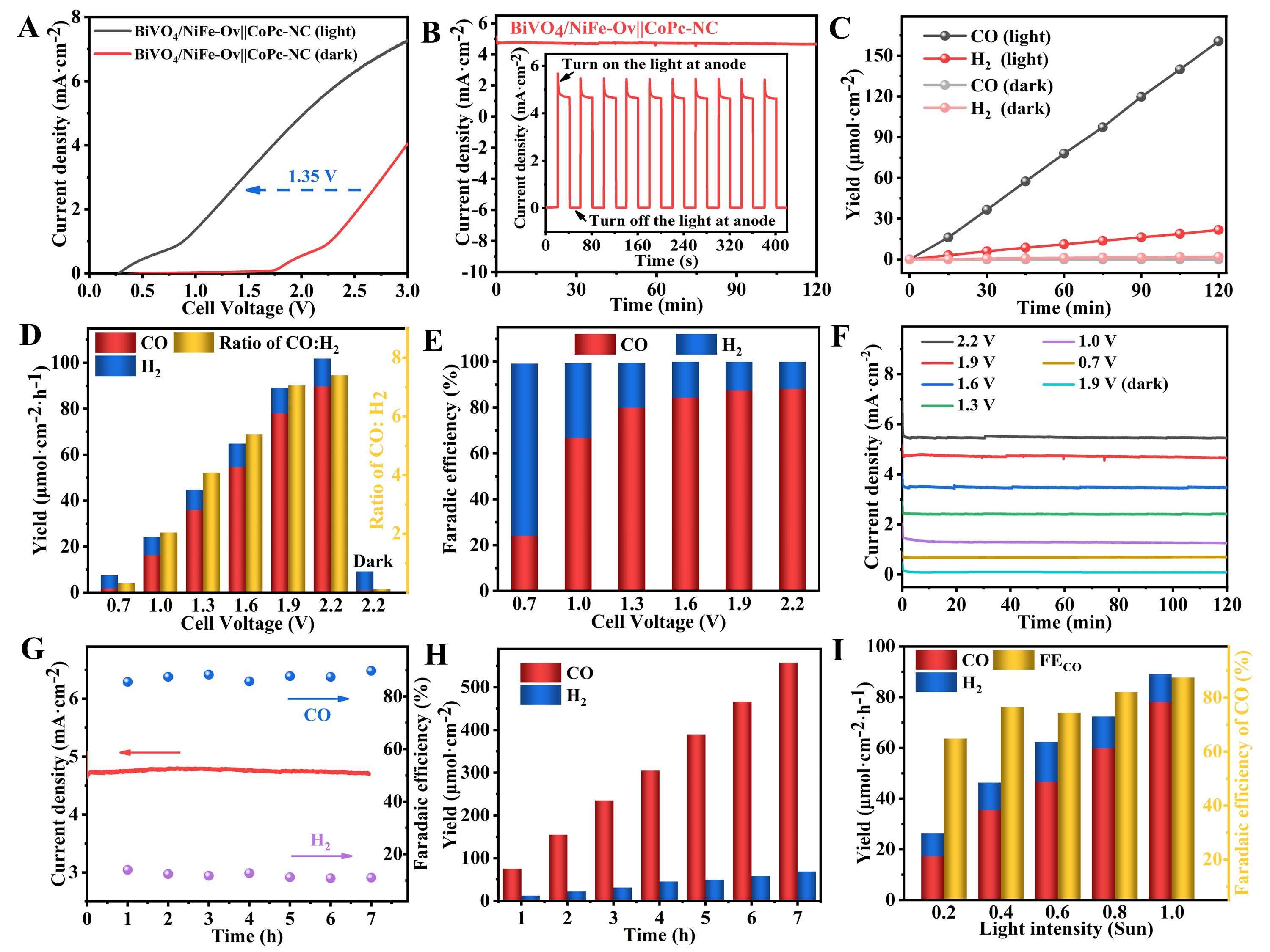


**Fig. S36** (**A**) LSV curves of BiVO_4_/NiFe-Ov‖CoPc-NC PEC CO_2_ reduction system under illumination and dark; (**B**) I-t curve of BiVO_4_/NiFe-Ov‖CoPc-NC PEC CO_2_ reduction system at 1.9 V; (**C**) CO/H_2_ yields of BiVO_4_/NiFe-Ov‖CoPc-NC PEC CO_2_ reduction system at 1.9 V; (**D**) CO/H_2_ yields and ratio of BiVO_4_/NiFe-Ov‖CoPc-NC PEC CO_2_ reduction system at different cell voltage; (**E**) Faradaic efficiency of CO and H_2_ over BiVO_4_/NiFe-Ov‖CoPc-NC PEC CO_2_ reduction system at different cell voltage; (**F**) I-t curve of BiVO_4_/NiFe-Ov‖CoPc-NC PEC CO_2_ reduction system at different cell voltage; (**G**) The stability experiment and (**H**) CO/H_2_ evolution amounts of BiVO_4_/NiFe-Ov‖CoPc-NC PEC CO_2_ reduction system during 7h test; (**I**) CO/H_2_ yields and FE_CO_ of BiVO_4_/NiFe-Ov‖CoPc-NC PEC CO_2_ reduction system under different light intensity at 1.9 V

**Additional discussion**

For the possibility of practical application in future, the performances of artificial photosynthetic CO_2_ reduction cells constructed by BiVO_4_/NiFe-Ov photoanode coupled CoPc-NC cathode have been conducted in a two-electrode system (Fig. S36). As shown in Fig. S36A, the required voltage for the PEC CO_2_ reduction system under illumination at photoanode could be lower by 1.35 V than that of the darkness, indicating that this PEC cell droved by BiVO_4_/NiFe-Ov photoanode is crucial for generating CO fuels at a low voltage. Besides, the amounts of CO and H_2_ produced by CoPc-NC cathode surface are 160.6 and 21.7 μmol·cm^-2^ after 2 h irradiation, respectively (Fig. S36C). In Fig. S36D, not only does the faradic efficiency of CO increase with enhancing cell voltage, but also that is greater than 80% at a wide cell voltage range of 1.3-2.2 V. During 7 h irradiation, an average CO faradic efficiency of 87.9% has been obtained on the PEC CO_2_ reduction system, as well as the amounts of CO and H_2_ exhibit a linear increasement (Fig. S36H), indicating its excellent selectivity and stability. Furthermore, the influences of light intensity on the activity of CO_2_ reduction have been investigated, which should be highly important for the practical applications (Fig. S36I). The CO faradaic efficiency of 64.9% has even been achieved for the PEC CO_2_ reduction system under the light intensity of 0.2 sun. This result suggests the PEC CO_2_ reduction system could be operated for CO production under sunny and cloudy weather.


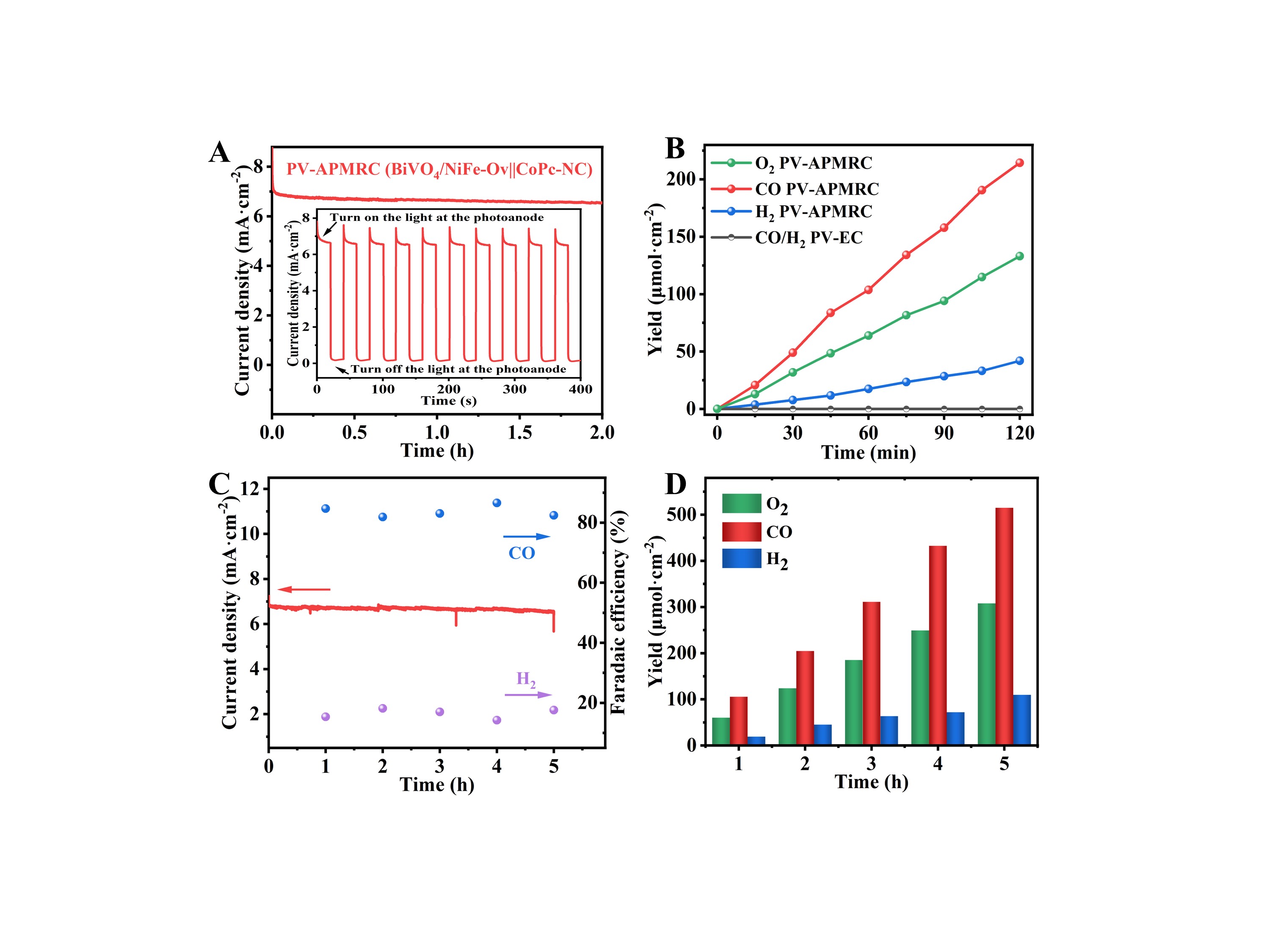


**Fig. S37** (**A**) I-t curve of BiVO_4_/NiFe-Ov‖CoPc-NC PV-APMRC; (**B**) O_2_, CO, and H_2_ yields of BiVO_4_/NiFe-Ov‖CoPc-NC PV-APMRC; (**C**) The stability experiment and (**D**) O_2_, CO, and H_2_ evolution amounts of BiVO_4_/NiFe-Ov‖CoPc-NC PV-APMRC

**Additional discussion**

For the possibility of practical application on Mars in future, replace the CO_2_ gas in the above PV-PEC CO_2_ reduction system with a simulated Mars atmosphere (95.51% CO_2_, 2.71% N_2_, 1.58% Ar, 0.132% O_2_, 0.0699% CO), noted as PV-APMRC system. When a commercial PV cell with an output voltage of 2 V was applied to assemble with the APMRC, the photocurrent density could be reached to 6.7 mA·cm^-2^ under light intensity of 2 sun (Fig. S37A). However, the photocurrent density of PV-APMRC system was rapidly dropped to near zero, and no gas was detected (Fig. S37A inset), confirming that the PV-APMRC system for oxygen and syngas production is driven by BiVO_4_/NiFe-Ov photoanode rather than solar cell. In Fig. S37B, the amounts of O_2_, CO and H_2_ reached 63.8, 103.6 and 17.5 μmol·cm^-2^ after 1 h irradiation, respectively, exhibiting the appropriate stoichiometric ratio of ~2:1 (CO+H_2_/O_2_). As shown in Fig. S37C, the faradaic efficiency of CO and H_2_ could be constant during the 5h irradiation, suggesting the excellent stability of the PV-APMRC system. After 5h irradiation, the amounts of O_2_, CO and H_2_ could be reached up to 9.9, 14.4, and 0.22 mg·cm^-2^, respectively (Fig. S37D).

**S3 Supplemental Tables and Discussions**

**Table S1** Fitting results of the Nyquist plots of BiVO_4_ and BiVO_4_/NiFe-Ov photoanodes

|  | **Rct (Ω)** | **CPE (F·cm^-2^)** |
| --- | --- | --- |
| **BiVO_4_** | 665.1 | 3.8×10^-4^ |
| **BiVO_4_/NiFe-Ov** | 76.1 | 4.2×10^-4^ |

**Table S2** Fitting parameters of transient absorption kinetics of BiVO_4_ and BiVO_4_/NiFe-Ov photoanodes

|  | **τ_1_ (ns)** | **τ_2_ (ns)** | **τ_ave_ (ns)** |
| --- | --- | --- | --- |
| **BiVO_4_** | 1747.56 (80.39%) | 83.28 (19.61%) | 1420.95 |
| **BiVO_4_/NiFe-Ov** | 3585.65 (94.38%) | 105.34 (5.62%) | 3390.06 |

**Table S3** ICP-OES results of Ni and Fe content in BiVO_4_/NiFe-Ov photoanodes

| **Element** | **Volume (mL)** | **Concentration (mg/L)** | **Calculated content (wt%)** |
| --- | --- | --- | --- |
| **Ni** | 1 | 0.0925 | 0.093 |
| **Fe** | 1 | 0.9222 | 0.922 |

**Additional discussion**

The loading amounts of Ni and Fe in BiVO_4_/NiFe-Ov photoanodes have been analyzed by an inductively coupled plasma optical emission spectroscopy (ICP-OES, Agilent 700). As shown in Table S3, the loading amounts of Ni and Fe in BiVO_4_/NiFe-Ov were calculated to be 0.093 wt%, and 0.92 wt%, respectively, and the ratio of Ni and Fe was calculated to be about 0.1.

**Table S4** ICP-MS results of leaking content for BiVO_4_/NiFe-Ov photoanodes after the stability test

| **Element** | **Volume (mL)** | **Concentration (mg/L)** | **Calculated amount (μmol/L)** |
| --- | --- | --- | --- |
| **Ni** | 1 | 0.001 | 0.00002 |
| **Fe** | 1 | 0.0193 | 0.00035 |

**Additional discussion**

The leaking amounts of BiVO_4_/NiFe-Ov photoanodes after the stability test have been analyzed by an inductively coupled plasma mass spectroscopy (ICP-MS, Agilent 7800). The contents of Ni, and Fe after the PEC stability test were calculated to be 0.00002, and 0.00035 µmol/L, respectively, indicating no evident dissolution of Ni and Fe during the stability test (Table S4).

**Table S5** EXAFS fitting parameters at the Co K-edge for CoPc-NC sample

|  | **Shell** | ***CN****^a^* | ***R*(Å)***^b^* | ***σ*^2^×10^3^(Å^2^)***^c^* | **Δ*E*_0_ (eV)***^d^* | ***R* factor** |
| --- | --- | --- | --- | --- | --- | --- |
| **CoPc-NC** | Co-N | 4.7±0.3 | 1.91±0.01 | 3.5 | 7.7±1.8 | 0.0086 |

*^a^CN*: coordination numbers; *^b^R*: bond distance; *^c^σ*^2^: Debye-Waller factors; *^d^* Δ*E*_0_: the inner potential correction. *R* factor: goodness of fit.

**Additional discussion**

The obtained XAFS data was processed in Athena (version 0.9.26) for background, pre-edge line and post-edge line calibrations. Then Fourier transformed fitting was carried out in Artemis (version 0.9.26). The k^3^ weighting, k-range of 3-14 Å^-1^ and R range of 1-3 Å were used for the fitting of Co foil; k-range of 3-11 Å^-1^ and R range of 1-3 Å were used for the fitting of samples. The four parameters, coordination number, bond length, Debye-Waller factor and E_0_ shift (CN, R, σ^2^, ΔE_0_) were fitted without anyone was fixed, the σ^2^ was set. For Wavelet Transform analysis, the χ(k) exported from Athena was imported into the Hama Fortran code. The parameters were listed as follow: R range, 1-4 Å, k range, 0-12 Å^-1^ for samples; k weight, 3; and Morlet function with κ=10, σ=1 was used as the mother wavelet to provide the overall distribution.

**Table S6** XPS analysis results of CoPc, NC, and CoPc-NC samples (unit: at%)

|  | **C** | **N** | **Co** |
| --- | --- | --- | --- |
| **CoPc** | 83.59 | 14.87 | 1.55 |
| **NC** | 96.38 | 3.63 | - |
| **CoPc-NC** | 96.23 | 3.52 | 0.17 |

**Table S7** XPS analysis results of different N species in CoPc, NC, and CoPc-NC samples (unit: at%)

|  | **Pyridinic N** | **Co-N** | **Pyrrolic N** | **Graphitic N** | **Oxidized N** | **Ratio of Co-N/Co** |
| --- | --- | --- | --- | --- | --- | --- |
| **CoPc** | 7.64 | 6.13 | 1.10 | - | - | 3.96 |
| **NC** | 1.77 | - | 1.31 | 0.22 | 0.33 | - |
| **CoPc-NC** | 1.25 | 0.91 | 0.50 | 0.32 | 0.54 | 5.35 |

**Additional discussion**

The XPS has been used to investigate the chemical composition and electron structure. The different N species and content of CoPc, NC, and CoPc-NC samples could be analyzed and shown in Table S6 and Table S7, the ratio of Co-N to Co is close to 5. This result further confirms that the coordination detail of Co single atom in CoPc-NC should be Co-N_5_, which is agreement with the X-ray absorption structure spectroscopy result.

**Table S8** ICP-OES measurement results of Co content in CoPc-NC sample

| **Elements wavelength (nm)** | **Concentration (mg/L)** | **RSD (%)** | **Calculated content (wt%)** |
| --- | --- | --- | --- |
| Co 228.615 | 1.12655 | 6.9 | 2.25310 |
| Co 231.406 | 1.07329 | 8.1 | 2.14658 |
| Co 236.379 | 1.14333 | 7.0 | 2.28666 |
| Co 237.863 | 1.13440 | 7.1 | 2.26880 |
| Co 238.345 | 1.11102 | 9.7 | 2.22204 |

**Additional discussion**

The loading amounts of cobalt in CoPc-NC sample have been analyzed by an inductively coupled plasma optical emission spectroscopy (ICP-OES, Agilent 725-ES). As shown in Table S8, the average content of cobalt in CoPc-NC was calculated to be 2.24 wt%.

**Table S9** Summary of recent significant progress in BiVO_4_-based photoanodes

| **Photoanodes** | **Photocurrent (mA·cm^-2^ 1.23 V_RHE_)** | **Year** | **Refs.** |
| --- | --- | --- | --- |
| BiVO_4_/FeOOH/NiOOH | 4.5 | 2014 | [S6] |
| WO_3_/BiVO_4_/CoPi | 6.72 | 2015 | [S7] |
| Mo:BiVO_4_/Fe(Ni)OOH | 5.82 | 2016 | [S8] |
| Ni:FeOOH/WO_3_/BiVO_4_ | 4.5 | 2016 | [S9] |
| NiOOH/FeOOH/CQD/BiVO_4_ | 5.99 | 2017 | [S10] |
| FeCoO_x_/BiVO_4_ | 4.82 | 2018 | [S11] |
| Mo:BiVO_4_@TANF | 5.1 | 2018 | [S12] |
| BiVO_4_/TiO_2-x_ | 6.12 | 2019 | [S13] |
| NiFeO_x_/BiVO_4_ | 5.54 | 2020 | [S14] |
| BiVO_4_/Fe_x_Ni_1-x_OOH | 5.8 | 2020 | [S15] |
| Ov-BiVO_4_@NiFe-MOFs | 5.3 | 2021 | [S16] |
| BiVO_4_/N:NiFeO_x_ | 6.4 | 2021 | [S17] |
| BiVO_4_/Bi/NiFeOOH | 4.7 | 2022 | [S18] |
| BiVO_4_/NiFePO_x_ | 6.73 | 2022 | [S19] |
| NiOOH/Co_3_O_4_/BiVO_4_ | 6.4 | 2023 | [S20] |
| NiFe/CA/Fe-BiVO_4_ | 6.2 | 2024 | [S21] |
| BiVO_4_/NiFe-Ov | 6.51 | This work |  |

**Table S10** The performance of photoanode-driven PEC CO_2_ reduction

| **Photoanode** | **Cathode** | **Products and FE^a^/%** | **Yield/μmol cm^-2^ h^-1^** | **Refs.** |
| --- | --- | --- | --- | --- |
| TiO_2_ NR | Cu_2_O | CH_4_ (54.63)  CO (30.03)  CH_3_OH (2.79) | 3.4  7.5  0.2 | [S22] |
| WO_3_ | Sn/SnO_x_ | CO (17.5)  HCOOH (26.8) | 12.9  19.8 | [S23] |
| WO_3_ | Cu | CH_4_ (67)  C_2_H_4_ (2.7) | 2.8  0.1 | [S24] |
| CNTs/ZnO/Co_3_O_4_ | Pd-Cu | CO (75) | 5.6 | [S25] |
| Si-Ni | Ag | CO (90) | - | [S26] |
| Si-Ni | Ag^b^ | CO (70) | - | [S27] |
| Fe_2_O_3_/Co-C_3_N_4_ | Ag-Pd | CO (51.4) | 42.1 | [S28] |
| RuO_x_-Al_2_O_3_-Fe_2_O_3_ | Ni-SNG | CO (90) | 26.5 | [S29] |
| Co-Ci/BiVO_4_/WO_3_ | Cu | CO (1.4)  C_2_H_4_ (3.7)  CH_4_ (46.8) | 0.24  0.11  2.03 | [S30] |
| Co-Pi/BiVO_4_ | Au | CO (90) | - | [S31] |
| NiFe-BiVO_4_ | AgO_x_/Ag | CO (66.9) | 84.7 | [S32] |
| BiVO_4_/NiFe-Ov | CoPc-NC | CO (90.6) | 109.4 | This work |

a: The faradaic efficiency of products. b: Nanoporous Ag.

**Table S11** Comparisons of solar energy conversion efficiency for unbiased-PEC CO_2_ reduction

| **(Photo)anode** | **(Photo)cathode** | **PV** | **η (STF)** | **η (STC)** | **Refs.** |
| --- | --- | --- | --- | --- | --- |
| Si-Ni foam | Cu-Zn | Si | 4.3% | 0.8% | [S33] |
| Si-Ni | Ag | Si | --- | 2.42% | [S26] |
| Pt | Cu-Sn | DDSC^a^ | 0.97% | 0.79% | [S34] |
| Pt | CuFeO_2_/CuO | Si | 1.2% | 1% | [S35] |
| BiVO_4_/TiCo | CoMTPP@CNT | Perovskite | 0.633% | 0.053% | [S36] |
| Co-Ci | Au/CdTe/ZnTe | Perovskite | 0.43% | 0.35% | [S37] |
| BiVO_4_/Co | CoMTPP@CNT | Perovskite | 0.08% | 0.02% | [S38] |
| BiVO_4_ | Cu_96_In_4_ | Perovskite | 0.25% | 0.19% | [S39] |
| Mo:BiVO_4_/Co-Pi | CuAg/GDE | CuInSe | 2% | 0.56% | [S40] |
| BiVO_4_/NiFe-Ov | CoPc-NC | Si | 5.41% | 4.44% | This work |

a: dye-sensitized solar cell.

**Supplementary References**

1. Y. Wang, D. Chen, J. Zhang, M.-S. Balogun, P. Wang et al., Charge relays via dual carbon-actions on nanostructured BiVO_4_ for high performance photoelectrochemical water splitting. Adv. Funct. Mater. **32**, 2112738 (2022). <https://doi.org/10.1002/adfm.202112738>
2. A. Kudo, K. Omori, H. Kato, A novel aqueous process for preparation of crystal form-controlled and highly crystalline BiVO_4_ powder from layered vanadates at room temperature and its photocatalytic and photophysical properties. J. Am. Chem. Soc. **121**, 11459-11467 (1999). <https://doi.org/10.1021/ja992541y>
3. S. Gu, W. Li, F. Wang, S. Wang, H. Zhou et al., Synthesis of buckhorn-like BiVO_4_ with a shell of CeO_x_ nanodots: Effect of heterojunction structure on the enhancement of photocatalytic activity. Appl. Catal. B-Environ. **170**, 186-194 (2015). <https://doi.org/10.1016/j.apcatb.2015.01.044>
4. J. Gao, Y. Hu, Y. Wang, X. Lin, K. Hu et al., MOF structure engineering to synthesize Co-N-C catalyst with richer accessible active sites for enhanced oxygen reduction. Small **17**, 2104684 (2021). <https://doi.org/10.1002/smll.202104684>
5. Y. Pan, R. Lin, Y. Chen, S. Liu, W. Zhu et al., Design of single-atom Co-N_5_ catalytic site: a robust electrocatalyst for CO_2_ reduction with nearly 100% CO selectivity and remarkable stability. J. Am. Chem. Soc. **140**, 4218 (2018). <https://doi.org/10.1021/jacs.8b00814>
6. T. W. Kim, K.-S. Choi, Nanoporous BiVO_4_ photoanodes with dual-layer oxygen evolution catalysts for solar water splitting. Science 343, 990 (2014). <https://doi.org/10.1126/science.1246913>
7. Y. Pihosh, I. Turkevych, K. Mawatari, J. Uemura, Y. Kazoe et al., Photocatalytic generation of hydrogen by core-shell WO_3_/BiVO_4_ nanorods with ultimate water splitting efficiency. Sci. Rep. **5**, 11141 (2015). <https://doi.org/10.1038/srep11141>
8. Y. Qiu, W. Liu, W. Chen, W. Chen, G. Zhou et al., Efficient solar-driven water splitting by nanocone BiVO_4_-perovskite tandem cells. Sci. Adv. **2**, e1501764 (2016). <https://doi.org/10.1126/sciadv.1501764>
9. L. Cai, Ji. Zhao, H. Li, J. Park, I.S. Cho et al., One-step hydrothermal deposition of Ni:FeOOH onto photoanodes for enhanced water oxidation. ACS Energy Lett. **1**, 624-632 (2016). <https://doi.org/10.1021/acsenergylett.6b00303>
10. K.-H.Ye, Z. Wang, J. Gu, S. Xiao, Y. Yuan et al., Carbon quantum dots as a visible light sensitizer to significantly increase the solar water splitting performance of bismuth vanadate photoanodes. Energy Environ. Sci. **10**, 772-779 (2017). <https://doi.org/10.1039/C6EE03442J>
11. S. Wang, T. He, J.-H. Yun, Y. Hu, M. Xiao et al., New iron-cobalt oxide catalysts promoting BiVO_4_ films for photoelectrochemical water splitting. Adv. Funct. Mater. **28**, 1802685 (2018). <https://doi.org/10.1002/adfm.201802685>
12. Y. Shi, Y. Yu, Y. Yu, Y. Huang, B. Zhao et al., Boosting photoelectrochemical water oxidation activity and stability of Mo-doped BiVO_4_ through the uniform assembly coating of NiFe-phenolic networks. ACS Energy Lett. **3**, 1648-1654 (2018). <https://doi.org/10.1021/acsenergylett.8b00855>
13. Z. Tian, P. Zhang, P. Qin, D. Sun, S. Zhang et al., Novel black BiVO_4_/TiO_2−x_ photoanode with enhanced photon absorption and charge separation for efficient and stable solar water splitting. Adv. Energy Mater. **9**, 1901287 (2019). <https://doi.org/10.1002/aenm.201901287>
14. S. Wang, T. He, P. Chen, A. Du, K. Ostrikov, W. Huang et al., In situ formation of oxygen vacancies achieving near-complete charge separation in planar BiVO_4_ photoanodes. Adv. Mater. **32**, 2001385 (2020). <https://doi.org/10.1002/adma.202001385>
15. B. Zhang, X. Huang, Y. Zhang, G. Lu, L. Chou et al., Unveiling the activity and stability origin of BiVO_4_ photoanodes with FeNi oxyhydroxides for oxygen evolution. Angew. Chem. Int. Ed. **59**, 18990-18995 (2020). <https://doi.org/10.1002/anie.202008198>
16. J.-B. Pan, B.-H. Wang, J.-B. Wang, H.-Z. Ding, W. Zhou et al., Activity and stability boosting of an oxygen-vacancy-rich BiVO_4_ photoanode by NiFe-MOFs thin layer for water oxidation. Angew. Chem. Int. Ed. **60**, 1433-1440 (2021). <https://doi.org/10.1002/anie.202012550>
17. B. Zhang, S. Yu, Y. Dai, X. Huang, L. Chou et al., Nitrogen-incorporation activates NiFeO_x_ catalysts for efficiently boosting oxygen evolution activity and stability of BiVO_4_ photoanodes. Nat. Commun. **12**, 6969 (2021). <https://doi.org/10.1038/s41467-021-27299-0>
18. J. Cui, M. Daboczi, M. Regue, Y.-C. Chin, K. Pagano et al., 2D bismuthene as a functional interlayer between BiVO_4_ and NiFeOOH for enhanced oxygen-evolution photoanodes. Adv. Funct. Mater. **32**, 2207136 (2022). <https://doi.org/10.1002/adfm.202207136>
19. Z. Zhang, X. Huang, B. Zhang, Y. Bi, High-performance and stable BiVO_4_ photoanodes for solar water splitting via phosphorus–oxygen bonded FeNi catalysts. Energy Environ. Sci. **15**, 2867 (2022). <https://doi.org/10.1039/D2EE00936F>
20. Y. Zhang, L. Xu, B. Liu, X. Wang, T. Wang et al., Engineering BiVO_4_ and oxygen evolution cocatalyst interfaces with rapid hole extraction for photoelectrochemical water splitting. ACS Catal. **13**, 5938-5948 (2023). <https://doi.org/10.1021/acscatal.3c00444>
21. X. Li, J. Wu, C. Dong, Y. Kou, C. Hu et al., Boosting photoelectrocatalytic oxygen evolution activity of BiVO_4_ photoanodes via caffeic acid bridged to NiFeOOH. Appl. Catal. B-Environ. Energy **353**, 124096 (2024). <https://doi.org/10.1016/j.apcatb.2024.124096>
22. X. Chang, T. Wang, P. Zhang, Y. Wei, J. Zhao et al., Stable aqueous photoelectrochemical CO_2_ reduction by a Cu_2_O dark cathode with improved selectivity for carbonaceous products. Angew. Chem. Int. Ed. **55**, 8840-8845 (2016). <https://doi.org/10.1002/anie.201602973>
23. G. Magesh, E.S. Kim, H.J. Kang, M. Banu, J.Y. Kim et al., A versatile photoanode-driven photoelectrochemical system for conversion of CO_2_ to fuels with high faradaic efficiencies at low bias potentials. J. Mater. Chem. A **2**, 2044 (2014). <https://doi.org/10.1039/C3TA14408A>
24. W. Liu, Y. Yang, F. Zhan, D. Li, Y. Li et al., Ultrafast fabrication of nanostructure WO_3_ photoanodes by hybrid microwave annealing with enhanced photoelectrochemical and photoelectrocatalytic activities. Int. J. Hydrogen Energy **43**, 8770-8778 (2018). <https://doi.org/10.1016/j.ijhydene.2018.03.082>
25. M. Li, P. Li, K. Chang, H. Liu, X. Hai et al., Design of a photoelectrochemical device for the selective conversion of aqueous CO_2_ to CO: using mesoporous palladium-copper bimetallic cathode and hierarchical ZnO-based nanowire array photoanode. Chem. Commun. **52**, 8235-8238 (2016). <https://doi.org/10.1039/C6CC03960J>
26. B. Liu, T. Wang, S. Wang, G. Zhang, D. Zhong et al., Back-illuminated photoelectrochemical flow cell for efficient CO_2_ reduction. Nat. Commun. **13**, 7111 (2022). <https://doi.org/10.1038/s41467-022-34926-x>
27. Y. Zhang, W. Luc, G.S. Hutchings, F. Jiao, Photoelectrochemical carbon dioxide reduction using a nanoporous Ag cathode. ACS Appl. Mater. Interfaces **8**, 24652-24658 (2016). <https://doi.org/10.1021/acsami.6b09095>
28. B. Zhao, X. Huang, Y. Ding, Y. Bi, Bias-free solar-driven syngas production: a Fe_2_O_3_ photoanode featuring single-atom cobalt integrated with a silver-palladium cathode. Angew. Chem. Int. Ed. 62, e202213067 (2023). <https://doi.org/10.1002/anie.202213067>
29. H. Zhang, P. Zhang, J. Zhao, Y. Liu, Y. Huang et al., The hole-tunneling heterojunction of hematite-based photoanodes accelerates photosynthetic reaction. Angew. Chem. Int. Edit. **60**, 16009-16018 (2022). <https://doi.org/10.1002/anie.202102983>
30. J.H. Kim, G. Magesh, H.J. Kang, M. Banu, J.H. Kim et al., Carbonate-coordinated cobalt co-catalyzed BiVO_4_/WO_3_ composite photoanode tailored for CO_2_ reduction to fuels. Nano Energy **15**, 153-163 (2015). <https://doi.org/10.1016/j.nanoen.2015.04.022>
31. L.-X. Liu, J. Fu, L.-P. Jiang, J.-R. Zhang, W. Zhu et al., Highly Efficient photoelectrochemical reduction of CO_2_ at low applied voltage using 3D Co-Pi/BiVO_4_/SnO_2_ nanosheet array photoanodes. ACS Appl. Mater. Interfaces **11**, 26024-26031 (2019). <https://doi.org/10.1021/acsami.9b08144>
32. Z. Zhang, X. Huang, Y. Bi, High-efficiency and stable syngas production by coupling NiFe-BiVO_4_ photoanodes with AgO_x_/Ag cathodes. Appl. Catal. B-Environ. Energy **349**, 123894 (2024). <https://doi.org/10.1016/j.apcatb.2024.123894>
33. F. Urbain, P. Tang, N.M. Carretero, T. Andreu, L.G. Gerling et al., A prototype reactor for highly selective solar-driven CO_2_ reduction to synthesis gas using nanosized earth-abundant catalysts and silicon photovoltaics. Energy Environ. Sci. **10**, 2256 (2017). <https://doi.org/10.1039/C7EE01747B>
34. A. Sacco, R. Speranza, U. Savino, J. Zeng, M.A. Farkhondehfal et al., An integrated device for the solar-driven electrochemical conversion of CO_2_ to CO. ACS Sustainable Chem. Eng. **8**, 7563-7568 (2020). <https://doi.org/10.1021/acssuschemeng.0c02088>
35. U. Kang, S.K. Chio, D.J. Ham, S.M. Ji, W. Choi et al., Photosynthesis of formate from CO_2_ and water at 1% energy efficiency via copper iron oxide catalysis. Energy Environ. Sci. **8**, 2638-2643 (2015). <https://doi.org/10.1039/C5EE01410G>
36. V. Andrei, G.M. Ucoski, C. Pornrungroj, C. Uswachoke, Q. Wang et al., Floating perovskite-BiVO_4_ devices for scalable solar fuel production. Nature **608**, 518-522 (2022). <https://doi.org/10.1038/s41586-022-04978-6>
37. Y.J. Jang, I. Jeong, J. Lee, J. Lee, M.J. Ko et al., Unbiased sunlight-driven artificial photosynthesis of carbon monoxide from CO_2_ using a ZnTe-based photocathode and a perovskite solar cell in tandem. ACS Nano **10**, 6980-6987 (2016). <https://doi.org/10.1021/acsnano.6b02965>
38. . Andrei, B. Reuillard, E. Reisner, Bias-free solar syngas production by integrating a molecular cobalt catalyst with perovskite-BiVO_4_ tandems. Nat. Mater. **19**, 189 (2020). <https://doi.org/10.1038/s41563-019-0501-6>
39. M. Rahaman, V. Andrei, C. Pornrungroj, D. Wright, J.J. Baumberg et al., Selective CO production from aqueous CO_2_ using a Cu_96_In_4_ catalyst and its integration into a bias-free solar perovskite-BiVO_4_ tandem device. Energy Environ. Sci. **13**, 3536 (2020). <https://doi.org/10.1039/D0EE01279C>
40. Q. Jia, S. Tanabe, I. Waki, Direct gas-phase CO_2_ reduction for solar methane generation using a gas diffusion electrode with a BiVO_4_:Mo and a Cu-In-Se photoanode. Chem. Lett. **47**, 436 (2018). <https://doi.org/10.1246/cl.171094>
